# Climate change scenarios across South-Kivu agroecological zones, Eastern D.R. Congo

**DOI:** 10.1038/s41598-026-50143-8

**Published:** 2026-05-03

**Authors:** Luc Cimusa Kulimushi, Jean M. Mondo, Janvier B. Bashagaluke, Aimé H. K. Bisimwa, Alphonse Z. Balezi, Irfan Ur Rashid, Pankaj Prasad, Jackson-Gilbert M. Majaliwa, Sudhir Kumar Singh, Charles Kahindo, Mansour Almazroui, Katcho Karume

**Affiliations:** 1 Doctoral School of Agroecology and Climate Sciences, Université Evangélique en Afrique (UEA), Bukavu, Democratic Republic of Congo; 2 Faculty of Agriculture, Université Catholique de Bukavu, Bukavu, Democratic Republic of Congo; 3 Centre Régional d’Etudes Interdisciplinaires Appliquées au Développement Durable (CEREIAD-UCB), Université Catholique de Bukavu, Bukavu, Democratic Republic of Congo; 4 Faculty of Agriculture and Environmental Sciences, Université Evangélique en Afrique (UEA), Bukavu, South-Kivu, Democratic Republic of Congo; 5 Department of Agriculture, Université Officielle de Bukavu (UOB), Bukavu, Democratic Republic of Congo; 6 Institut Supérieur de Techniques de Développement, ISTD, Kalehe, Democratic Republic of Congo; 7 Research and Development Division, Meteorological Department, Islamabad, 44000 Pakistan; 8 Geoinformatics Division, Department of Geography, Mahapurusha Srimanta Sankaradeva Viswavidyalaya, Nagaon, India; 9 RUFORUM, Makerere University, Kampala, Uganda; 10 Nehru Science Centre, K. Banerjee Centre of Atmospheric & Ocean Studies, IIDS, University of Allahabad, Prayagraj, 211002 Uttar Pradesh India; 11 Faculty of Sciences, Université Officielle de Bukavu (UOB), Bukavu, Democratic Republic of Congo; 12 Center of Excellence for Climate Change Research, Department of Meteorology, King Abdulaziz University, Jeddah, Saudi Arabia; 13 Climatic Research Unit, School of Environmental Sciences, University of East Anglia, Norwich, NR4 7TJ UK; 14 Centre de Recherche en Géothermie, (CRGeo), Bukavu, Democratic Republic of Congo

**Keywords:** CMIP6, Climate projections, Taylor Skill Score, Topographic gradients, SSP scenarios, Agroecological zones, Congo Basin, Climate sciences, Environmental sciences

## Abstract

Characterizing climate projections to capture agroecological heterogeneity and topographic gradients is essential for localized planning in complex terrains. This study evaluates historical trends and projected changes in temperature and precipitation across six agroecological zones (AEZs) in South-Kivu, Democratic Republic of the Congo under SSP2-4.5, SSP3-7.0, and SSP5-8.5 scenarios. We evaluated seven CMIP6 models using the Taylor Skill Score (TSS), selecting the three best-performing models per variable and AEZ to construct zone-specific multi-model ensembles. Bias correction was performed using Quantile Mapping (QM) with a two-fold cross validation design (1983–1998 and 1999–2014). Selected models demonstrated high ranking stability across independent historical windows, achieving a weighted mean stability index of 0.83 for precipitation, 0.94 for maximum temperature (Tmax) and 1.00 for minimum temperature (Tmin). For precipitation, BCC-CSM2-MR excelled in low-altitude zones, while CMCC-CM2-SR5 and MPI-ESM1-2-HR were more effective in high-altitude zones. MRI-ESM2-0 and GFDL-ESM4 performed best for temperature across most AEZs. Climate trajectories were analyzed for near-term (2026–2050), mid-term (2051–2075) and long-term (2075–2100) periods relative to a 1983–2014 baseline. Reported projections represent the ensemble mean, while inter-model spread is detailed in the results. Results indicate continuous warming across all AEZs, with Tmin increasing more rapidly than Tmax. Province wide for the near (long) term, Tmax increases range from 0.78 °C (1.64 °C), 0.82 °C (2.59 °C), to 0.87 °C (2.85 °C) under SSP2-4.5, SSP3-7.0, and SSP5-8.5; Tmin increases range from 1.08 °C (2.12 °C), 1.13 °C (3.12 °C), and 1.25 °C (3.46 °C), respectively. Precipitation projections reveal non-linear, AEZ-differentiated trajectories characterized by near-term reductions followed by a long-term transition toward wetting in several zones. The EMAZ exhibits persistent declines (-9.8%) and the THMAZ shows a late-century wetting (+ 21%) under SSP5-8.5. Furthermore, a shift in seasonal redistribution is projected, with the long rain season intensifying and the short rain season contracting. These findings characterize the range of plausible climate trajectories in South Kivu, providing a necessary evidence base for future risk-targeted adaptation planning in eastern DRC and comparable tropical mountain systems.

**Supplementary Information:**

The online version contains supplementary material available at 10.1038/s41598-026-50143-8.

## Introduction

Climate change remains one of humanity’s greatest challenges, profoundly influencing ecosystems and livelihoods worldwide^1^. Africa is among the most vulnerable regions and has warmed significantly over the past century, with temperatures projected to increase by 1.4–4.4 °C by 2100^2^. The Democratic Republic of the Congo (DRC) is identified as a high-risk country, ranking 167th in vulnerability and 179th in readiness globally^3^. This vulnerability is primarily driven by a high dependence on rainfed agriculture, widespread poverty, and recurring conflicts, particularly in the eastern DRC^4–6^.

Observational evidence confirms the early signature of these changes. Temperatures are rising, rainfall is becoming increasingly erratic, and extreme events are intensifying^6–9^. Indeed, over half of Congolese farmers report declining rainfall, while 94% observe rising temperatures^10^. These shifts have already triggered major yield losses^[,7,8^, pest outbreaks^11,12,13^, and rising prevalence of climate-sensitive disease^14^. Beyond immediate agricultural impacts, climate change is restructuring the hydrological cycle, jeopardizing long-term water security^15–19^. Projected warming is expected to intensify evapotranspiration and deplete soil moisture^20,21^, while a shift toward fewer but more intense rainfall events overwhelms infiltration capacity, amplifying surface runoff, flash floods, and landslide susceptibility^22–24^. In South Kivu, where topography range from 500 m to 3412 m a.s.l Mitumba Mountains, these hazards are already frequent^6,8,25–29^. Understanding how they will evolve requires climate projections that can resolve the spatial heterogeneity of this landscape.

Global Circulation Models (GCMs) are the primary tools for such projections^30–32^. GCMs employ mathematical equations to simulate complex interactions within the Earth’s climate system under various greenhouse gas (GHG) emission scenarios^33–35^. The Coupled Model Intercomparison Project Phase 6 (CMIP6) represents the current state of the art. CMIP6 offers important advances over its predecessor, featuring improved modeling of synoptic processes, and updated Shared Socioeconomic Pathways (SSPs)^33,34^. Despite these advances, there is still a lot of uncertainty associated with each of the underlying GCMs to accurately replicate various regions across the globe^36–39^. These distortions are particularly acute in topographically complex regions where coarse-resolution models fail to capture mesoscale convective dynamics and orographic moisture transport^39–42^. Bias correction therefore becomes a physical imperative to align model outputs with observed regional signals and ensure scientifically robust projections^36,38,39,43^. This approach narrows uncertainty and ensures that the resulting output is physically consistent with observed regional climate signal, providing physically justified projections for impact assessments^44–46^.

Even with bias-corrected outputs, the spatial scale determines practical utility. National and continental-level assessments of CMIP6 performance across Africa have identified broad climatic patterns^2,14,47,48^, but these macro-scales may systematically mask the localized variability that triggers biophysical thresholds in agriculture and hydrology^32,49^. While these previous studies have contributed valuable insights for larger regions or specific time resolutions, a more detailed and localized examination of future climatic dynamics at local scale is required. Agroecological zones (AEZs) provide a framework to bridge this gap^50,51^. This approach partitions landscapes into relatively homogeneous units based on climatic, topographic and edaphic characteristics^52^, ensuring that projections are directly applicable to agricultural adaptation and water resource policy^50,51^. In South Kivu, six distinct AEZs have been identified^53^, and reveal contrasting climate sensitivity, and agricultural potentials. Characterizing these zone-specific trajectories is fundamental for anticipating hydrological responses, and developing targeted disaster risk reduction (DRR) strategies. To the author’s knowledge, the capacity of the recent CMIP6 models to capture fine-scale fluctuations in precipitation and temperature across South Kivu’s AEZs has not been thoroughly documented.

This study addresses these gaps by providing a detailed, CMIP6-based climate assessment tailored to the six AEZs of South-Kivu. Specifically, the study pursues three objectives: (i) to analyze historical trends in temperature and precipitation from 1983 to 2014 and explore their statistical correlations across the six AEZs; (ii) to evaluate the performance of seven CMIP6 models and identify the top performers for each AEZ and variable; and (iii) to examine projected changes under three SSP scenarios across near-term (2026–2050), mid-term (2051–2075), and long-term (2075–2100) horizons. Integrating the future climatic conditions within the geographic reality of AEZs, this work establishes the essential evidence base for future water management policies and agricultural resilience in the DRC.

## Materials and methods

### Description of South-Kivu’s AEZs

**Fig. 1:**
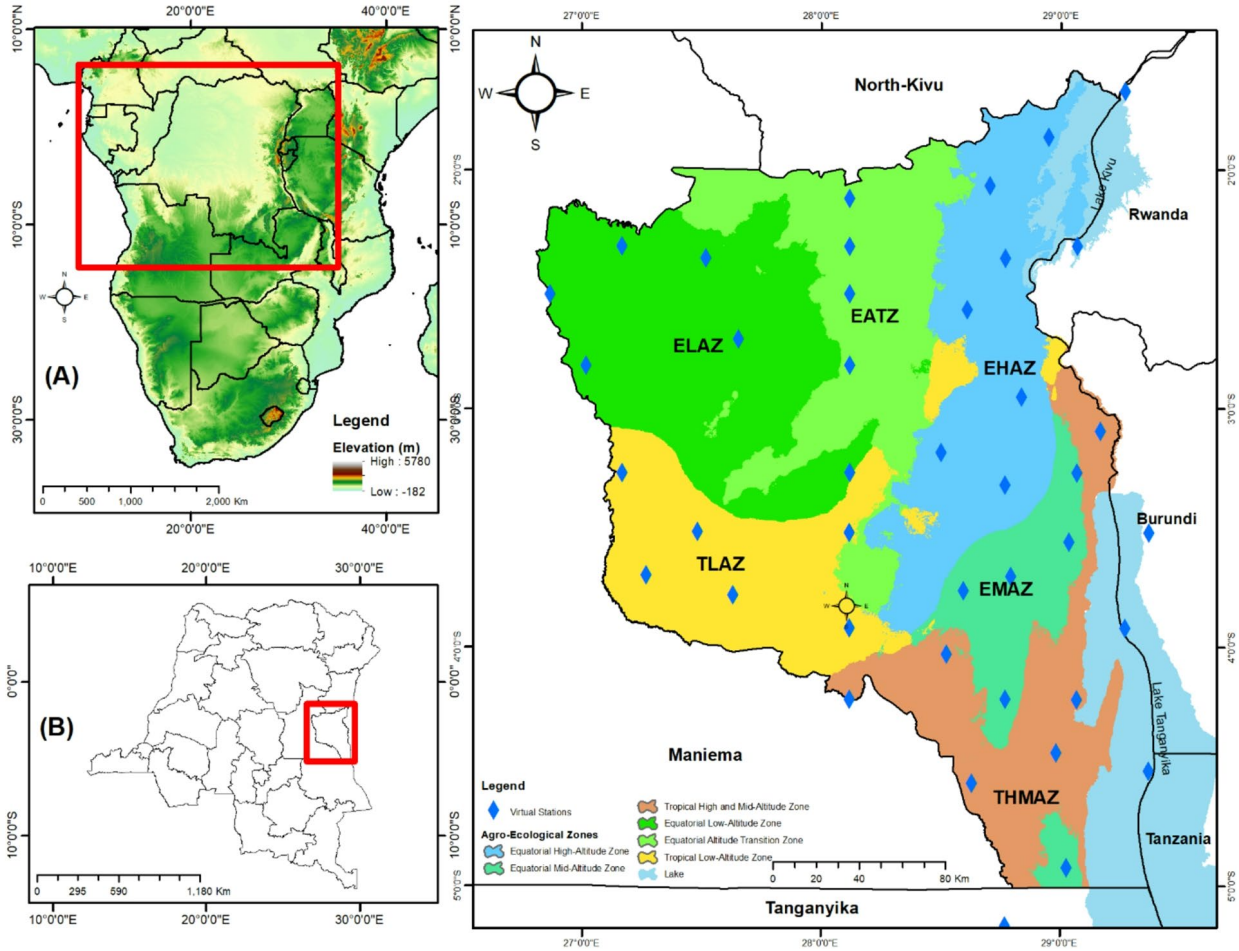
Location of the Study area. (**A**) Middle Africa with DRC highlighted (red box), (**B**) DRC with South Kivu highlighted among 26 provinces, (**C**) Agroecological zones of South Kivu.

South-Kivu Province is located in the eastern DRC between 1.5836° and 5.0103° S and 26.8106° and 29.3890° E (Fig. 1). The province covers ~ 65 000 km2 (approximately 2.73% of the DRC) and features important geoclimatic diversity ranging from floodplains to hilly landscapes. This study focused on six AEZs: the Equatorial High-Altitude Zone (EHAZ), Equatorial Mid-Altitude Zone (EMAZ), Equatorial Altitude Transition Zone (EATZ), Equatorial Low Altitude Zone (ELAZ), Tropical High and Mid-Altitude Zone (THMAZ), and Tropical Low Altitude Zone (TLAZ), adapted from Kulimushi et al.^53^. The spatial distribution of these AEZs is illustrated in Fig. 1, while Table 1 details their respective elevation ranges, mean annual precipitation, mean temperatures, and climatic classifications. For a detailed assessment of climatic variation within the six zones, Figure S1 provides box-and-whisker plots of these variables.

**Table 1:** Topographic and climatic characteristics of South-Kivu AEZs. (Adapted from Kulimushi et al. 2026)

AEZs	Elevation ranges		Mean annual rainfall (mm)	Mean annual temperature (°C)	Length of the growing period (days)	Climatic classification
	Minimum (m)	Maximum (m)				
EHAZ	1078.03	3411.56	1671.55	19.98	210	Wet subhumid
EMAZ	1132.83	3255.89	1472.05	19.60	158	Dry subhumid
EATZ	653	1955.06	2383.23	23.15	263	Very humid equatorial
ELAZ	504.28	1278.91	2199.90	25.83	257	Humid equatorial
THMAZ	644.74	2094.09	1204.41	25.04	144	Tropical dry subhumid
TLAZ	533.12	1654.28	1788.84	24.89	202	Tropical wet subhumid

EHAZ: Equatorial High-Altitude Zone (EHAZ), EMAZ: Equatorial Mid-Altitude Zone, THMAZ: Tropical High and Mid-Altitude Zone, ELAZ: Equatorial Low Altitude Zone, EATZ: Equatorial Altitude Transition Zone and TLAZ: Tropical Low Altitude Zone.

- EHAZ:

- Geographic coordinates: EHAZ is located between 1.6195°-3.9831° S, and 28.0095°-29.1554° E.
- Climate: This zone is located in the central Mitumba Mountains and encompasses the high-altitude portion of Kahuzi Biega National Park (KBNP). It features an average elevation of 1980 m above the sea level (a.s.l), peaking at 3412 m. The region is defined by a wet sub-humid climate, featuring a mean annual temperature of 19.98 °C, an average annual rainfall of 1671.8 mm, and a 210-day length of the growing period (LGP).

- EMAZ:

- Geographic coordinates: EMAZ is situated between 3.0022°-5.0090° S, and 28.2106°-29.1134° E.
- Climate: This zone extends the Mitumba Mountains to the southeast, and encompasses the high-altitude portion of Itombwe Natural Reserve (INR). It features an average elevation of 2148 m a.s.l, peaking at 3256 m. It is characterized by a dry sub-humid climate with a mean annual temperature of 19.6 °C and an average annual rainfall of 1472.05 mm. The region is defined by a 158-day LGP and high rainfall variability, resulting in pronounced wet/dry and warm/cool seasonal cycles.

- EATZ:

- Geographic coordinates: EATZ spans between 1.8661°-3.8677° S, and 27.3750°-28.6475° E.
- Climate: This zone represents the intermediate belt between equatorial lowlands and highlands, encompassing the larger medium-altitude portion of KBNP. It features an average elevation of 1,188.63 m, peaking at 1,955 m. The region is characterized by a very humid equatorial climate with a mean annual temperature of 23.15 °C, average annual rainfall of 2383.23 mm, and a 263-day LGP.

- ELAZ:

- Geographic coordinates: ELAZ spans between 2.0991°-3.4791° S and 26.8109°-28.4579° E.
- Climate: The zone extends from the Congo Basin rainforest in the east, forming a lowland equatorial region with a mean elevation of 891.6 m a.s.l, and a peak of 1278 m. It is characterized by a humid equatorial climate with a mean annual temperature of 25.83, and an average annual rainfall of 2199.90 mm. The region features a 257-day LGP.

- THMAZ:

- Geographic coordinates: THMAZ is situated between 2.7320°-5.0120° S, and 28.0166°-29.2638° E.
- Climate: The zone is located in the southeastern part of the province, featuring a mean elevation of 1369.4 m a.s.l, and peaking at 2094.09 m. It exhibits a tropical dry subhumid climate characterized by a mean annual temperature of 25.04 °C, and an average annual rainfall of 1204.41 mm. With 144-day LGP, the region experiences high seasonal variability, posing challenges related to water scarcity and drought stress.

- TLAZ:

- Geographic coordinates: TLAZ spans between 2.6820°-4.1284° S, and 27.0864°-29.0343° E.
- Climate: This zone is situated in the southwestern lowlands of the province, serving as a transition between the equatorial lowlands and tropical mid-to-high altitudes. It features an average elevation of 1093.7 m, peaking at 1654.28 m. The region is characterized by a tropical wet subhumid climate, with a mean annual temperature of 24.89 °C, and an average annual rainfall of 1801.7 mm. It is defined by a 202-day LGP, and experiences moderate seasonal variability.

### Observation dataset

Bias correction of GCMs requires substantial observational records to capture decadal climate variability^54,55^. In the DRC, the scarcity of ground station data and inconsistent spatiotemporal coverage limit conventional bias correction approaches^14^. Consequently, we utilized satellite-derived products as the best available alternative for this data-sparse region^14,56,57^. Daily precipitation data were obtained from the Climate Hazards Group InfraRed Precipitation with Stations (CHIRPS), at a 0.05° resolution, while surface air temperature data were derived from the Climate Hazards Group InfraRed Temperature with Stations (CHIRTS) at the same resolution. CHIRPS integrates satellite infrared estimates with in situ observations^58^, and CHIRTS combines CHRITSmax with ERA5 reanalysis^58^.

Both datasets provide continuously daily records from 1981 to present; thus, the historical period spanning 1983–2014 was selected as the baseline for this study. While these products are among the most robust evidence available for data-sparse regions^59–63^, and the DRC^14,56^, we acknowledge that the satellite derived products carry uncertainties. Inherent uncertainties including potential systematic underestimation of orographic precipitation, the smoothing of fine scale thermal heterogeneity^60,64^, and temporal inhomogeneities^65^, necessary propagate into the study’s total uncertainty budget. These factors primarily influence the absolute calibration of the bias-correction transfer functions and the magnitude of performance metrics, necessitating an interpretation of the resulting projections within a relative risk framework.

### Global climate models

CMIP6 uses a range of climate models and SSPs to project future climate change, integrating socioeconomic narratives with specific radiative forcing targets^34,66^. The IPCC Sixth Assessment Report outlines five core scenarios^67^. These include sustainable development (SSP1), middle-of-the-road development (SSP2-4.5), regional rivalry (SSP3-7.0), and fossil-fuel development (SSP5-8.5). The description of these scenarios are detailed in the IPCC Sixth Assessment Report^67^.

This study evaluates climate trajectories under SSP2-4.5, SSP3-7.0, and SSP5-8.5 scenarios^54^. These scenarios were selected to capture a plausible range of intermediate to high-end climate outcomes, providing a conservative basis for risk-averse adaptation planning in the highly vulnerable South Kivu region. While SSP1-2.6 represents an ambitious mitigation pathway consistent with the Paris Agreement 1.5–2 °C targets, it was excluded to prioritize scenarios that reflect plausible high-impact trajectories aligned with recent global emission trends and current policy baselines^14,54^. This selection ensures that localized adaptation strategies for the DRC are robust against a wide spectrum of potential warming levels^56,68^, focusing on the scenarios that pose the most significant challenges to agricultural and hydrological resilience^34,66^.

Future projections were derived from a performance-based selection of seven CMIP6 GCMs (Table 2). While larger ensembles are available, we selected a subset based on data availability and regional performance skill in capturing moisture transport and orographic precipitation in Central and East Africa^2,47,49,56,68^. We adopted a fitness-for-purpose selection strategy, prioritizing models with high horizontal resolution to better resolve the complex topography of South-Kivu^56^. To account for model uncertainty and structural dependence, our selection framework followed the criteria established by Adeyeri et al.^37^ and Knutti et al.^69^. We ensured that our ensemble represents distinct international modeling centers with diverse atmospheric cores and physics parameterizations. We deliberately avoided sister models and multiple versions of the same model structure to prevent the over-representation of specific biases, thereby maximizing the structural independence of the ensemble. This approach ensures that the multi-model ensemble captures a representative range of equilibrium climate sensitivities and internal variability^49^. Finally, to maintain internal consistency, the r1i1p1f1 variant was used for all models. Simulated historical (1970–2014) and future (2015–2100) datasets were retrieved from the ESGF archive (https://esgf-node.llnl.gov/projects/cmip6/).

**Table 2:** Reference of selected CMIP6 dataset for the study and availability of data with respect to maximum (Tmax) and minimum (Tmin) temperatures and precipitation as well as spatial resolution.

Model	Institution	Country	Tmax	Tmin	Precip	Resolution	Vibrant level	Key references
AWI-CM-1-1-MR	Alfred Wegener Institute, Helmholtz Centre for Polar and Marine Research, Germany	Germany	✓	✓	o	0.9° × 0.9°	r1i1p1f1	Semmler et al. (2018)
BCC-CSM2-MR	Beijing Climate Center, China Meteorological Administration, China	China	o	o	✓	1.1° × 1.1°	r1i1p1f1	Wu et al. (2019)
CMCC-CM2-SR5	Fondazione Centro Euro-Mediterraneo sui Cambiamenti Climatici, Italy	Italy	o	o	✓	1.3° × 0.9°	r1i1p1f1	Fogli et al. (2020)
GFDL-ESM4	NOAA Geophysical Fluid Dynamics Laboratory, USA	USA	✓	✓	✓	1.3° × 1.0°	r1i1p1f1	Held et al. (2019)
MIROC6	Japan Agency for Marine-Earth Science and Technology, Japan	Japan	✓	✓	✓	1.4° × 1.4°	r1i1p1f1	Tatebe et al. (2019)
MPI-ESM1-2-HR	Max Planck Institute for Meteorology, Germany	Germany	✓	✓	✓	0.9° × 0.9°	r1i1p1f1	Gutjahr et al. (2019)
MRI-ESM2-0	Meteorological Research Institute, Japan	Japan	✓	✓	✓	1.1° × 1.1°	r1i1p1f1	Yukimoto et al. 2019)

## Methods of data analysis

### Data quality assessment

CHIRPS and CHIRTS dataset quality was assessed using the ClimPact2 package in R software^70^ to identify potential data issues. Quality control checks included: (i) identification of negative precipitation values, (ii) detection of temperature values exceeding physically plausible ranges for the region (Tmin < 5 °C or Tmax > 45 °C), and (iii) assessment of temporal consistency in daily values. Following quality control, homogeneity analysis was performed to detect and correct temporal discontinuities that could introduce artificial trends^5,25,27^. For precipitation data, the RHtests_dlyPrcp package was applied^71^, while RHtestsV4 was used for temperature data. These tests employ non-parametric change-point detection to identify significant breakpoints in time series^72^.

Quality control results revealed no missing values across the 1983–2014 period in any AEZ. For precipitation, no negative values were detected, but temporal inconsistency checks identified ˂ 0.5% of days per zone with potential spurious values (daily precipitation > 200 mm/day). These flagged values were retained but monitored during subsequent analyses. For temperature, implausible values were rare (< 1.5% of records for Tmax, < 0.1% for Tmin). These temperature values were replaced with linearly interpolated values from adjacent days to maintain data continuity while removing obvious sensor errors.

Homogeneity test results using Pettitt test revealed distinct patterns across variables and AEZs (Table S1). For precipitation, no statistically significant breakpoints were detected in any of the six AEZs (all p ˃ 0.05), thus required no further adjustment. For Tmax significant breakpoints were detected in all six AEZs. Breakpoints occurred in 1993 for EHAZ, EMAZ, and THMAZ (p ˂ 0.004), and 1995 for ELAZ, EATZ, and TLAZ (p ˂ 0.0017). Shift magnitudes ranged from + 0.43 °C to 0.49 °C across zones. For Tmin, significant breakpoints were detected only in ELAZ (2006; *p* = 0.023; shift magnitude = + 0.306 °C) and EATZ in 1990 (*p* = 0.009; shift magnitude = + 0.382 °C), while the remaining four AEZs showed no significant discontinuities.

All detected discontinuities were adjusted using the quantile-matching algorithm implemented in RHtests, which corrects mean shifts while preserving variance and the distribution of extremes^71,72^. For precipitation, no adjustment was necessary as no breakpoints were detected across any AEZ. For Tmax, despite systematic breakpoints detected in all six AEZs, significant increasing trends persisted post-adjustment across all zones, with annual Tmax trends ranging from + 0.015 °C yr^− 1^ to + 0.016 °C yr^− 1^ (*p* < 0.01). Here, the homogeneity corrections removed abrupt shifts (magnitude + 0.428 to + 0.494 °C) while preserving the underlying gradual warming signal, demonstrating that the observed Tmax warming represents a real climatic trend rather than a data artifact. For Tmin, the two zones with detected breakpoints showed modest trend reversal post-adjustment (− 0.0064 to + 0.0043 °C yr^− 1^ in ELAZ; +0.0043 to − 0.0065 °C yr^− 1^ in EATZ), but neither was statistically significant before or after adjustment (p ˃ 0.11).

To address the spatial scale mismatch between observations data and GCM grid points, we performed a spatial aggregation of the corrected observational data to the GCM grid cell centers. Rather than employing a single nearest-neighbor approach^68^, we utilized bilinear interpolation^73^ to compute a distance-weighted mean of the four nearest observed grid points surrounding each GCM cell center. This aggregation strategy ensures that the historical reference data are spatially representative of the specific grid cells used to project future climate. By deriving a localized neighborhood mean, we provide a stable climatic baseline for the bias-correction algorithms^68^, reducing artifacts that can arise when comparing point-scale observations to grid-scale simulations. While this method focuses on areal representativeness, we acknowledge that it does not explicitly incorporate sub-grid lapse-rate corrections for temperature or elevation-aware interpolation for precipitation. However, both CHIRPS and CHIRTS already integrate topographic signals through their respective station-blending and ERA5 reanalysis components, which partially account for orographic effects^27,56,60^. Furthermore, since our final analysis is conducted at the AEZ level, the zone-level aggregation naturally integrates across internal elevation gradients. This multi-step approach ensures that the drivers of the region’s hydrological processes are captured in a manner consistent with the resolution of the climate models, thereby providing a reliable foundation for subsequent impact assessments.

Seasonal aggregations were computed for three distinct seasons characteristic of South Kivu’s climate regime as adapted from Mondo et al.^5^. The long rainy season (Season A, September to January), short rainy season (Season B, February to May) and Dry season spans from June to August.

### Bias correction

Given the inherently coarse resolution of GCMs and their systematic errors in simulating local climate, bias correction is essential for reliable impact assessments. A wide range of adjustment methods has been document in the literature^45,74,75^. Among available techniques, Quantile Mapping (QM) has gained popularity for its superior performance in reducing systematic errors across the entire probability distribution^76^. Furthermore, its computational efficiency and ease of implementation make it one of the most widely adopted approaches^56,77–79^. QM is a post-processing technique that adjusts the statistical properties of raw GCM outputs to match the observed frequency distribution^76^, by establishing a transfer function between the cumulative distribution functions (CDFs) of the historical observations ($\:{F}_{o,h})$, and the historical GCM simulation ($\:{F}_{m,h})$. The transfer function is expressed as:

1 $$\:{\stackrel{\sim}{x}}_{m}\:\left(t\right)={F}_{o,h}^{-1}\left({F}_{m,h}\left[{x}_{m}\left(t\right)\right]\right)$$

Where $\:{x}_{m}\left(t\right)$ is the raw GCM output at time $\:t$, and $\:{\stackrel{\sim}{x}}_{m}\:\left(t\right)\:$is the bias-adjusted value. The subscript $\:h,\:m,\:and\:o$ denote the historical periods, the model, and the observed data, respectively. This transformation ensures that the corrected GCM outputs provide a statistically consistent baseline for climate impact assessments^76,77^.

To account for the distinct statistical properties of climate variables, we implemented a variable-specific QM approach applied independently to each GCM grid cell prior to spatial aggregation. This ensures that the unique topographic and climatic characteristics of individual grid locations were preserved before generating zone-representative means. This univariate approach specifically corrects marginal distributions, including the mean, variance, and higher-order moments^57^. While this method effectively reduces systematic biases in local frequency and intensity, it corrects climate variables independently, potentially disregarding inter-variable dependencies and correlations as stated by Adeyeri et al.^80^. It is nevertheless designed to preserve the underlying temporal structure, and the raw model’s relative climate change signal. By maintaining the rank correlation of the raw model, the climate simulations remain intact, as discussed by Bannister et al.^81^.

For daily precipitation, we utilized Parametric Quantile Mapping (PQM) based on a Gamma distribution to account for its skewed and non-negative nature^57,82^. A wet-day threshold of 0.1 mm/day was applied to eliminate the GCM drizzle effect, consistent with previous study^83^. Empirical Quantile Mapping (EQM)^46,84–86^ was applied to temperature as a non-parametric method to match observed and simulated CDFs. To evaluate the predictive skill and stability of the model screening workflow, we implemented a symmetric two-fold cross-validation design. In Fold 1, QM was calibrated on 1983–1998 and evaluated on the withheld 1999–2014 period; in Fold 2 the design was reversed. While this out-of-sample validation provides confidence in the model selection framework, the final QM transfer functions used for future projections were re-calibrated on the full 1983–2014 record. This maximizes the observational sample size and the representation of climatic extremes^85^, ensuring that most stable statistical baseline for long-term projections, albeit while accepting the inherent epistemic risks associated with final stage recalibration.

To address potential QM-induced distortion of the climate change signal, a common critique of the stationarity assumption^85,87,88^, we conducted a signal preservation analysis for the 95th percentile of daily precipitation (Q95) and wet days frequency. For each AEZ, we quantified the relative change in both metrics between the historical period and the future period for both raw and QM corrected series. The QM-corrected distributions closely tracked observed values across the full upper tail (Figure S2). For Q95, absolute deviations between raw and corrected signals remained below 0.72% points (pp) across all 18 AEZs-scenario combinations, with the sign of change preserved in all cases (Figures S3-S4). For wet-day frequency, differences remained below 0.31 pp. Collectively, these results confirm that PQM framework preserves the externally forced signal across the full forcing range, while the MME approach further attenuates non-stationary biases^46,77–79,84,86^.

### Model performance evaluation

Model performance was evaluated using Taylor diagrams and Taylor skill score (TSS). Developed by Taylor^89^, Taylor diagrams visually compare simulations with observations using the correlation coefficient (r) standard deviation (SD), and centered root mean square difference (RMSD)^31,90^. The r measures temporal pattern agreement between simulated and observed values, SD quantifies variability relative to observations, and RMSD measures overall prediction error magnitude. Strong model performance is indicated by SD close to the observed SD, r near 1, and RMSD near 0^91^.

The TSS integrates these three components into a composite performance metric, evaluating models based on their simultaneous ability to reproduce temporal patterns, climatological variability and minimize systematic deviations. TSS values range from 0 to 1, with values closer 1 indicating superior performance^90,91^. The mathematical formulations are expressed in Eqs. 2 and 3.

2 $$\:RMSD=\:\sqrt{{SD}_{obs}^{2}+{SD}_{sim}^{2}-2{SD}_{obs}{SD}_{sim}r}$$

3 $$\:TSS=\:\frac{{4\left(1+r\right)}^{4}}{{\left({SD}_{sim}/{SD}_{obs}+{SD}_{obs}/{SD}_{sim}\right)}^{2}{\left(1+{r}_{max}\right)}^{4}}$$

Where r is the correlation coefficient between simulated and observed data; $\:{SD}_{obs}$ and $\:{SD}_{sim}$ represent the SDs of simulated and observed data, respectively; $\:{r}_{max}$ represents the maximum attainable correlation which is 1.

To validate the TSS-based evaluation framework, we calculated complementary performance metrics including Percent Bias (PBIAS)^57^, and Root Mean Square Error (RMSE)^56^. PBIAS (Eq. 4) measures the average bias tendency, while RMSE (Eq. 5) measure error magnitudes. All metrics were calculated at monthly scale. It is worth noting that, these metrics were derived from the out-of-sample validation period of the two-fold cross validation.

4 $$\:PBIAS=\frac{{\sum\:}_{i=1}^{n}({X}_{oi}-{X}_{si})}{{\sum\:}_{i=1}^{n}\left({X}_{oi}\right)}\:\times\:100$$

5 $$\:RMSE=\sqrt{\frac{1}{n}\sum\:_{i=1}^{n}{({X}_{si}-{X}_{oi})}^{2}}$$

Where $\:{X}_{oi}$ is the observed data on month $\:i$, $\:{X}_{si}$ is the simulated data on month $\:i$, $\:{\stackrel{-}{X}}_{oi}$ oi is the average measured value during the study period, and n is the total number of the observed data.

Based on the out-of-sample TSS rankings, the three highest-performing GCMs for each variable within each AEZ were selected to construct a Multi-Model Ensemble (MME) using a simple ensemble mean with equal weighting. To ensure the reliability of this selection, we assessed the stability index (SI) across the validation folds. A GCM was considered stable if it consistently appeared in the Top 3 performing models across both validation periods for a specific variable and AEZ.

This multi-metric and cross-validated approach ensure the ensemble remains robust in convective-influenced zones, capturing both the seasonal cycle and the absolute magnitude of climate variables essential for impact assessment. The equal-weighting approach assumes that selected models contribute complementary information and helps avoid overconfidence that can arise from complex weighting schemes^32,47,49,92,93^. The TSS based MME framework aligns with established practices, effectively leveraging individual model strengths while minimizing the influence of model-specific biases and preventing the underlying climate change signal^30,56,57^.

### Trend detection analysis

The Mann–Kendall (MK) test^94^ was applied to detect monotonic trends in annual and seasonal aggregates for precipitation, Tmax, and Tmin time series across all the AEZs. The MK test is a non-parametric method that assesses the existence of statistically significant trends without assuming a normal distribution. MK analysis on both annual and seasonal aggregates. For a time series $\:X=\:\left\{{x}_{1},{x}_{2}\dots\:\:{x}_{n}\right\}$, the MK test statistic *S* is calculated using the following equations (Eqs. (6)–(9)):

6 $$S=\:\sum\:_{i=1}^{n-1}\sum\:_{j=i+1}^{n}sgn({x}_{j}-{x}_{i})$$

where $\:Xi$ and $\:Xj$ are the values of sequences $\:i$ and $\:j$ and where $\:n$ is the length of the time series.

where sgn is the sign function:

7 $$\:\:\:\:\:\:\:\:\:\:\:\:\:\:\:\:\:\:\:\:\:\:\:\:\:\:\:sgn\:({x}_{j}-{x}_{i})=\left\{\begin{array}{c}+1\:if\:{x}_{j}-{x}_{i}>0\\\:0\:if\:{x}_{j}-{x}_{i}=0\\\:-1\:if\:{x}_{j}-{x}_{i}\:<0\end{array}\right.$$

The variance of S is computed as:

8 $$\:\:\:\:\:\:\:\:\:\:\:\:\:\:\:var\left(s\right)=\frac{n\left(n-1\right)\left(2n+5\right)-\:{\sum\:}_{t}t(t-1)(2t+5)}{18}$$

where t denotes the extent of any given tie. The standardized test statistic Z is then:

9 $$\:\:\:\:\:\:\:\:\:\:\:\:\:\:\:\:\:\:\:\:\:\:\:\:\:\:Z=\left\{\begin{array}{c}\frac{S-1}{\sqrt{var\left(S\right)}}\:if\:S>0\\\:0\:if\:S=0\\\:\frac{S+1}{\sqrt{var\left(S\right)}}\:if\:S<0\end{array}\:\right.$$

Statistical significance was assessed at the 95% confidence level (α = 0.05). A positive Z value indicates an upward trend, whereas a negative value indicates a downward trend. Prior to trend analysis, we assessed the lag-1 autocorrelation ($\:{\rho\:}_{1}$) for all times series; the resulting coefficients remained low ($\:{\rho\:}_{1}\le\:0.25$), indicating negligible serial correlation. Consequently, following the recommendations of Von Storch^95^ and Yue et al.^96^, we applied no pre-whiting or variance correction to avoid introducing bias in the presence of weak autocorrelation. To mitigate potential errors arising from multiple testing across the six AEZs, the Benjamini-Hochberg procedure was applied to control the False Discovery Rate (FDR). The magnitude of detected trends was quantified using Sen’s slope estimator (Sen, 1968), calculated as the median of all pairwise slopes in the time series (Eq. 10):

10 $$\:\beta\:=median\:\left(\frac{{x}_{j}-{x}_{i}}{j-i}\right)for\:all\:i\le\:j$$

where $\:\beta\:$ represents the trend magnitude in mm yr^− 1^ for precipitation or °C yr^− 1^ for temperature.

### Correlation analysis

To assess the physical coupling between temperature and precipitation, Pearson correlation coefficients (r) were calculated to identify the linear relationship between annual daily average temperature and total annual precipitation for each AEZ using 1983–2014 timeframe. Annual scale aggregation was used to eliminate intra-annual seasonal cycles, ensuring the correlations specifically capture inter-annual climate feedbacks. For two variables X and Y with n paired observations, the correlation coefficient is (Eq. 11):

11 $$\:r=\:\frac{{\sum\:}_{i=1}^{n}\left({x}_{i}-\stackrel{-}{x}\right)({y}_{i}-\stackrel{-}{y})}{\sqrt{\sum\:_{i=1}^{n}{({x}_{i}-\stackrel{-}{x})}^{2}}\:\times\:\:\sqrt{\sum\:_{i=1}^{n}{({y}_{i}-\stackrel{-}{y})}^{2}}}$$

where $\:\stackrel{-}{x}$ and $\:\stackrel{-}{y}$ are the means X and Y, respectively. The coefficient ranges from − 1 (perfect negative correlation) to + 1 (perfect positive correlation), with values near 0 indicating no linear relationship.

Simple linear regression was applied to quantify the proportion of precipitation variance explained by temperature. The regression model takes the following form (Eq. 12):

12 $$\:y=mx+c$$

where *y* is the precipitation, *x* is the temperature, *m* is the slope, and *c* is the y-intercept. The coefficient of determination (R²) was calculated to assess the goodness of fit:

13 $$\:{R}^{2}=1-\frac{\sum\:_{i=1}^{n}{({y}_{i}-{\widehat{y}}_{i})}^{2}}{\sum\:_{i=1}^{n}{({y}_{i}-\stackrel{-}{y})}^{2}}$$

where $\:{\widehat{y}}_{i}$ represents the predicted values. R² values range from 0 to 1, with higher values indicating that temperature explains a greater proportion of precipitation variability. The correlation strength was classified as weak (*r* < 0.3), moderate (0.3 ≤ *r* < 0.7), or strong (*r* ≥ 0.7).

### Projected precipitation and temperature change analysis

Future annual and seasonal changes were analyzed at the AEZ scale under three SSP scenarios using MMEs. The absolute change (AC, Eq. (14)) was used for temperature (Tmax and Tmin) changes, and the percentage change (Eq. (15)) was used for precipitation. Anomalies were calculated relative to the baseline period. Under three scenarios, projections were evaluated for three periods: the near-term (2026–2050), mid-term (2051–2075), and long-term (2076–2100) periods.

14 $$\:AC=\left({T}_{sim}-{T}_{o}\right)$$

15 $$\:PC=\frac{{(P}_{sim}-{P}_{o})}{{P}_{o}}\:\times\:100$$

where $\:AC$ and $\:PC$ represent the absolute and percentage changes in the projection periods relative to the reference period. $\:{T}_{sim}$ and $\:{P}_{sim}$ are the temperature (Tmax and Tmin) and precipitation, respectively, in a specified projection period, whereas $\:{T}_{o}$ and $\:{P}_{o}$ are the temperature and precipitation, respectively, in the reference period. To evaluate the sensitivity of these results to ensemble membership, projections were additionally computed using an expanded Top-5 ensemble for each AEZ and variable. Differences ($\:\varDelta\:$) between the Top-3 and Top-5 means were assessed against robustness thresholds of $\:\pm\:2\%$ for precipitation and $\:\pm\:0.2^\circ\:C$ for temperature.

To evaluate the statistical significance of the seasonal deltas, a non-parametric bootstrapping procedure was implemented. For each AEZ and variable, 1,000 bootstrap resamples with replacement were generated from the projected seasonal series. The 95% confidence intervals (CI) were derived using the percentile method from the resulting distribution of means. A seasonal shift was considered statistically significant at the 95% level where the confidence interval did not cross the zero-change threshold. Seasons were defined following the bimodal rainfall regime of South Kivu as described by Mondo et al.^5^. Seasonal deltas represent the mean projected change aggregated across the full 2026–2100 projection period relative to the 1983–2014 baseline.

## Results

### Analysis of historical climatic variables

#### Observed precipitation

**Fig. 2:**
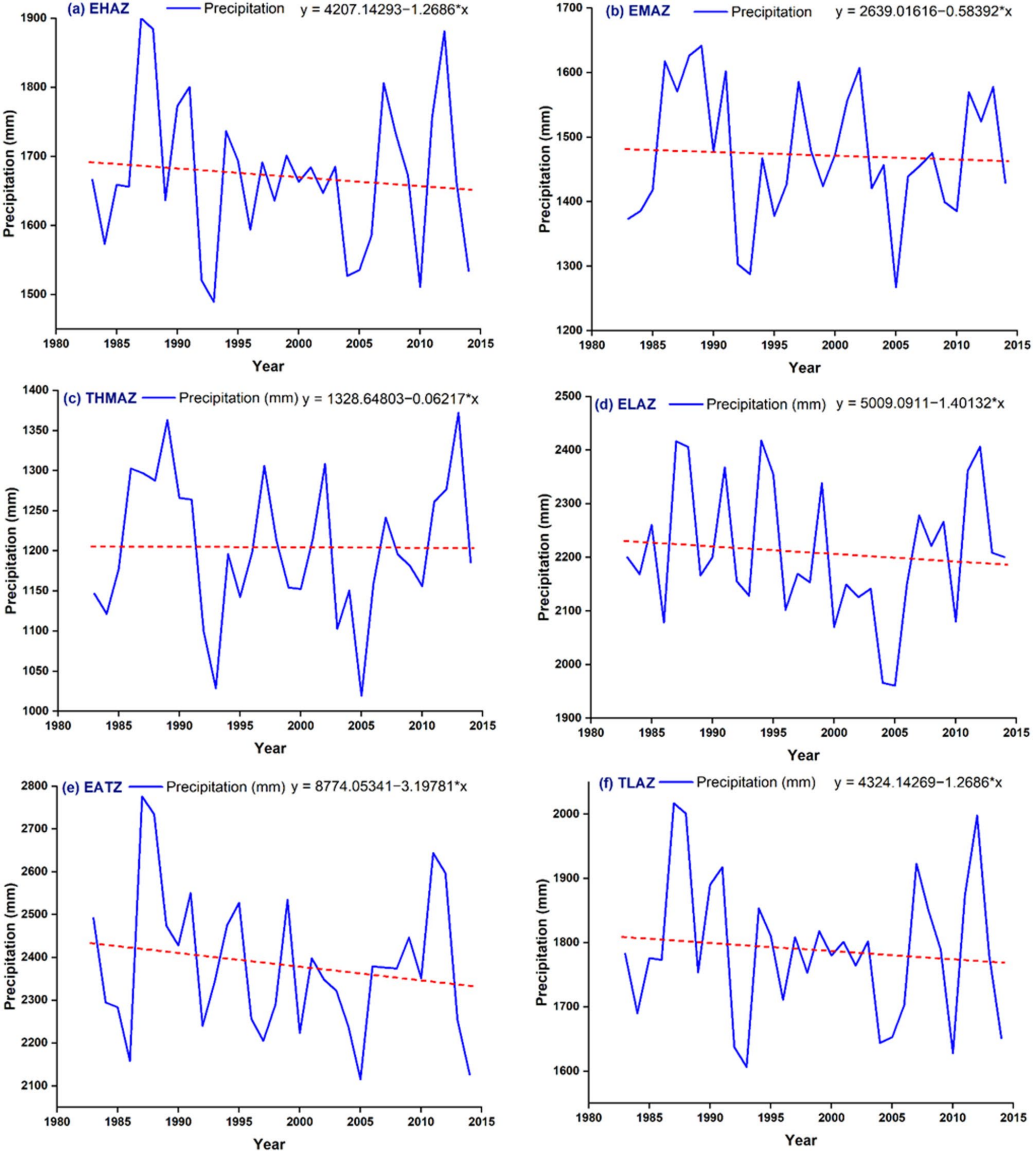
Observed annual precipitation in different agroecological zones (1983–2014). EHAZ: Equatorial High-Altitude Zone (EHAZ), EMAZ: Equatorial Mid-Altitude Zone, THMAZ: Tropical High and Mid-Altitude Zone, ELAZ: Equatorial Low Altitude Zone, EATZ: Equatorial Altitude Transition Zone and TLAZ: Tropical Low Altitude Zone.

Table 3 summarizes the Mann‒Kendall test results and Sen’s slope estimates, while Fig. 2 presents the historical annual precipitation variations (1983–2014). At the annual scale, precipitation trends were predominantly negative but statistically nonsignificant (p ˃ 0.05) in five of six AEZs. Annual Sen’s slope values ranged from − 0.436 mm yr^− 1^ in EMAZ to -4.518 mm yr^− 1^ in EHAZ. EHAZ was the only zone to exhibit a statistically significant decline (− 4.518 mm yr^− 1^, *p* = 0.043 at *p* = 0.05), while THMAZ exhibited a positive non-significant trend ((+ 0.295 mm yr^− 1^, *p* = 0.884).

**Table 3:** Mann-Kendall test and Sen’s slope estimator for temperatures and rainfall.

AEZs	Period	Precipitation			Tmax			Tmin		
		$\:\boldsymbol{\omega\:}$	$\:\boldsymbol{\gamma\:}$	$\:\boldsymbol{p}$	$\:\boldsymbol{\omega\:}$	$\:\boldsymbol{\gamma\:}$	$\:\boldsymbol{p}$	$\:\boldsymbol{\omega\:}$	$\:\boldsymbol{\gamma\:}$	$\:p$
EHAZ	Annual	**-0.004**	**-4.518**	**0.043**	**0.006**	**0.015**	**0.006**	0.002	0.005	0.355
	Season A	-0.004	-3.001	0.077	0.004	0.015	0.067	0.001	0.002	0.685
	Season B	-0.004	-3.372	0.054	0.003	0.018	0.089	0.001	0.004	0.593
	Dry season	0.001	0.219	0.662	**0.004**	**0.017**	**0.046**	0.001	0.003	0.506
EMAZ	Annual	0.000	-0.436	0.884	**0.005**	**0.015**	**0.010**	0.001	0.003	0.549
	Season A	0.000	-0.051	0.987	**0.005**	**0.016**	**0.026**	0.001	0.003	0.638
	Season B	-0.001	-0.377	0.808	0.002	0.010	0.277	0.000	0.001	0.833
	Dry season	0.000	-0.022	0.961	**0.004**	**0.017**	**0.029**	0.001	0.005	0.638
THMAZ	Annual	0.000	0.295	0.884	**0.006**	**0.015**	**0.006**	0.000	0.000	1.000
	Season A	0.000	0.062	0.935	**0.005**	**0.017**	**0.024**	0.000	0.000	0.987
	Season B	-0.001	-0.446	0.783	0.003	0.013	0.140	-0.001	-0.002	0.685
	Dry season	0.002	0.214	0.323	**0.005**	**0.018**	**0.013**	0.001	0.002	0.527
ELAZ	Annual	-0.003	-3.332	0.132	**0.005**	**0.016**	**0.008**	-0.002	-0.006	0.250
	Season A	0.000	-0.366	0.858	**0.004**	**0.015**	**0.036**	-0.001	-0.005	0.685
	Season B	**-0.004**	**-3.431**	**0.034**	0.004	0.014	0.062	0.001	0.004	0.593
	Dry season	0.001	0.724	0.486	**0.004**	**0.018**	**0.031**	**-0.005**	**-0.023**	**0.009**
EATZ	Annual	-0.002	-3.049	0.408	**0.006**	**0.016**	**0.003**	0.002	0.004	0.427
	Season A	-0.002	-1.568	0.390	**0.005**	**0.018**	**0.024**	0.001	0.003	0.638
	Season B	-0.003	-3.280	0.102	**0.004**	**0.014**	**0.043**	0.003	0.009	0.168
	Dry season	0.001	1.040	0.506	**0.004**	**0.016**	**0.031**	0.000	0.000	1.000
TLAZ	Annual	-0.001	-1.202	0.615	**0.006**	**0.015**	**0.005**	-0.001	-0.003	0.549
	Season A	0.002	0.889	0.446	**0.004**	**0.016**	**0.029**	-0.001	-0.004	0.506
	Season B	**-0.005**	**-2.350**	**0.022**	0.003	0.012	0.149	0.001	0.004	0.570
	Dry season	0.002	0.614	0.263	**0.005**	**0.018**	**0.019**	-0.003	-0.018	0.089

$\:\omega\:$: Mann–Kendal’s tau; $\:\gamma\:$: Sen’s slope; p: probability. EHAZ: Equatorial High-Altitude Zone (EHAZ), EMAZ: Equatorial Mid-Altitude Zone, THMAZ: Tropical High and Mid-Altitude Zone, ELAZ: Equatorial Low Altitude Zone, EATZ: Equatorial Altitude Transition Zone and TLAZ: Tropical Low Altitude Zone.

Seasonal patterns revealed more localized shifts. For the long rainy Season A, all AEZs showed non-significant trends (*p* > 0.077). In contrast, the short rainy Season B showed more evidence of change, with statistically significant declines recorded in ELAZ (− 3.431 mm yr^− 1^, *p* = 0.034) and TLAZ (− 2.350 mm yr^− 1^, *p* = 0.022). Notably, EHAZ showed a marginal decline in Season B (− 3.372 mm yr^− 1^, *p* = 0.054), just above the significance threshold. Trends during the Dry season were generally positive but remained statistically non-significant across all zones (*p* > 0.26) indicating that dry-season precipitation totals have remained stable.

#### Observed temperatures

**Fig. 3:**
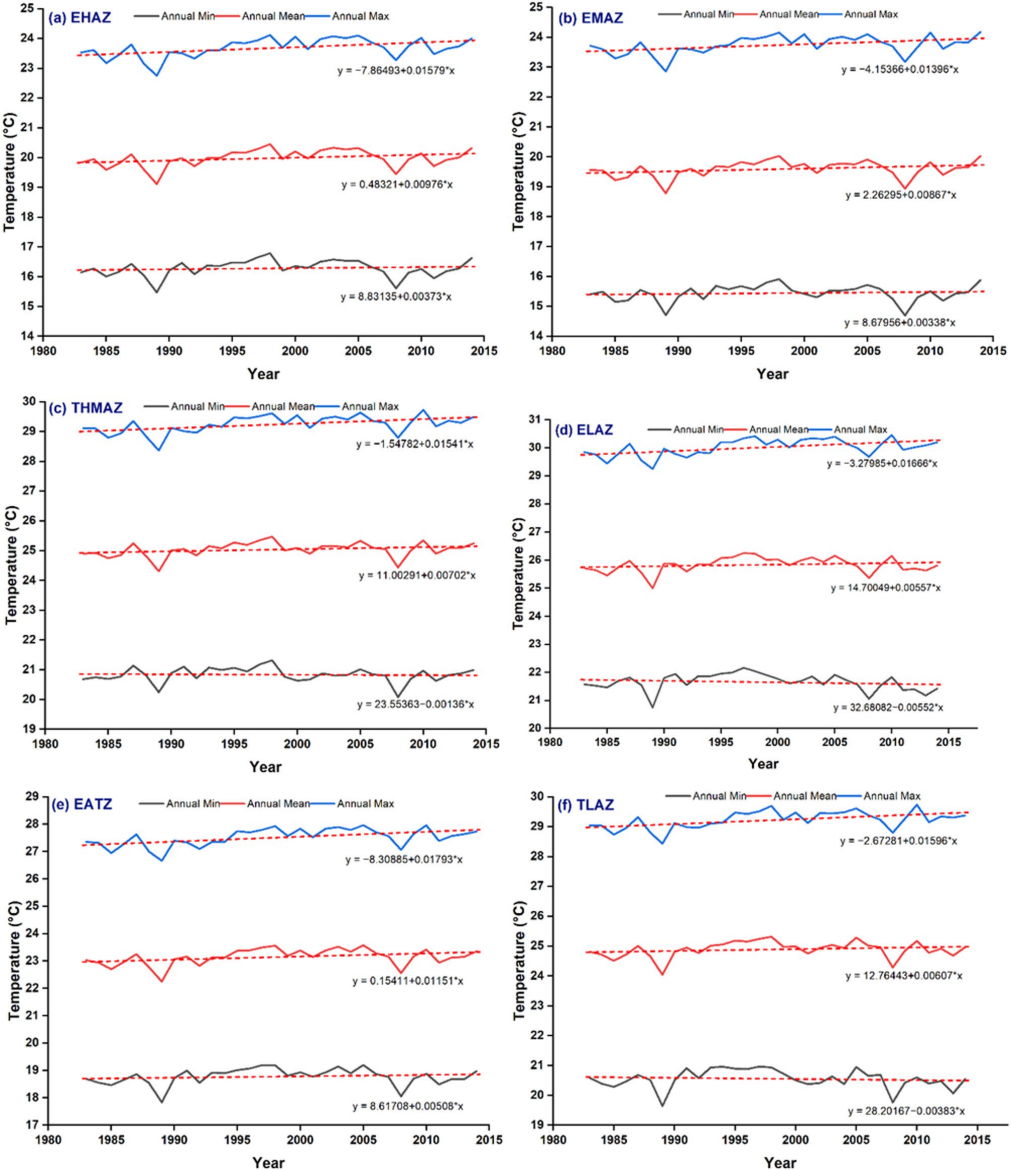
Observed annual daily average surface air temperature of in different Agroecological zones (1983–2014). EHAZ: Equatorial High-Altitude Zone (EHAZ), EMAZ: Equatorial Mid-Altitude Zone, THMAZ: Tropical High and Mid-Altitude Zone, ELAZ: Equatorial Low Altitude Zone, EATZ: Equatorial Altitude Transition Zone and TLAZ: Tropical Low Altitude Zone.

Figure 3 illustrates the historical annual variation in Tmin, average temperature (Tmean), and Tmax across the AEZs. Annual Tmax showed statistically significant warming trends (p ˂0.05) across all zones, with Sen’s slope estimates ranging from + 0.015 °C yr^− 1^ to + 0.016 °C yr^− 1^ (Table 3). In contrast, Tmin exhibited mixed and statistically non-significant trends (p ˃0.05), with values ranging from − 0.003 °C yr^− 1^ to + 0.005 °C yr^− 1^.

A spatially consistent warming signal was observed in EHAZ, EMAZ, and EATZ, where significant Tmax increases (+ 0.015 °C yr^− 1^ to + 0.016 °C yr^− 1^) were accompanied by non-significant Tmin increases (+ 0.003 °C to + 0.005 °C yr^− 1^). Conversely, THMAZ, TLAZ, and ELAZ exhibited an asymmetric warming pattern, characterized by significant increases in Tmax (+ 0.015 °C yr^− 1^ to + 0.016 °C yr^− 1^) but nearly stable or slightly declining Tmin (0.0 °C to -0.006 °C yr^− 1^).

Seasonally, the Dry season experienced the most significant warming, with significant Tmax increases in all zones, ranging from + 0.016 °C yr^− 1^ to + 0.018 °C yr^− 1^. Significant Tmax warming was also observed during Season A in EMAZ, THMAZ, and ELAZ, and during Season B in EATZ. However, Tmin trends remained generally weak and nonsignificant across most seasons and zones, with the notable exception of ELAZ, which showed a significant Tmin decline during the Dry season (*p* = 0.009).

#### Analysis of the relationship between historical observed temperature and precipitation

**Fig. 4:**
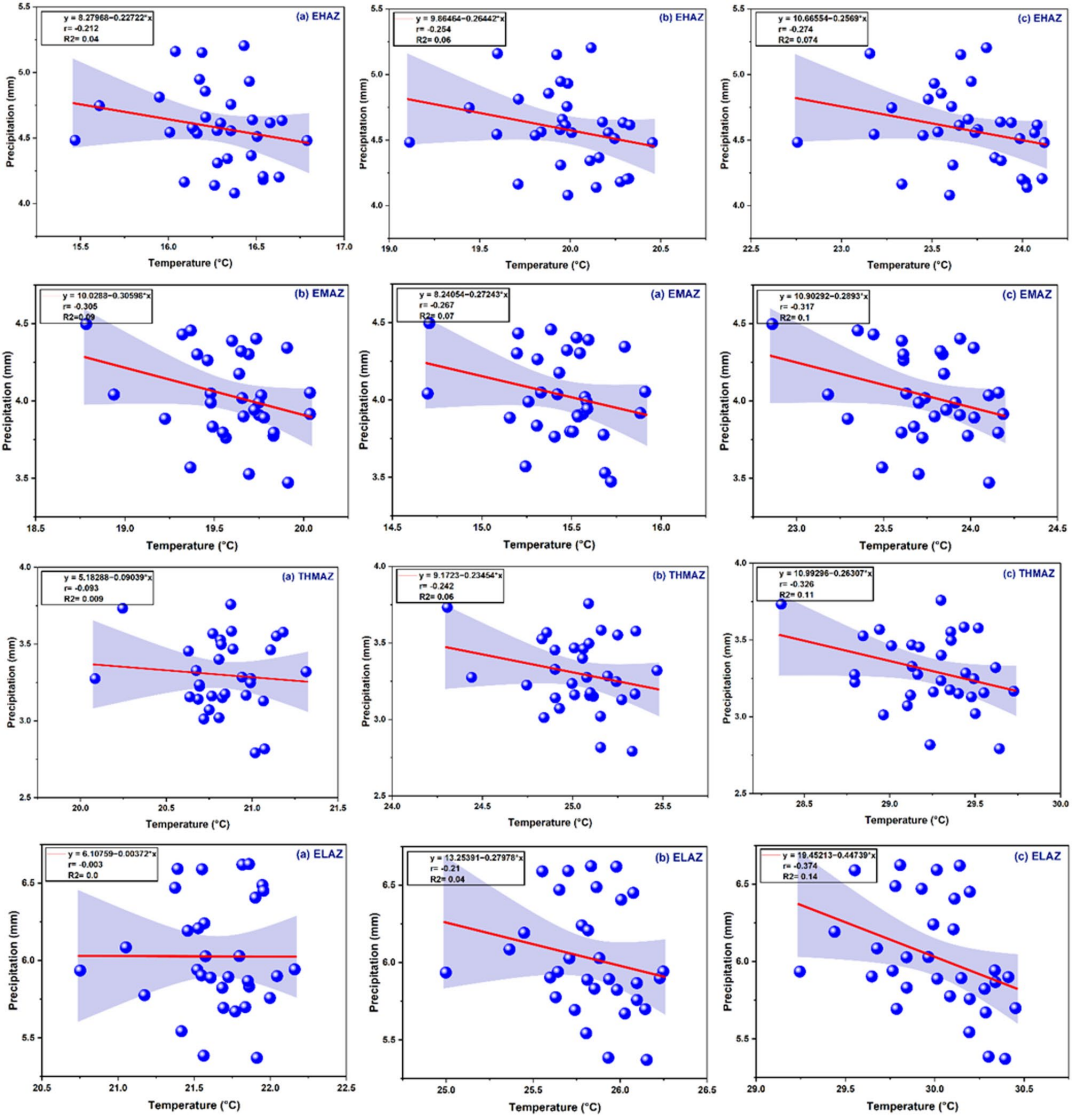

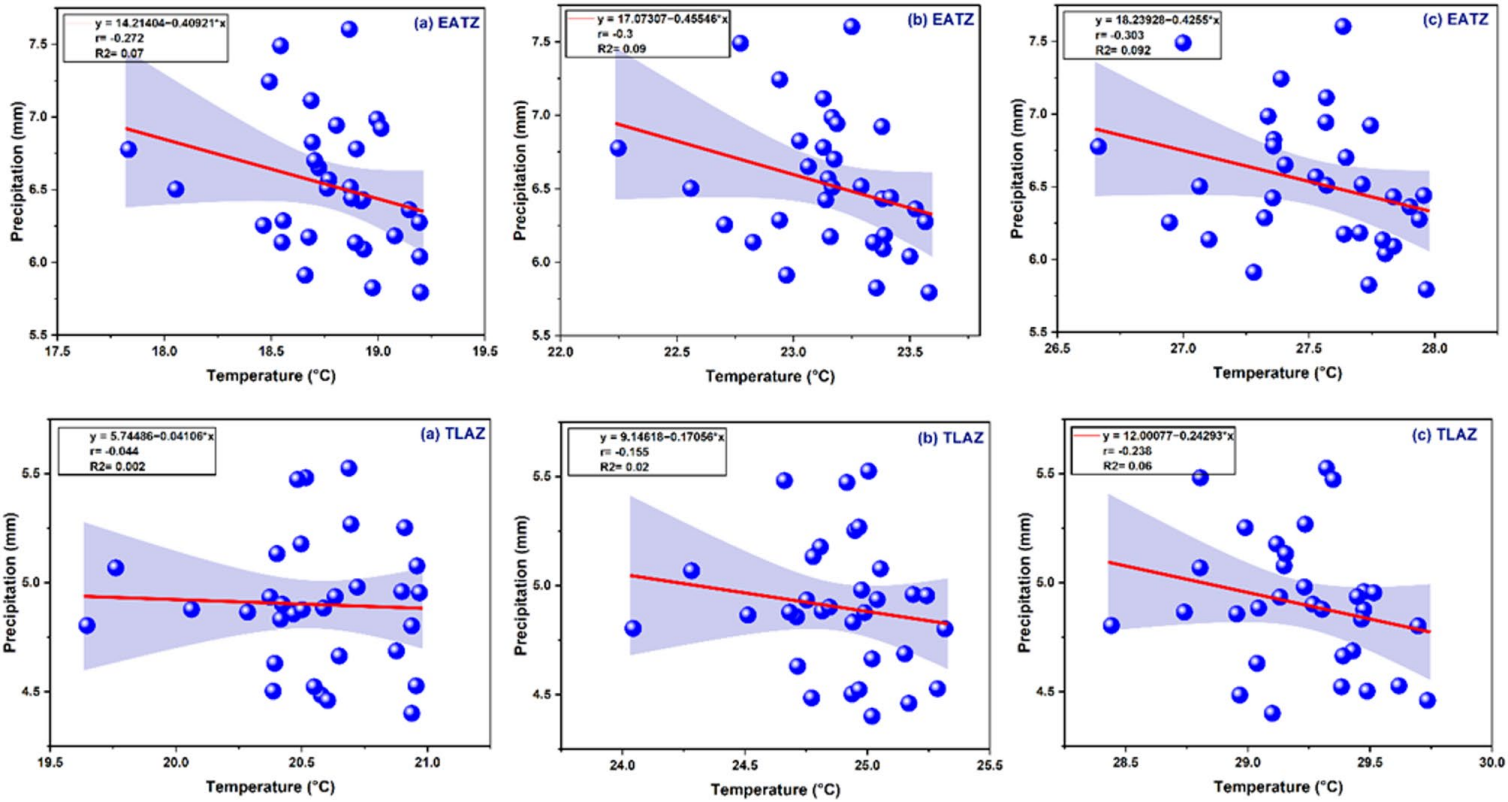
Relationship between observed annual daily average temperature vs. precipitation across AEZs (1983–2014). [(i) Tmin–Precipitation; (ii) Tmean–Precipitation; (iii) Tmax-Precipitation]. EHAZ: Equatorial High-Altitude Zone (EHAZ), EMAZ: Equatorial Mid-Altitude Zone, THMAZ: Tropical High and Mid-Altitude Zone, ELAZ: Equatorial Low Altitude Zone, EATZ: Equatorial Altitude Transition Zone and TLAZ: Tropical Low Altitude Zone.

The correlation analysis revealed inverse statistical associations between temperature parameters (Tmin, Tmean, and Tmax) and precipitation across all AEZs based on annual daily averages (Fig. 4). While these results show a negative relationship where higher temperatures often coincide with lower annual rainfall totals, the magnitude of this inter-annual association varies significantly. Correlation coefficient (r) ranged from − 0.003 (Tmin in ELAZ) to -0.374 (Tmax in ELAZ), with Tmax consistently showing the strongest association.

R^2^ values indicate that temperature explains only a small fraction of the inter annual precipitation variance, ranging from 0.2% in TLAZ to 14% in ELAZ. These low coefficients of determination suggest that temperature is not the primary driver of precipitation variability in the region. Instead, the observed rainfall patterns are likely governed by broader synoptic-scale processes and other meteorological factors. The substantial spread of data points around the regression lines in the scatter plots further confirms that while a weak inverse inter-annual relationship exists, it is exploratory in nature and does not imply a direct causal link.

### CMIP6 model performance assessment

**Fig. 5:**
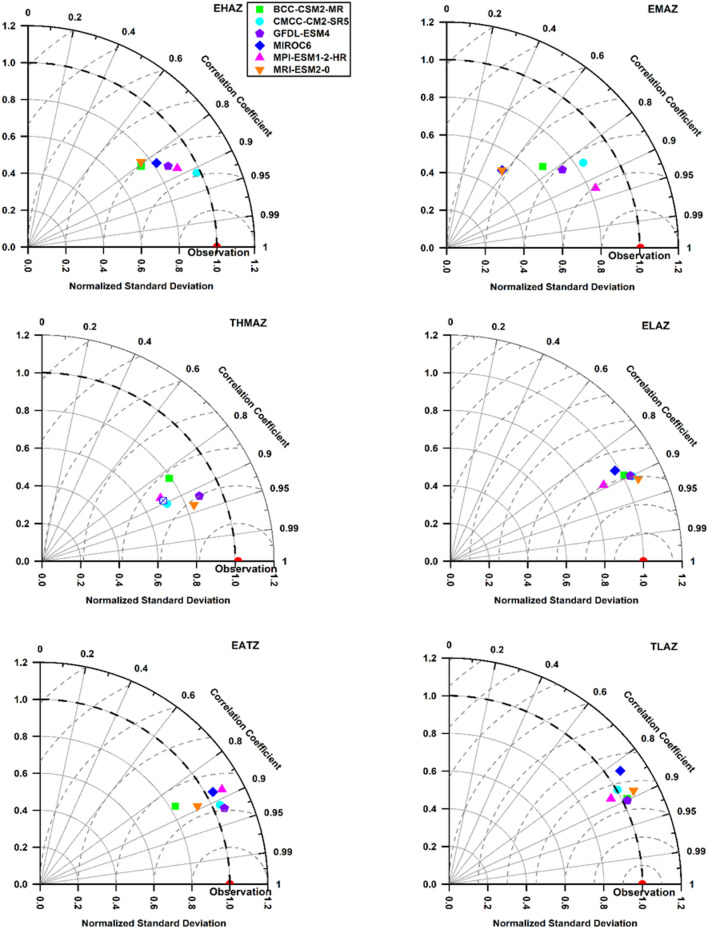
Taylor diagram for precipitation of the individual general circulation models (GCM) at the monthly scale during the validation period of 1983–2014. EHAZ: Equatorial High-Altitude Zone (EHAZ), EMAZ: Equatorial Mid-Altitude Zone, THMAZ: Tropical High and Mid-Altitude Zone, ELAZ: Equatorial Low Altitude Zone, EATZ: Equatorial Altitude Transition Zone and TLAZ: Tropical Low Altitude Zone.

**Fig. 6:**
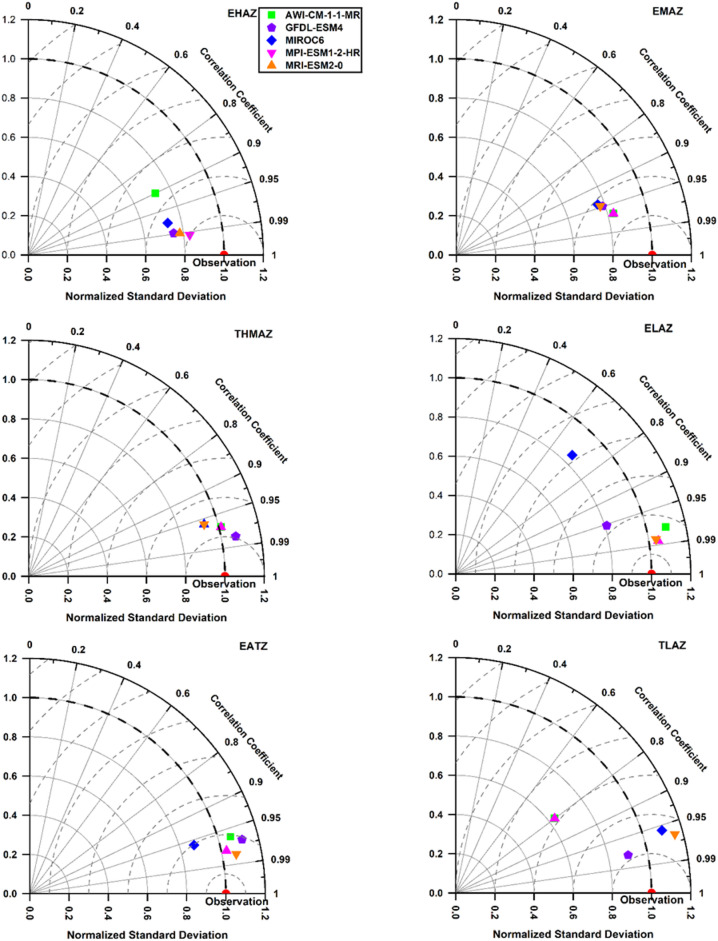
Taylor diagram for Tmax of the individual general circulation models (GCM) : Equatorial High-Altitude Zone (EHAZ), EMAZ: Equatorial Mid-Altitude Zone, THMAZ: Tropical High and Mid-Altitude Zone, ELAZ: Equatorial Low Altitude Zone, EATZ: Equatorial Altitude Transition Zone and TLAZ: Tropical Low Altitude Zone.

**Fig. 7:**
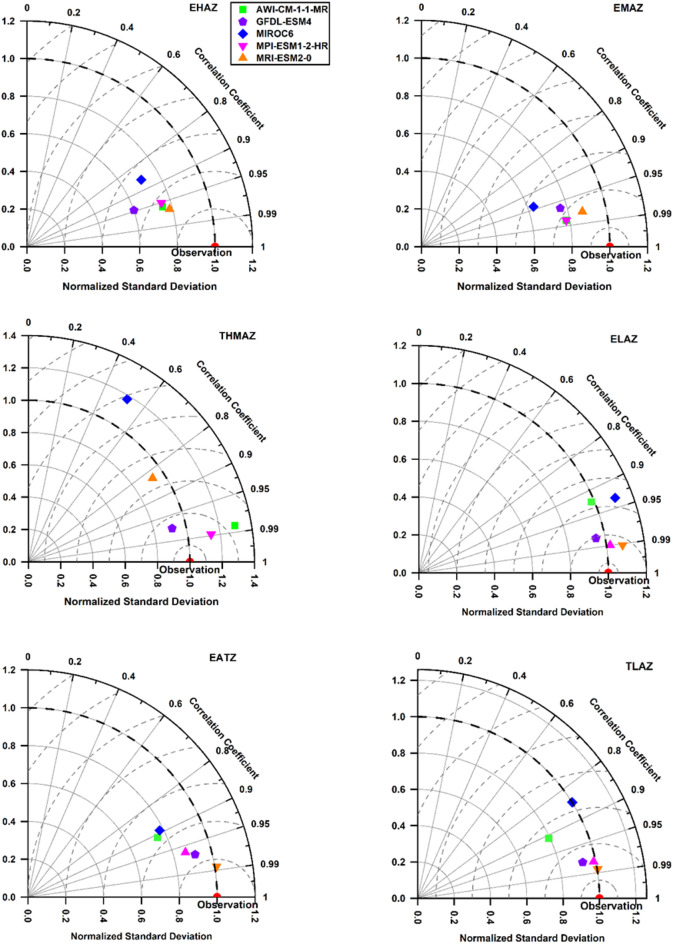
Taylor diagram for Tmin of the individual general circulation models (GCM). Equatorial Mid-Altitude Zone, THMAZ: Tropical High and Mid-Altitude Zone, ELAZ: Equatorial Low Altitude Zone, EATZ: Equatorial Altitude Transition Zone and TLAZ: Tropical Low Altitude Zone.

Taylor diagrams representing the validation performance for precipitation, Tmax and Tmin across the six AEZS are shown in Figs. 5, 6 and 7.

For precipitation, the top-performing models achieved r values ranging from 0.820 to 0.921, reflecting a strong ability to simulate temporal dynamics. CMCC-CM2-SR5 demonstrated superior performance in EHAZ (*r* = 0.911), while MPI-ESM1-2-HR excelled in EMAZ (*r* = 0.912) and THMAZ (*r* = 0.901). In the lowland zones, MRI-ESM2-0 yielded the highest correlations for ELAZ (*r* = 0.912), while BCC-CSM2-MR outperformed in TLAZ (*r* = 0.901). For EATZ, GFDL-ESM4 emerged as the most accurate with correlation of *r* = 0.921.

In reproducing Tmax during validation period, the top-performing models maintained high skill with correlation coefficients (r) generally exceeding 0.90. MRI-ESM2-0 achieved exceptional results in EHAZ (*r* = 0. 958) and performed strongly in EATZ (*r* = 0. 982). MPI-ESM1-2-HR and AWI-CM-1-1-MR showed equivalent high skill in EMAZ (*r* = 0.967, respectively). GFDL-ESM4 was optimal for THMAZ (*r* = 0.982) and achieved the best overall Tmax accuracy in TLAZ (*r* = 0.977). Furthermore, MPI-ESM1-2-HR yielded the highest performance in the ELAZ (*r* = 0.987).

For Tmin, MRI-ESM2-0 consistently achieved the highest correlations across most zones, specifically in EHAZ (*r* = 0.967), ELAZ (*r* = 0.996), EATZ (*r* = 0.987), and TLAZ (*r* = 0.985). MPI-ESM1-2-HR and AWI-CM-1-1-MR proved equivalent high skill in EMAZ (*r* = 0.984, respectively), while MPI-ESM1-2-HR demonstrated high skill in THMAZ (*r* = 0.989). GFDL-ESM4 also showed high performance in ELAZ (*r* = 0.931) and TLAZ (*r* = 0.929).

### Selection of GCMs

**Fig. 8:**
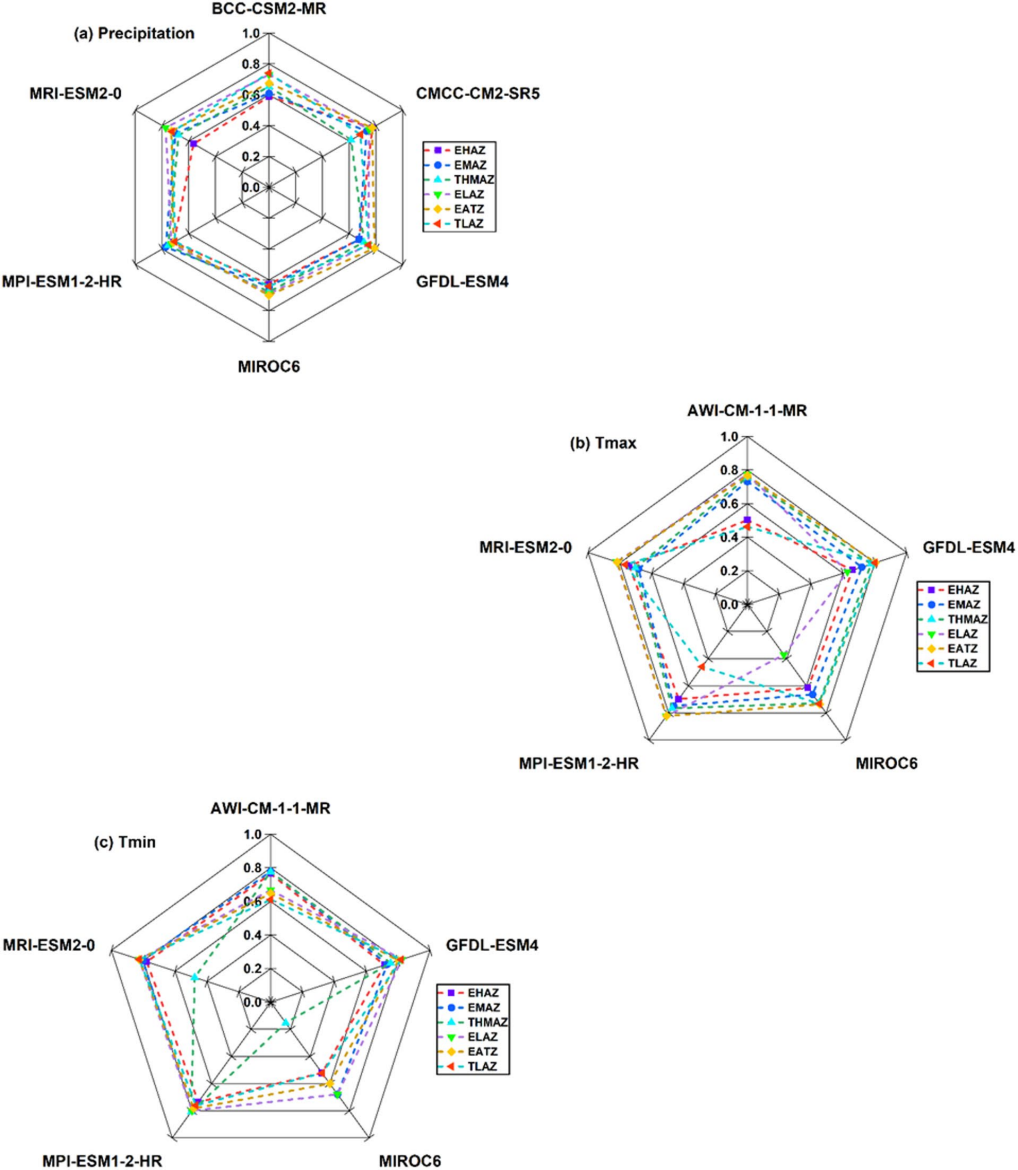
Radar chart for the Taylor skill score (TSS) for individual GCM for (**a**) Precipitation, (**b**) Tmax, and (**c**) Tmin. EHAZ: Equatorial High-Altitude Zone (EHAZ), EMAZ: Equatorial Mid-Altitude Zone, THMAZ: Tropical High and Mid-Altitude Zone, ELAZ: Equatorial Low Altitude Zone, EATZ: Equatorial Altitude Transition Zone and TLAZ: Tropical Low Altitude Zone.

The out-of-sample validation (TSS, RMSE, and PBIAS) for Fold 1 (calibration 1983–1998; validation 1999–2014) are presented in Tables S2-S4, and TSS values are illustrated in Fig. 8. For each variable and AEZ, the three highest- performing models were selected to construct the MME based on their TSS ranking on the Fold 1 validation period. The robustness of this selection is confirmed by the stability index (SI, Tables S5-S9), which measures the fraction of top-3 models that retain their ranking when the validation design is reversed (Fold 2). All 18 AEZ-variable combinations retained at least two of their three top-ranked models across both folds (SI = 0.83 for precipitation, 0.94 for Tmax, 1.00 for Tmin, Tables S5-S9).

For precipitation (Table S2), in the EHAZ, CMCC-CM2-SR5, MPI-ESM1-2-HR, and GFDL-ESM4 were identified as the three top-performing models, with TSS values ranging from 0.675 (GFDL-ESM4) to 0.774 (CMCC-CM2-SR5) and RMSE values between 19.2 and 27.6 mm/month. All three models exhibited a tendency to underestimate mean monthly rainfall, with PBIAS ranging from − 4.4% (CMCC-CM2-SR5) to − 12.5% (GFDL-ESM4). In the EMAZ, MPI-ESM1-2-HR (TSS = 0.774), CMCC-CM2-SR5 (TSS = 0.730), and GFDL-ESM4 (TSS = 0.675) were selected, with RMSE values of 17.6, 15.8, and 24.4 mm/month respectively. In the THMAZ, MPI-ESM1-2-HR (TSS = 0.755) and GFDL-ESM4 (TSS = 0.711) remained top-ranked, while CMCC-CM2-SR5 (TSS = 0.614) was identified as the third ensemble member. In this zone, MPI-ESM1-2-HR and CMCC-CM2-SR5 showed slight overestimation of rainfall (PBIAS of + 2.2% and + 13.4% respectively), whereas GFDL-ESM4 continued to underestimate (PBIAS = − 3.9%). In the ELAZ, the top-performing models were MRI-ESM2-0 (TSS = 0.774), BCC-CSM2-MR (TSS = 0.749), and GFDL-ESM4 (TSS = 0.744), with RMSE values tightly contained between 18.8 and 22.4 mm/month. In the EATZ, GFDL-ESM4, CMCC-CM2-SR5, and MRI-ESM2-0 captured rainfall patterns with high precision; GFDL-ESM4 achieved the lowest RMSE (12.1 mm/month) and a minimal PBIAS of + 1.4%. In the TLAZ, BCC-CSM2-MR, MPI-ESM1-2-HR and MRI-ESM2-0 were selected; these models maintained the highest TSS and lowest RMSE values in the zone, with PBIAS ranging from − 8.90% (BCC-CSM2-MR) to + 3.4% (MPI-ESM1-2-HR).

For Tmax (Table S3) in the EHAZ, MRI-ESM2-0 (TSS = 0.872), MPI-ESM1-2-HR (TSS = 0.841), and GFDL-ESM4 (TSS = 0.825) were the top-performing models; MRI-ESM2-0 demonstrated exceptional accuracy with a minimal RMSE of 0.22 °C and a PBIAS of + 0.42%. In the EATZ, MRI-ESM2-0 (TSS = 0.926) achieved the highest precision with an RMSE of 0.44 °C, followed closely by MPI-ESM1-2-HR (TSS = 0.916) and GFDL-ESM4 (TSS = 0.901). In the EMAZ, THMAZ, and ELAZ the ensemble comprised MPI-ESM1-2-HR, AWI-CM-1-1-MR, and GFDL-ESM4. In the EMAZ, MPI-ESM1-2-HR ranked first (TSS = 0.898), while GFDL-ESM4 was ranked third (TSS = 0.856). In the THMAZ, GFDL-ESM4 ranked first (TSS = 0.926), supported by a low PBIAS of + 0.72%. In the ELAZ, MPI-ESM1-2-HR achieved high TSS (0.945) but underestimate the Tmax (PBIAS = -1.07). In the TLAZ, GFDL-ESM4 (TSS = 0.916, RMSE = 0.99 °C, PBIAS = − 1.36%), MRI-ESM2-0 (TSS = 0.896), and MIROC6 (TSS = 0.870) were selected.

For Tmin (Table S4), MRI-ESM2-0, AWI-CM-1-1-MR, and MPI-ESM1-2-HR were selected for both the EHAZ and EMAZ. In the EMAZ, these three models demonstrated high precision with RMSE values as low as 0.33 °C (MPI-ESM1-2-HR) and PBIAS ranging from + 1.49% to + 2.1%. In the EHAZ, MRI-ESM2-0 (TSS = 0.898, RMSE = 0.91 °C) maintained reliable physical precision despite the challenging nocturnal thermal regime of high-altitude zones. For the THMAZ, MPI-ESM1-2-HR, AWI-CM-1-1-MR, and GFDL-ESM4 were identified as the most skillful models; MPI-ESM1-2-HR ranked first (TSS = 0.949) with an exceptional RMSE of 0.17 °C and a minimal PBIAS of + 0.63%. In the ELAZ, EATZ, and TLAZ, the TSS consistently favored MRI-ESM2-0, GFDL-ESM4, and MPI-ESM1-2-HR, with TSS values exceeding 0.88 across all three zones. In the ELAZ, MRI-ESM2-0 achieved the highest absolute accuracy with an RMSE of 0.12 °C and a negligible PBIAS of − 0.18%. Across the EATZ and TLAZ, the selected models maintained RMSE values predominantly below 1.4 °C and PBIAS within ± 1.6%, confirming robust representation of minimum temperature profiles in the lowland and transitional zones.

### Projection of climatic variables

#### Projected precipitation

**Fig. 9:**
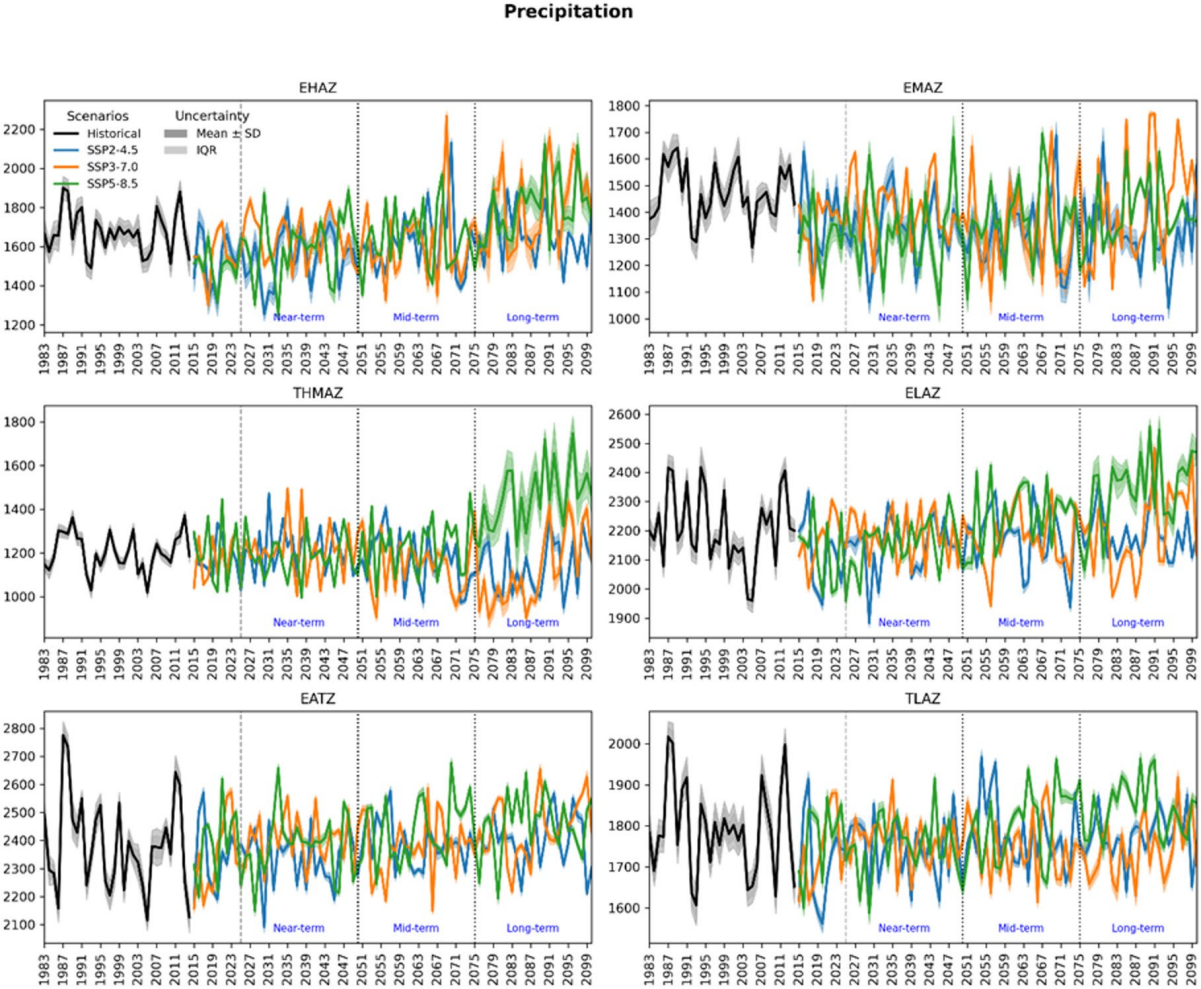
Projected annual daily average precipitation (ref. period 1983 to 2100), ensemble model. EHAZ: Equatorial High-Altitude Zone (EHAZ), EMAZ: Equatorial Mid-Altitude Zone, THMAZ: Tropical High and Mid-Altitude Zone, ELAZ: Equatorial Low Altitude Zone, EATZ: Equatorial Altitude Transition Zone and TLAZ: Tropical Low Altitude Zone.

Table 4 summarizes the projected precipitation changes across the three 25-year periods under SSP2-4.5, SSP3-7.0, and SSP5-8.5, while Fig. 9 provides trajectory of annual rainfall from 1983 to 2100. Precipitation projections vary substantially across AEZs and scenarios. The results revealed a declining precipitation trend in the near- and mid-term periods, followed by increases in the long term. SSP5-8.5 may lead to the most significant increase in the far future. However, differences remain evident across the AEZs. Regardless of the magnitude of future rainfall variability, the EATZ and ELAZ consistently maintain the highest annual precipitation across all periods, whereas the THMAZ remains the lowest, except in the long term under SSP5-8.5, when it exceeds the EMAZ.

**Table 4:** Relative change in annual precipitation (mm) in the near-term (2026–2050), mid-term (2051–2075), and long-term (2076–2100) relative to the reference period (1983–2014) under different climate change scenarios across South-Kivu’s agroecological zones.

Scenario	AEZs	Observed (mm)	2026–2050				2051–2075				2076–2100			
			**Mean** **(%)**	**Min (%)**	**Max (%)**	**SD (%)**	**Mean (%)**	**Min (%)**	**Max (%)**	**SD (%)**	**Mean (%)**	Min (%)	Max (%)	SD (%)
SSP2-4.5	EHAZ	1672	-7.8	-11.62	-4.84	3.47	-3	-4.49	-1.86	1.35	-0.6	-1.65	0.1	0.93
	EMAZ	1472	-9.4	-11.94	-5.83	3.18	-10.1	-12.83	-6.26	3.42	-9.8	-12.44	-6.08	3.31
	THMAZ	1204	0.9	0.2	1.6	0.7	-3.5	-4.97	-2.17	1.41	-5.7	-8.09	-3.53	2.29
	ELAZ	2200	-2.5	-3.38	-1.97	0.77	-0.9	-1.6	-0.55	0.61	0.6	-0.1	0.95	0.61
	EATZ	2383	-2.1	-2.8	-1.75	0.61	0.3	-0.4	0.65	0.58	0.7	0	1.05	0.61
	TLAZ	1789	-2.6	-3.51	-1.61	0.95	-0.5	-1.2	0.2	0.7	1.8	1.1	2.5	0.7
SSP3-7.0	EHAZ	1672	0.1	-0.95	0.8	0.93	-4.1	-6.11	-2.54	1.83	8.9	4.54	12.28	3.96
	EMAZ	1472	-1.8	-2.15	-1.1	0.61	-9.2	-11.69	-5.7	3.12	-0.8	-1.15	-0.1	0.61
	THMAZ	1204	0.8	0.1	1.5	0.7	-4.9	-6.96	-3.04	1.97	-7.1	-10.08	-4.4	2.85
	ELAZ	2200	-0.5	-1.2	-0.15	0.61	-1.1	-1.8	-0.75	0.61	1.2	0.5	1.55	0.61
	EATZ	2383	0.7	0	1.05	0.61	-0.3	-1	0.05	0.61	2.6	1.9	2.95	0.61
	TLAZ	1789	-1.6	-2.3	-0.9	0.7	-2.3	-3.1	-1.43	0.84	2.9	1.89	4	1.06
SSP5-8.5	EHAZ	1672	-4.6	-6.86	-2.85	2.05	-1.9	-2.97	-1.18	0.94	9.5	4.85	13.11	4.23
	EMAZ	1472	-11.3	-14.35	-7.01	3.82	-7.4	-9.4	-4.59	2.51	-3.2	-4.07	-1.98	1.09
	THMAZ	1204	-1.9	-2.7	-1.18	0.76	2.2	1.28	3.04	0.88	21.2	12.3	28.26	8.14
	ELAZ	2200	-1.5	-2.2	-1.15	0.61	2.3	1.49	2.76	0.7	6.9	4.49	8.69	2.17
	EATZ	2383	-0.3	-1	0.05	0.61	1.9	1.2	2.25	0.61	2.6	1.9	2.95	0.61
	TLAZ	1789	-2.5	-3.38	-1.55	0.92	0.9	0.2	1.6	0.7	2.6	1.69	3.59	0.95

EHAZ: Equatorial High-Altitude Zone (EHAZ), EMAZ: Equatorial Mid-Altitude Zone, THMAZ: Tropical High and Mid-Altitude Zone, ELAZ: Equatorial Low Altitude Zone, EATZ: Equatorial Altitude Transition Zone and TLAZ: Tropical Low Altitude Zone. Relative values in brackets [Min ; Max] indicate the spread between the three selected models.

In the near-term, annual precipitation is projected to decline by -0.1% to -9.7% across most AEZs. The EMAZ shows a consistent decrease (-3.9% to -9.7%) across all scenarios, followed by the EHAZ (-4.6% ± 2.05 to -7.8% ± 3.47). ELAZ and TLAZ will likely experience a moderate decline within a range from − 0.5% ± 0.61 to -2.6 ± 0.95%. Conversely, the THMAZ shows a slight increase under SSP3-7.0 and SSP2-4.5 (+ 0.8% ± 0.7 to + 0.9% ± 0.7, respectively) but decreased under SSP5-8.5 (-1.9% ± 0.76). The EATZ is predicted to decline by -2.1% ± 0.61 under SSP2-4.5, increase under SSP3-7.0 (+ 0.7% ± 0.61) and stabilize under SSP5-8.5.

In the mid-term, the EMAZ continues to experience the largest projected decrease (-7.4% ± 2.51 to -10.1% ± 3.42). Under SSP2-4.5 and SSP3-7.0, the EATZ will stabilize within a range of -0.3% ± 0.61 to 0.3% ± 0.58, whereas the THMAZ, EHAZ, TLAZ, and ELAZ will decrease by -4.9% ± 1.97, − 4.1% ± 1.83, − 2.3% ± 0.84, and − 1.1% ± 0.61, respectively. Under high-emission SSP5-8.5 scenario, a divergence occurs: while the EMAZ and EHAZ continue to decline, the lowland zones (ELAZ and TLAZ) and EATZ projected to increase by + 0.9% ± 0.7 to 2.3% ± 0.7.

In the long term, most AEZs exhibit increased precipitation, particularly under SSP5-8.5. Notably, while THMAZ decreases under lower-emission scenarios, it shows a dramatic projected increase under SSP5-8.5 (+ 21% ± 8.14). In this far-future period under SSP5-8.5, the magnitude of increase follows the order: THMAZ (+ 21% ± 8.14) > EHAZ (+ 9.5% ± 4.23) > ELAZ (+ 6.9% ± 2.17) > TLAZ (+ 2.0% ± 0.61) > EATZ (+ 2.6% ± 0.95). Despite these increases, the EMAZ remains an outlier with persistent declines (0.8% ± 0.61 to 9.8% ± 3.31).

#### Projected temperatures

**Fig. 10:**
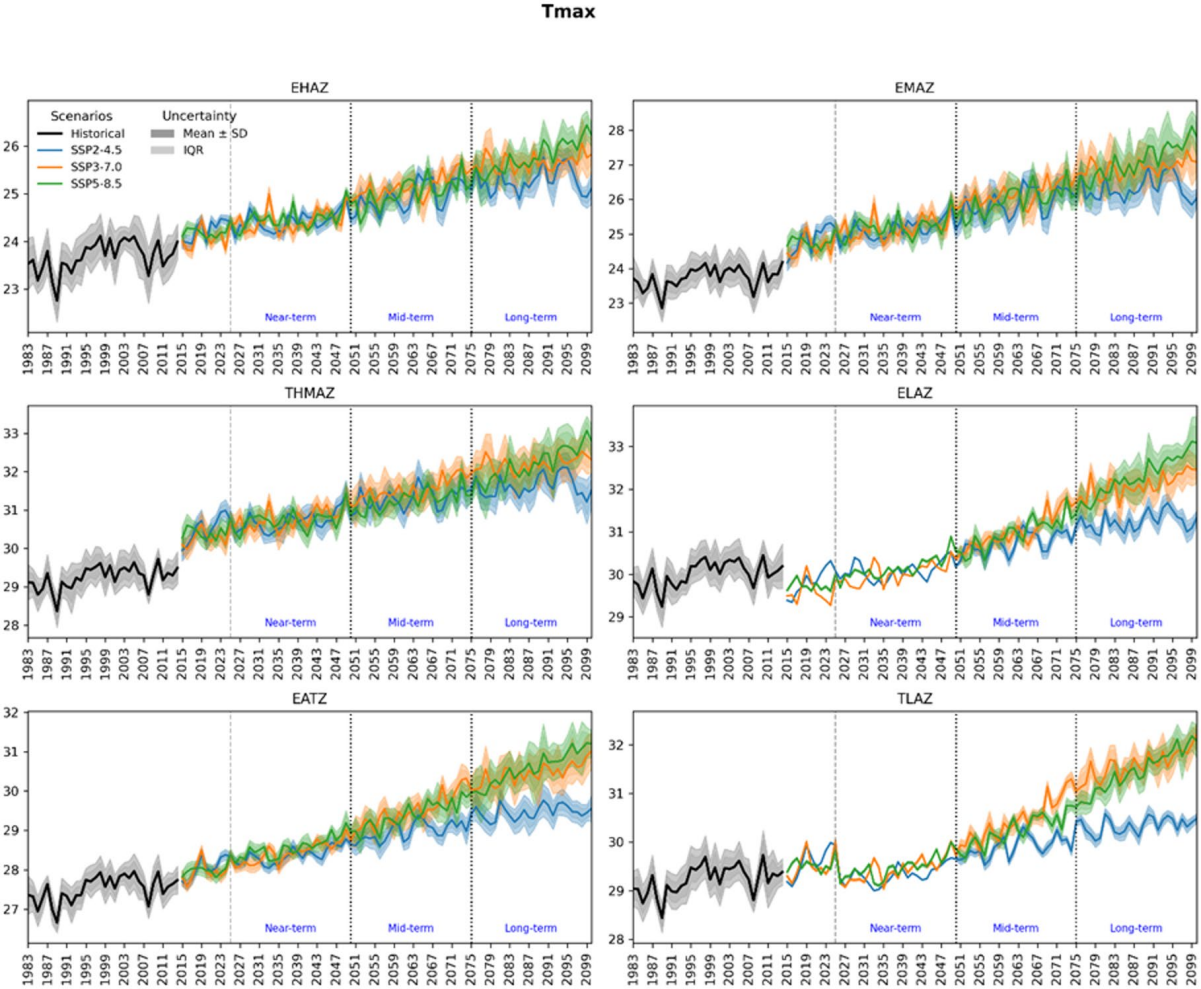
Projected annual daily average surface air Tmax (ref. period 1983 to 2100), ensemble model. EHAZ: Equatorial High-Altitude Zone (EHAZ), EMAZ: Equatorial Mid-Altitude Zone, THMAZ: Tropical High and Mid-Altitude Zone, ELAZ: Equatorial Low Altitude Zone, EATZ: Equatorial Altitude Transition Zone and TLAZ: Tropical Low Altitude Zone.

**Fig. 11:**
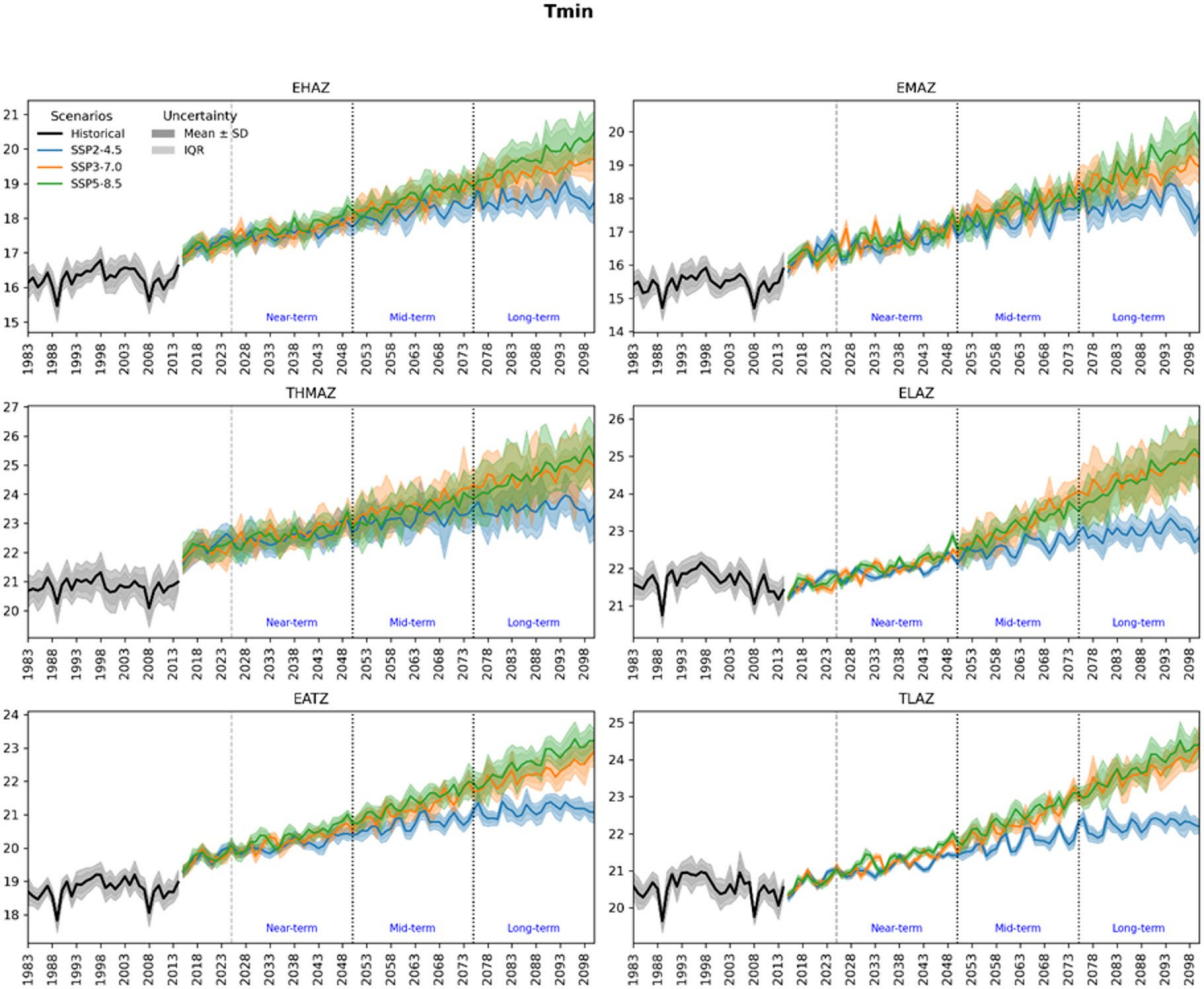
Projected annual daily average surface air Tmin (ref. period 1983 to 2100), ensemble model. EHAZ: Equatorial High-Altitude Zone (EHAZ), EMAZ: Equatorial Mid-Altitude Zone, THMAZ: Tropical High and Mid-Altitude Zone, ELAZ: Equatorial Low Altitude Zone, EATZ: Equatorial Altitude Transition Zone and TLAZ: Tropical Low Altitude Zone.

Substantial and continuous warming is projected across all AEZs and scenarios (Tables 5 and 6; Figs. 10 and 11). Warming is directly proportional to emission intensity, peaking in the long-term under SSP5-8.5. Both Tmax and Tmin are projected to rise by up to 3.89 °C by the late century, though nighttime temperatures exhibit greater overall increases than daytime temperatures.

**Table 5:** Absolute change in annual Tmax in the near-term (2026–2050), mid-term (2051–2075), and long-term (2076–2100) relative to the reference period (1983–2014) under different climate change scenarios across South-Kivu’s agroecological zones.

Scenario	AEZs	Observed (°C)	2026–2050				2051–2075				2076–2100			
			**Mean** **(°C)**	**Min (°C)**	**Max (°C)**	**SD (°C)**	**Mean (°C)**	**Min (°C)**	**Max (°C)**	**SD (°C)**	**Mean (°C)**	Min (°C)	Max (°C)	SD (°C)
SSP2-4.5	EHAZ	23.69	0.74	0.61	0.86	0.13	1.28	1.06	1.47	0.21	1.59	1.32	1.83	0.26
	EMAZ	23.75	1.39	1.15	1.61	0.23	2.17	1.8	2.52	0.36	2.58	2.14	2.99	0.43
	THMAZ	29.24	1.47	1.22	1.71	0.25	2.08	1.73	2.41	0.34	2.38	1.98	2.76	0.39
	ELAZ	30.01	0.07	0.06	0.08	0.01	0.75	0.62	0.87	0.13	1.24	1.03	1.44	0.21
	EATZ	27.52	0.85	0.71	0.97	0.13	1.47	1.22	1.69	0.24	1.95	1.62	2.24	0.31
	TLAZ	29.23	0.1	0.09	0.11	0.01	0.7	0.61	0.78	0.09	1.11	0.97	1.23	0.13
SSP3-7.0	EHAZ	23.69	0.76	0.63	0.87	0.12	1.49	1.24	1.71	0.24	1.98	1.64	2.28	0.32
	EMAZ	23.75	1.46	1.21	1.69	0.24	2.43	2.02	2.82	0.4	3.17	2.63	3.68	0.53
	THMAZ	29.24	1.54	1.28	1.79	0.26	2.32	1.93	2.69	0.38	2.97	2.47	3.45	0.49
	ELAZ	30.01	0.02	0.02	0.02	0	1.04	0.86	1.21	0.18	2.09	1.73	2.42	0.35
	EATZ	27.52	0.93	0.77	1.07	0.15	2.02	1.68	2.32	0.32	2.94	2.44	3.38	0.47
	TLAZ	29.23	0.22	0.19	0.25	0.03	1.25	1.09	1.39	0.15	2.38	2.07	2.64	0.29
SSP5-8.5	EHAZ	23.69	0.79	0.66	0.9	0.12	1.35	1.12	1.55	0.22	2.1	1.74	2.42	0.34
	EMAZ	23.75	1.46	1.21	1.69	0.24	2.24	1.86	2.6	0.37	3.38	2.81	3.92	0.56
	THMAZ	29.24	1.49	1.24	1.73	0.25	2.02	1.68	2.34	0.33	2.97	2.47	3.45	0.49
	ELAZ	30.01	0.15	0.12	0.17	0.03	0.97	0.81	1.13	0.16	2.31	1.92	2.68	0.38
	EATZ	27.52	1.06	0.88	1.22	0.17	1.9	1.58	2.18	0.3	3.08	2.56	3.54	0.49
	TLAZ	29.23	0.25	0.22	0.27	0.03	1.03	0.9	1.14	0.12	2.25	1.96	2.5	0.27

EHAZ: Equatorial High-Altitude Zone (EHAZ), EMAZ: Equatorial Mid-Altitude Zone, THMAZ: Tropical High and Mid-Altitude Zone, ELAZ: Equatorial Low Altitude Zone, EATZ: Equatorial Altitude Transition Zone and TLAZ: Tropical Low Altitude Zone.

**Table 6:** Absolute change in annual Tmin in the near-term (2026–2050), mid-term (2051–2075), and long-term (2076–2100) relative to the reference period (1983–2014) under different climate change scenarios across South-Kivu’s agroecological zones.

Scenario	AEZs	Observed (°C)	2026–2050				2051–2075				2076–2100			
			**Mean** **(°C)**	**Min (°C)**	**Max (°C)**	**SD (°C)**	**Mean (°C)**	**Min (°C)**	**Max (°C)**	**SD (°C)**	**Mean (°C)**	**Min (°C)**	Max (°C)	SD (°C)
SSP2-4.5	EHAZ	16.28	1.29	1.05	1.5	0.23	1.91	1.56	2.22	0.33	2.29	1.87	2.66	0.4
	EMAZ	15.44	1.25	1.04	1.44	0.2	2	1.66	2.3	0.32	2.41	2	2.77	0.39
	THMAZ	20.83	1.72	1.43	2.23	0.44	2.34	1.94	3.04	0.61	2.68	2.22	3.49	0.7
	ELAZ	21.65	0.31	0.26	0.4	0.08	0.95	0.79	1.23	0.24	1.33	1.1	1.73	0.35
	EATZ	18.78	1.34	1.17	1.48	0.16	1.95	1.7	2.16	0.23	2.36	2.05	2.62	0.29
	TLAZ	20.55	0.58	0.5	0.65	0.08	1.23	1.07	1.37	0.15	1.67	1.45	1.86	0.21
SSP3-7.0	EHAZ	16.28	1.36	1.11	1.58	0.24	2.28	1.87	2.64	0.39	3.04	2.49	3.53	0.52
	EMAZ	15.44	1.37	1.14	1.57	0.22	2.35	1.95	2.7	0.38	3.23	2.68	3.72	0.52
	THMAZ	20.83	1.86	1.54	2.42	0.49	2.85	2.37	3.7	0.74	3.83	3.18	4.98	1
	ELAZ	21.65	0.42	0.35	0.54	0.1	1.58	1.31	2.06	0.42	2.87	2.38	3.73	0.75
	EATZ	18.78	1.47	1.28	1.63	0.18	2.45	2.13	2.72	0.3	3.46	3.01	3.84	0.42
	TLAZ	20.55	0.71	0.62	0.79	0.09	1.79	1.56	1.98	0.21	3.09	2.69	3.43	0.37
SSP5-8.5	EHAZ	16.28	1.47	1.2	1.71	0.26	2.33	1.91	2.7	0.4	3.48	2.85	4.04	0.6
	EMAZ	15.44	1.35	1.12	1.55	0.22	2.28	1.89	2.62	0.37	3.58	2.97	4.12	0.58
	THMAZ	20.83	1.79	1.49	2.32	0.46	2.65	2.2	3.44	0.69	3.89	3.23	5.06	1.02
	ELAZ	21.65	0.49	0.41	0.63	0.12	1.49	1.24	1.93	0.38	2.79	2.32	3.62	0.72
	EATZ	18.78	1.59	1.38	1.77	0.2	2.61	2.27	2.9	0.32	3.83	3.33	4.25	0.47
	TLAZ	20.55	0.83	0.72	0.92	0.1	1.92	1.67	2.13	0.23	3.23	2.81	3.59	0.39

EHAZ: Equatorial High-Altitude Zone (EHAZ), EMAZ: Equatorial Mid-Altitude Zone, THMAZ: Tropical High and Mid-Altitude Zone, ELAZ: Equatorial Low Altitude Zone, EATZ: Equatorial Altitude Transition Zone and TLAZ: Tropical Low Altitude Zone.

Daytime temperatures are projected to increase from 0.07 °C ± 0.01 to 1.54 °C ± 0.26 by 2050s and from 1.11 °C ± 0.13 to 3.38 °C ± 0.56 in the 2100s. Under SSP2-4.5, the projected absolute temperature increases in the near- to long-term range from 1.39 °C ± 0.23 to 2.58 °C ± 0.43, from 1.47 °C ± 0.25 to 2.38 °C ± 0.39, from 0.85 °C ± 0.13 to 1.95 °C ± 0.31, from 0.74 °C ± 0.13 to 1.59 °C ± 0.26, from 0.07 °C ± 0.01 to 1.24 °C ± 0.21 and from 0.1 °C ± 0.01 to 1.11 °C ± 0.13 in the EMAZ, THMAZ, EATZ, EHAZ, ELAZ, and TLAZ, respectively. Under high-emission SSP5 scenario, Tmax increases range from 1.46 ± 0.24 to 3.38 °C ± 0.56 in the EMAZ, 1.49 °C ± 0.25 to 2.97 °C ± 0.49 in the THMAZ, 1.06 °C ± 0.17 to 3.08 °C ± 0.49 in the EATZ, 0.79 °C ± 0.12 to 2.1 °C ± 0.34 in the EHAZ, 0.25 °C ± 0.03 to 2.25 °C ± 0.27 in the TLAZ and 0.15 ± 0.03 to 2.31 °C ± 0.38 in the ELAZ from the near- to late-century period.

Nighttime temperatures show more pronounced warming, with increase from 0.3 °C ± 0.08 to 1.86 °C ± 0.49 by mid-century and up to 3.89 °C ± 1.02 by late-century relative to the baseline. Under SSP5-8.5, the temperature increases range from 1.79 °C ± 0.46 to 3.89 °C ± 1.02, from 1.59 °C ± 0.2 to 3.83 °C ± 0.47, from 1.47 °C ± 0.26 to 3.48 °C ± 0.6, from 1.35 °C ± 0.22 to 3.58 °C ± 0.58, from 0.83 °C ± 0.1 to 3.23 °C ± 0.39, and from 0.49 °C ± 0.12 to 2.79 °C ± 0.72 in the THMAZ, EATZ, EHAZ, EMAZ, TLAZ, and ELAZ, respectively, from the near to far-future periods. Under SSP2-4.5, the temperature increases from 1.72 °C ± 0.44 to 2.68 °C ± 0.7, from 1.34 °C ± 0.16 to 2.36 °C ± 0.29, from 1.25 °C ± 0.22 to 2.41 °C ± 0.39, from 1.29 °C ± 0.23 to 2.29 °C ± 0.4, from 0.58 °C ± 0.08 to 1.67 °C ± 0.21, and from 0.31 °C ± 0.08 to 1.33 °C ± 0.35 in THMAZ, EATZ, EMAZ, EHAZ, TLAZ, and ELAZ, respectively.

Inter-AEZ comparisons reveal that THMAZ will consistently experience the highest temperatures. Daytime peaks in the THMAZ are projected to reach 30.72 °C ± 0.25 to 32.21 °C ± 0.49, while nighttime temperatures may reach 24.73 °C ± 1.02 under the most unfavorable SSP5-8.5 conditions.

#### Seasonal changes in precipitation

**Fig. 12:**
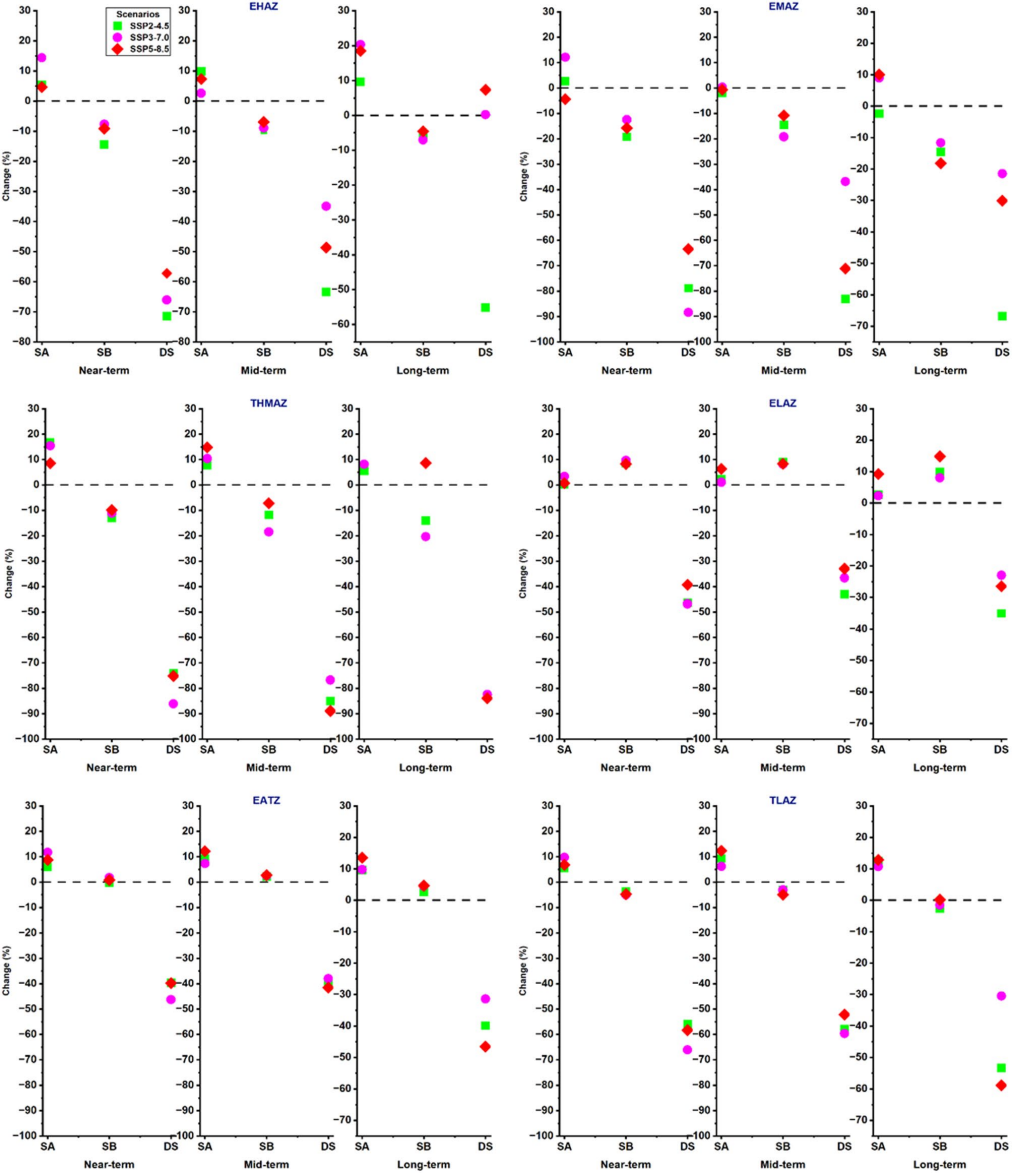
Projected seasonal changes in percentage of precipitation in six AEZs at different time period scales in Near-term, Mid-term, and Long-term under SSP2-4.5, SSP3-7.0 and SSP5-8.5 from the Model Ensemble relative to the reference period of 1983–2014. SA: Season A; SB: Season B; and DS: Dry season.

The seasonal precipitation projections across AEZs are presented in Fig. 12; Table 7. Model results indicate a clear redistribution of seasonal rainfall, with shifts intensifying throughout the century under the high-emission SSP5-8.5 scenario. Precipitation is projected to increasingly concentrate during the long rainy Season A, while Season B declines in most zones. Sign agreement reaches 100% in 52 of 54 AEZ-season-scenario combinations, with two exceptions: EMAZ season A under SSP2-4.5 (67% sign agreement; -0.54% ± 0.1) and THMAZ Season B under SSP5-8.5 (67% sign agreement; -2.8% ± 0.7). ELAZ and EATZ are the only zones where both rainy seasons are projected to increase under all scenarios.

**Table 7:** Projected seasonal precipitation deltas (%) across AEZs and SSP scenarios (2026–2100).

AEZs	Seasons	SSP2-4.5				SSP3-7.0				SSP5-8.5			
		**Mean delta (%)**	**SD**	**95% CI**	**Sign**	**Mean delta (%)**	**SD**	**95% CI**	**Sign**	**Mean delta (%)**	**SD**	**95% CI**	Sign
EHAZ	Season A	8.29	2.1	[3.07, 13.51]	100	10.18	2.6	[7.8, 12.5]	100	12.45	3.2	[9.6, 15.3]	100
	Season B	-9.89	2.5	[-12.1, -7.6]	100	-7.9	2	[-9.7, -6.1]	100	-6.88	1.7	[-8.4, -5.3]	100
	Dry season	-63.32	15.8	[-77.5, -49.1]	100	-33.58	8.4	[-41.1, -26.1]	100	-32.84	8.2	[-40.2, -25.5]	100
EMAZ	Season A	-0.54	0.1	[-0.79, -0.29]	67	1.7	0.4	[1.3, 2.1]	100	7.14	1.8	[5.5, 8.8]	100
	Season B	-16.09	4	[-19.7, -12.5]	100	-14.46	3.6	[-17.7, -11.2]	100	-14.93	3.7	[-18.3, -11.6]	100
	Dry season	-76.24	19.1	[-93.3, -59.1]	100	-48.91	12.2	[-59.9, -37.9]	100	-54.87	13.7	[-67.2, -42.5]	100
THMAZ	Season A	9.97	2.5	[7.7, 12.2]	100	11.32	2.8	[8.7, 13.9]	100	20.23	5.1	[15.6, 24.9]	100
	Season B	-12.97	3.2	[-15.9, -10.1]	100	-16.64	4.2	[-20.4, -12.9]	100	-2.8	0.7	[-3.4, -2.2]	67
	Dry season	-80.8	20.2	[-98.9, -62.6]	100	-81.77	20.4	[-99.0, -63.4]	100	-82.65	20.7	[-99.0, -64.1]	100
ELAZ	Season A	1.78	0.4	[1.4, 2.2]	100	2.24	0.6	[1.7, 2.8]	100	5.43	1.4	[4.2, 6.7]	100
	Season B	9.13	2.3	[7.0, 11.2]	100	8.63	2.2	[6.6, 10.6]	100	10.47	2.6	[8.1, 12.8]	100
	Dry season	-41.46	10.4	[-50.8, -32.1]	100	-35.46	8.9	[-43.4, -27.5]	100	-32.9	8.2	[-40.3, -25.5]	100
EATZ	Season A	8.3	2.1	[6.4, 10.2]	100	9.61	2.4	[7.4, 11.8]	100	11.51	2.9	[8.9, 14.1]	100
	Season B	1.54	0.4	[1.2, 1.9]	100	2.93	0.7	[2.3, 3.6]	100	2.72	0.7	[2.1, 3.3]	100
	Dry season	-40.01	10	[-49.0, -31.0]	100	-38.55	9.6	[-47.2, -29.9]	100	-42.61	10.7	[-52.2, -33.0]	100
TLAZ	Season A	9.12	2.3	[7.0, 11.2]	100	8.9	2.2	[6.9, 10.9]	100	10.65	2.7	[8.2, 13.1]	100
	Season B	-3.12	0.8	[-3.8, -2.4]	100	-3.22	0.8	[-3.9, -2.5]	100	-3.17	0.8	[-3.9, -2.5]	100
	Dry season	-55.77	13.9	[-68.3, -43.2]	100	-52.04	13	[-63.8, -40.3]	100	-56.42	14.1	[-69.1, -43.7]	100

In EHAZ, Season A precipitation is projected to increase by + 8.29% ± 2.1 (SSP2-4.5) to 12.45% ± 3.2 (SSP5-8.5), while Season B declines by -6.88% ± 1.7 to 9.89% ± 2.5. THMAZ substantial Season A gains of up to + 20.23% ± 5.1 under SSP5-8.5, partially offset by Season B losses of up -16.64% ± 4.2 under SSP3-7.0; notably, the THMAZ Season B signal under SSP5-8.5 carries only 67% sign agreement, reflecting near-neutral ensemble behavior at high emissions in this zone. TLAZ follows similar Season A intensification pattern (up to + 10.65% ± 2.7 under SSP5-8.5) alongside a small but consistent Season B decline of -3.12% to -3.22% across all scenarios. In the EMAZ, Season A displays a slight near-neutral change under SSP2-4.5 (-0.54% ± 0.1; 67% sign agreement) but transitions to an increase under higher emission pathways (+ 7.14% ± 1.8 under SSP5-8.5); Season B exhibits persistent decreases across all scenarios, ranging from − 14.46% ± 3.6 to -16.09% ± 4.0. Conversely, ELAZ and EATZ exhibit increases in both rainy seasons: ELAZ shows a Season B increase of up to + 10.47% ± 2.6 under SSP5-8.5 and a moderate Season A increase of + 5.43% ± 1.4, while EATZ shows a strong Season A intensification of + 11.51% ± 2.9 and a moderate Season B increase of + 2.93% ± 0.7 under SSP3-7.0. The Dry season shows the most dramatic projected precipitation declines across all AEZs, ranging from − 32.84% ± 8.2 in EHAZ to -82.65% ± 20.7 in THMAZ under SSP5-8.5, declines that are statistically robust with 100% sign agreement in all cases.

Monthly analysis (Figure S5) indicates that the wettest periods will likely shift or extend. Increases are projected for October–December (EHAZ, EMAZ, and THMAZ), and November–January (ELAZ, EATZ, and TLAZ). For Season B, localized increases are limited to February–March in most.

#### Seasonal changes in temperature

**Fig. 13:**
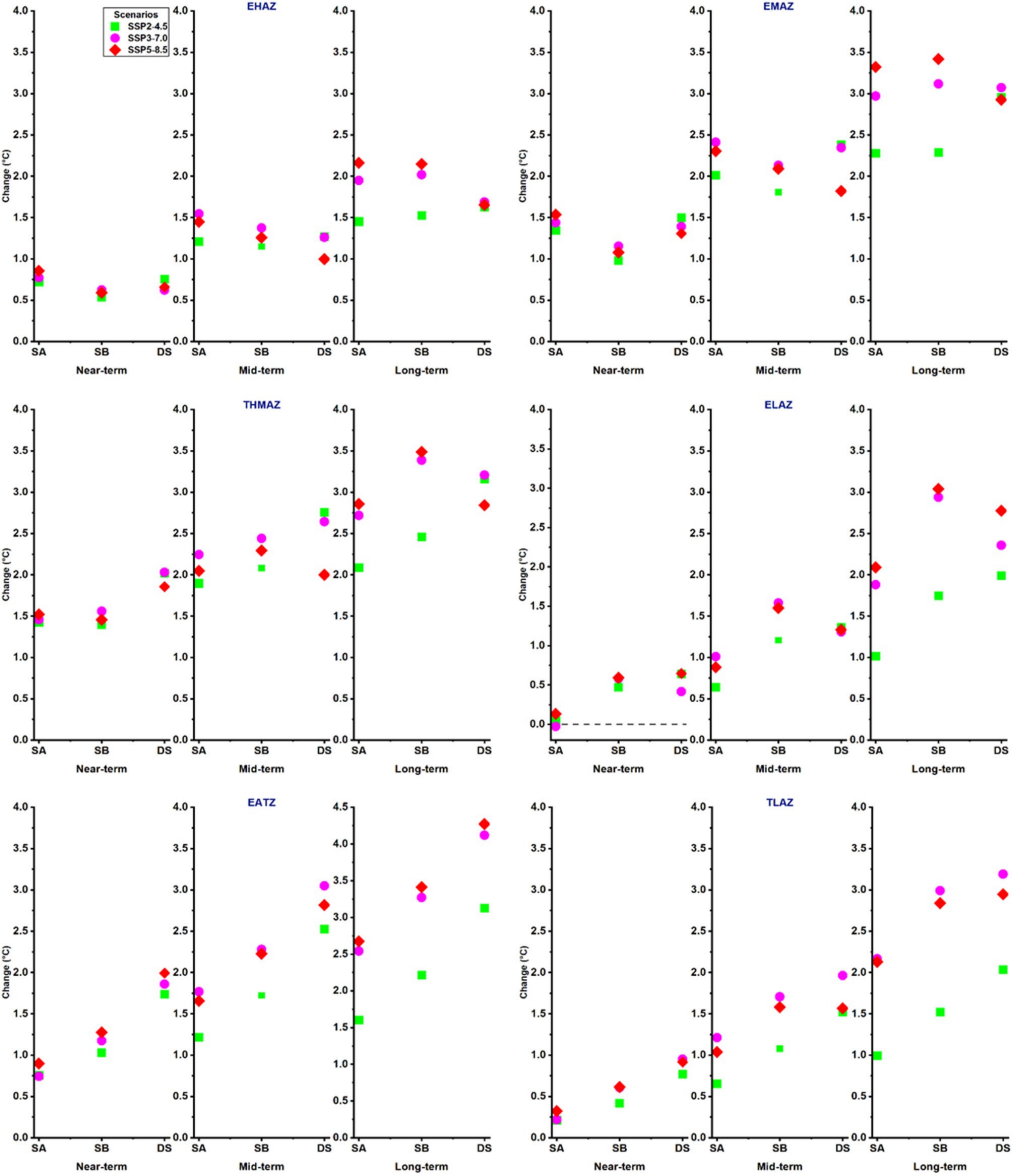
Projected seasonal changes in °C of maximum temperature in six AEZs at different time period scales in Near-term, Mid-term, and Long-term under SSP2-4.5, SSP3-7.0 and SSP5-8.5 from the Model Ensemble relative to the reference period of 1983–2014. SA: Season A; SB: Season B; and DS: Dry season.

**Fig. 14:**
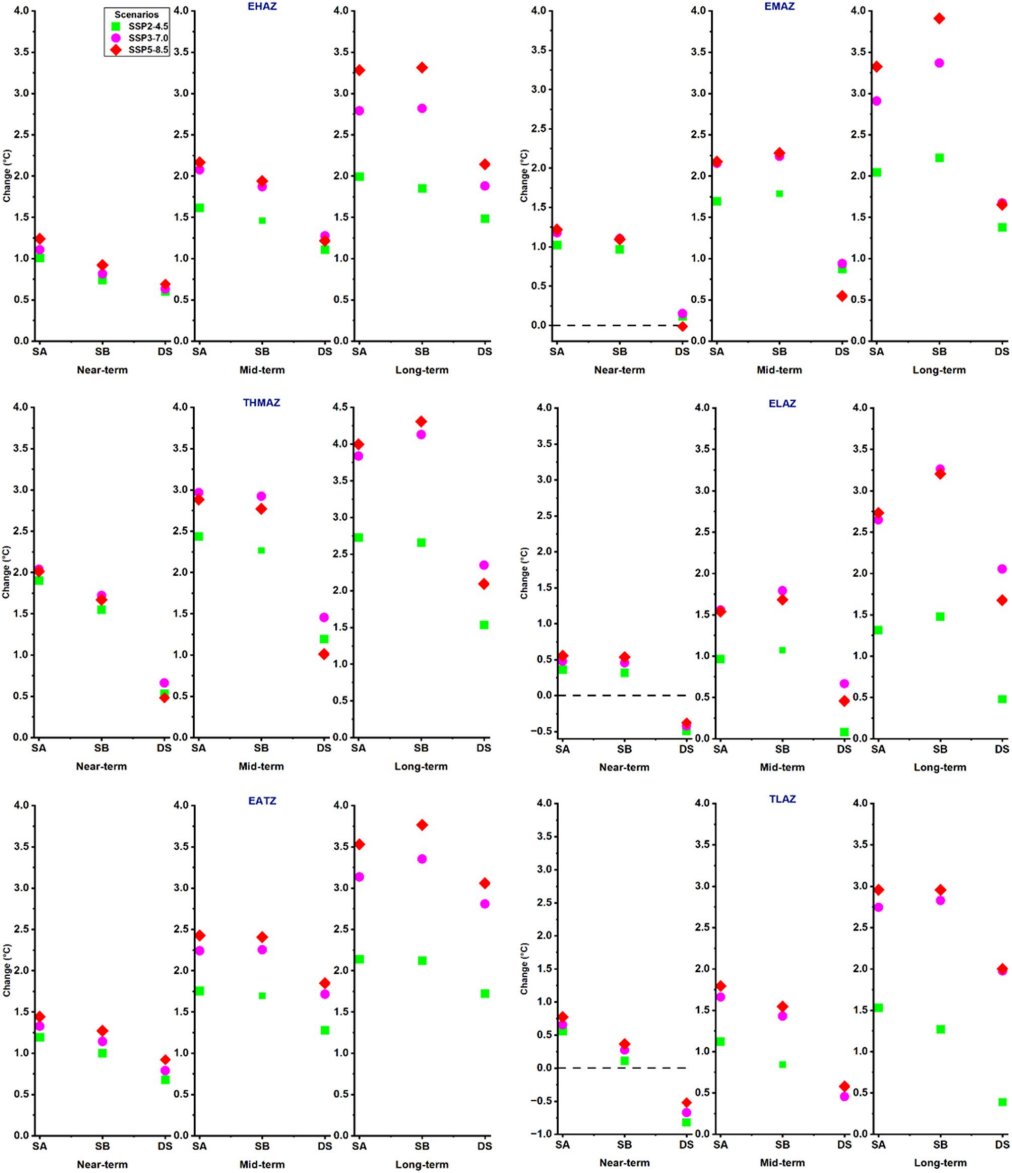
Projected seasonal changes in °C of minimum temperature in six AEZs at different time period scales in Near-term, Mid-term, and Long-term under SSP2-4.5, SSP3-7.0 and SSP5-8.5 from the Model Ensemble relative to the reference period of 1983–2014. SA: Season A; SB: Season B; and DS: Dry season.

Seasonal trajectories for Tmax and Tmin are presented in Figs. 13 and 14; Tables 8 and 9 and monthly changes are illustrated in Figures S5-S6. Warming is robust across all seasons, AEZs, and scenarios, with 100% sign agreement in all but two combinations. Under SSP5-8.5 long-term, Season B is projected to experience the most intense daytime warming in the EMAZ (+ 2.39 ± 0.31), THMAZ (+ 2.46 °C ± 0.32), and ELAZ (+ 1.74 °C ± 0.23). The Dry season is projected to see the highest warming in the EATZ (+ 3.03 °C ± 0.39). These shifts result in high-intensity monthly temperature peaks, with THMAZ and TLAZ projected to reach daytime maxima of 33.7 °C ± 0.29 to 34.0 °C ± 0.20 by April–May.

**Table 8:** Projected seasonal Tmax deltas across AEZs and SSP scenarios (2026–2100).

AEZs	Seasons	SSP2-4.5				SSP3-7.0				SSP5-8.5			
		**Mean delta (°C)**	**SD**	**95% CI**	**Sign**	**Mean delta (°C)**	**SD**	**95% CI**	**Sign**	**Mean delta (°C)**	**SD**	**95% CI**	Sign
EHAZ	Season A	1.13	0.15	[0.96, 1.30]	100	1.42	0.18	[1.21, 1.63]	100	1.49	0.19	[1.27, 1.71]	100
	Season B	1.07	0.14	[0.91, 1.23]	100	1.34	0.17	[1.14, 1.54]	100	1.33	0.17	[1.14, 1.52]	100
	Dry season	1.21	0.16	[1.03, 1.39]	100	1.19	0.15	[1.02, 1.36]	100	1.1	0.14	[0.94, 1.26]	100
EMAZ	Season A	1.88	0.24	[1.61, 2.15]	100	2.27	0.29	[1.94, 2.60]	100	2.2	0.29	[1.87, 2.53]	100
	Season B	1.69	0.22	[1.44, 1.94]	100	2.13	0.28	[1.81, 2.45]	100	2.39	0.31	[2.04, 2.74]	100
	Dry season	2.28	0.3	[1.94, 2.62]	100	2.27	0.3	[1.93, 2.61]	100	2.02	0.26	[1.72, 2.32]	100
THMAZ	Season A	1.8	0.23	[1.54, 2.06]	100	2.14	0.28	[1.82, 2.46]	100	2.14	0.28	[1.82, 2.46]	100
	Season B	1.98	0.26	[1.69, 2.27]	100	2.41	0.31	[2.06, 2.76]	100	2.46	0.32	[2.10, 2.82]	100
	Dry season	2.64	0.34	[2.25, 3.03]	100	2.63	0.34	[2.24, 3.02]	100	2.23	0.29	[1.90, 2.56]	100
ELAZ	Season A	0.56	0.07	[0.48, 0.64]	100	0.95	0.12	[0.81, 1.09]	100	1.03	0.13	[0.88, 1.18]	100
	Season B	1.14	0.15	[0.97, 1.31]	100	1.73	0.23	[1.47, 1.99]	100	1.74	0.23	[1.48, 2.00]	100
	Dry season	1.33	0.17	[1.14, 1.52]	100	1.36	0.18	[1.16, 1.56]	100	1.59	0.21	[1.35, 1.83]	100
EATZ	Season A	1.19	0.15	[1.02, 1.36]	100	1.68	0.22	[1.43, 1.93]	100	1.74	0.23	[1.48, 2.00]	100
	Season B	1.66	0.22	[1.41, 1.91]	100	2.24	0.29	[1.91, 2.57]	100	2.3	0.3	[1.96, 2.64]	100
	Dry season	2.46	0.32	[2.10, 2.82]	100	3.01	0.39	[2.57, 3.45]	100	3.03	0.39	[2.59, 3.47]	100
TLAZ	Season A	0.62	0.08	[0.53, 0.71]	100	1.2	0.16	[1.02, 1.38]	100	1.16	0.15	[0.99, 1.33]	100
	Season B	1.01	0.13	[0.86, 1.16]	100	1.77	0.23	[1.51, 2.03]	100	1.68	0.22	[1.43, 1.93]	100
	Dry season	1.44	0.19	[1.22, 1.66]	100	2.04	0.27	[1.73, 2.35]	100	1.81	0.24	[1.54, 2.08]	100

**Table 9:** Projected seasonal Tmin deltas across AEZs and SSP scenarios (2026–2100).

AEZs	Seasons	SSP2-4.5				SSP3-7.0				SSP5-8.5			
		**Mean delta (°C)**	**SD**	**95% CI**	**Sign**	**Mean delta (°C)**	**SD**	**95% CI**	**Sign**	**Mean delta (°C)**	**SD**	95% CI	Sign
EHAZ	Season A	1.54	0.21	[1.30, 1.78]	100	1.99	0.27	[1.68, 2.30]	100	2.23	0.31	[1.88, 2.58]	100
	Season B	1.35	0.19	[1.14, 1.56]	100	1.84	0.25	[1.56, 2.12]	100	2.06	0.28	[1.74, 2.38]	100
	Dry season	1.07	0.15	[0.90, 1.24]	100	1.26	0.17	[1.07, 1.45]	100	1.35	0.19	[1.14, 1.56]	100
EMAZ	Season A	1.59	0.22	[1.34, 1.84]	100	2.08	0.29	[1.75, 2.41]	100	2.24	0.31	[1.89, 2.59]	100
	Season B	1.66	0.23	[1.40, 1.92]	100	2.24	0.31	[1.89, 2.59]	100	2.43	0.34	[2.05, 2.81]	100
	Dry season	0.79	0.11	[0.67, 0.91]	100	0.92	0.13	[0.77, 1.07]	100	0.73	0.1	[0.62, 0.84]	100
THMAZ	Season A	2.36	0.33	[1.99, 2.73]	100	2.95	0.41	[2.49, 3.41]	100	2.91	0.41	[2.45, 3.37]	100
	Season B	2.16	0.3	[1.82, 2.50]	100	2.92	0.41	[2.46, 3.38]	100	2.96	0.41	[2.50, 3.42]	100
	Dry season	1.09	0.15	[0.92, 1.26]	100	1.49	0.21	[1.25, 1.73]	100	1.2	0.17	[1.01, 1.39]	100
ELAZ	Season A	0.88	0.12	[0.74, 1.02]	100	1.56	0.22	[1.31, 1.81]	100	1.61	0.23	[1.35, 1.87]	100
	Season B	0.96	0.13	[0.81, 1.11]	100	1.84	0.26	[1.55, 2.13]	100	1.81	0.25	[1.53, 2.09]	100
	Dry season	0.02	0.01	[0.01, 0.03]	67	0.77	0.11	[0.65, 0.89]	100	0.59	0.08	[0.50, 0.68]	100
EATZ	Season A	1.7	0.24	[1.43, 1.97]	100	2.23	0.31	[1.88, 2.58]	100	2.47	0.35	[2.07, 2.87]	100
	Season B	1.61	0.23	[1.35, 1.87]	100	2.25	0.32	[1.89, 2.61]	100	2.48	0.35	[2.08, 2.88]	100
	Dry season	1.23	0.17	[1.04, 1.42]	100	1.77	0.25	[1.49, 2.05]	100	1.94	0.27	[1.63, 2.25]	100
TLAZ	Season A	1.07	0.15	[0.90, 1.24]	100	1.69	0.24	[1.42, 1.96]	100	1.84	0.26	[1.55, 2.13]	100
	Season B	0.74	0.1	[0.63, 0.85]	100	1.51	0.21	[1.27, 1.75]	100	1.62	0.23	[1.36, 1.88]	100
	Dry season	-0.19	0.03	[-0.22, -0.16]	100	0.58	0.08	[0.49, 0.67]	100	0.59	0.08	[0.50, 0.68]	100

Nighttime temperatures show progressive and unanimous increases (100% sign agreement in all but one combination), with the highest seasonal warming during Season B. By the late century under SSP5-8.5, Season B nighttime peaks are projected at + 2.96 °C ± 0.41 (THMAZ), + 2.43 °C ± 0.34 (EMAZ), and + 2.48 °C ± 0.35 (EATZ). Monthly nighttime peaks are projected to exceed 28 °C ± 0.33 in the THMAZ and 25° ± 0.18 in the ELAZ. Nighttime temperatures are also projected to reach unprecedented levels, with monthly peaks exceeding 28 °C ± 0.33 in the THMAZ and 25 °C ± 0.18 in the ELAZ. TLAZ Dry season projects a slight cooling in minimum temperatures under SSP2-4.5 (-0.19 °C ± 0.03; 100% sign agreement). Under SSP3-7.0 and SSP5-8.5, this cooling signal disappears and warming prevails (+ 0.58–0.59 °C). These results confirm that while daytime thermal shifts are critical for crop physiological limits, amplified nighttime warming during the growing seasons represents the most significant and spatially consistent thermal stress for regional agroecosystems.

## Discussion

This study provides a characterization of climate trajectories across six topographically complex AEZs in South Kivu. Historical climate trajectories in South Kivu are defined by a decoupling between thermal and hydrological signals. While annual precipitation exhibits predominantly negative but statistically non-significant trends (p ˃ 0.05) across five of six AEZs, temperature trends show consistent, statistically significant warming (Tmax: +0.015 °C yr^− 1^ to + 0.016 °C yr^− 1^; *p* < 0.05). This divergence, where significant temperature increases coexist with spatially heterogeneous and largely stable rainfall, aligns with broader East African warming trajectories^1,2,57^ and confirms that thermodynamic coupling is not the primary driver of regional precipitation variability. The spatial distribution of observed precipitation shifts reveals a height-dependent drying signal. The EHAZ was the only zone exhibiting a statistically significant annual decline (-4.518 mm yr^− 1^, *p* = 0.043), corroborating the high-altitude drying signal reported by Ahana et al.^27^. Conversely, the relative stability observed in the THMAZ (+ 0.295 mm yr^− 1^; *p* = 0.884) is consistent with localized assessments in the adjacent Ruzizi catchment^25^. Seasonally, precipitation declines are most concentrated during the short rainy season (Season B, February-May), suggesting a temporal contraction of moisture availability that, despite its marginal statistical significance, poses substantial risks to the region’s smallholder-dominated agricultural stability^5,6,8,10^. The observed weak inverse association between annual temperature and precipitation (R^2^ from 0.2% to 14%) further confirms that regional rainfall is not primarily thermodynamically driver at inter-annual scales. While the 14% shared variance in the ELAZ is consistent with land-atmosphere feedback loops, whereby elevated temperatures enhance Vapor Pressure Deficit (VPD) and modulate convective initiation^97–99^; this relationship is secondary to a complex multivariate interaction of moisture transport, atmospheric stability, and topographic forcing^100–102^. The limited explanatory power of inter-annual temperature averages is further illustrated by the high temporal intermittency of tropical rainfall, where according to Pendergrass and Knutti^103^, a small fraction of days often accounts for the majority of annual totals. These findings reinforce the premise that single-variable linear frameworks systematically oversimplify tropical climate dynamics. In South Kivu, temperature functions as one among several convective precursors rather than a primary forcing variable^104^.

The observed AEZ-level precipitation heterogeneity exhibits patterns broadly consistent with established regional climate drivers, including large-scale atmospheric circulation, orographic uplift, and Congo Basin moisture dynamics^56,105^. While our analysis does not provide a formal attribution of these mechanisms, which would require moisture flux convergence or vertically integrated moisture transport (VIMT) diagnostics, the AEZ-specific precipitation totals align with the conceptual framework of the Intertropical Convergence Zone (ITCZ) as a primary moisture transport mechanism^106^. For instance, the high annual totals in the EATZ (2383 mm) are geographically coincident with the first major topographic barrier along the western moisture pathway, where orographic enhancement is theoretically maximized. Conversely, the moderate rainfall in the high-altitude EHAZ and EMAZ, despite their summit elevations exceeding 3000 m, may reflect progressive moisture depletion along the windward slopes. The minimum rainfall observed in the THMAZ (1204 mm) is consistent with the expected rain-shadow effects documented in comparable rift settings^56,105^. While these associations provide a plausible interpretive context, further investigation is required to decouple the relative contributions of large-scale forcing, orographic modulation, and localized land-surface feedbacks.

Selecting GCMs for regional climate impact assessments remains a primary challenge, as projections are subject to both internal variability and epistemic uncertainties arising from divergent structural representations of physical processes^36,37,39^. These deficiencies are particularly acute in topographically complex regions^36–39^, where coarse-resolution models often fail to resolve mesoscale convective interactions and orographic moisture transport^40^. To systematically manage these uncertainties, we adopted a performance-based ensemble selection designed to minimize errors attributable to model structural deficiencies^2,30,47,49,57,68^. Model performance exhibited significant spatial, reinforcing the principle that AEZ-specific selection is essential for resolving climate trajectories in topographically complex domains^32,49,68^. While MRI-ESM2-0, GFDL-ESM4, and MPI-ESM1-2-HR demonstrated broad reliability, zonal variations in skill, such as BCC-CSM2-MR’s superior performance in low-altitude zones versus CMCC-CM2-SR5 in high- and mid-altitude zones, reflect the differential capacity of GCMs to represent regional moisture recycling and orographic gradients^2,30,47,49,57,68^. By isolating models that minimize these structural deficiencies, our ensemble extracts a physically-consistent climate signal from model-specific epistemic noise^30,32,56,57^. This signal integrity is corroborated by our preservation analysis, which confirms that the PQM framework maintains the original externally forced change signal. Across all 54 AEZs-scenario-period combinations, absolute deviation remained below 0.72 pp for Q95 (Figure S3) and 0.31 pp for wet-day frequency, with sign of change preserved in 100% of cases. We acknowledge that fidelity of this evaluation is critically anchored to the observational benchmark. CHIRPS and CHIRTS inherent uncertainties regarding orographic precipitation and thermal heterogeneity propagate into the total uncertainty budget, influencing the absolute calibration bias-correction functions and the magnitude of performance metrics^62,64,65^. However, the capacity of the selected ensemble to resolve observed moisture gradients and seasonal phasing suggests that the benchmark successfully identifies models with superior structural skill, providing a reliable, physically-grounded basis for AEZ-scale climate assessment^57,107^.

The methodological utility of our selection framework is underscored by the high-ranking stability observed across independent validation windows (Tables S5-S9). Through a symmetric two-fold-cross-validation, the selected GCMs exhibited a weighted mean stability index (SI) of 0.83 for precipitation, 0.94 for Tmax and 1.00 for Tmin (Tables S8-S9). This persistence suggests that model skill in capturing South Kivu’s geoclimatic drivers is a structural feature of the selected GCMs cores rather than a temporal artifact. Crucially, the two validation windows (1983–1998 and 1999–2014) are not climatologically equivalent; each encompasses a distinct phases of regional moisture variability, including the predominantly La Niña-associated dry phase and the post-1999 Indian Ocean Dipole-positive (IOD) wetting trend^108^. High stability across these climatologically contrasting epochs provides a more rigorous robustness test than windows drawn from similar climatic conditions, confirming that the Top-3 models resolve persistent physical features of the regional climate system^27,56^. Furthermore, as these models consistently achieved the highest TSS, alongside the lowest PBIAS and RMSE, the ensemble remains optimal across multiple performance dimensions simultaneously^32,32,47,49,92,93^. The sensitivity check comparing the Top-3 ensemble against an expanded Top-5 MME (Tables S10-S12) provides a diagnostic justification for a constrained ensemble approach, particularly in AEZs where the forced climate signal is small relative to model divergence.

Precipitation projections exhibit significant sensitivity to ensemble membership (Table S10). The expansion to a Top-5 ensemble introduces substantial shifts across several AEZs. EATZ is high-confidence since it remained stable; EMAZ and EHAZ are moderate-confidence due to magnitude sensitivity (divergence 2.2–2.7 pp). Despite these magnitude shifts, these zones maintained directional coherence, suggesting a consistent drying signal among lower-ranked members. In contrast, THMAZ, ELAZ, and TLAZ exhibited profound structural sensitivities, where Top-5 inclusion displaced the ensemble mean by up to 7.2 times the Top-3 internal spread. Critically, this displacement induced sign reversals in 15% of precipitation scenario-period combinations. We interpret these reversals not as valid climatic uncertainty, but as evidence of idiosyncratic noise introduced by models whose structural deficiencies obscure the externally forced signal. This high sensitivity along near-neutral boundaries confirms that ensemble spread is function of the screening criteria; however, relaxing these criteria to achieve statistical neutrality risk absorbing biases that lack physical consistency with regional geoclimatic drivers. Consequently, in these sensitive zones, projections should be treated as indicative central-tendency estimates within a wider structurally uncertain envelope, requiring higher risk margins in adaptation planning.

Projected temperature shifts exhibit a clear decoupling between Tmin and Tmax in terms of ensemble sensitivity (Tables S11-S12). For Tmin, the Top-3 ensemble remains robust across four AEZs, with mean shifts (± 0.13 °C) consistently below our 0.2 °C threshold. Even in the THMAZ, where a higher thermal offset was observed (+ 0.58 to 0.$\:62^\circ\:C$), the absence of sign reversals and the uniform direction of bias suggests high-confidence trajectories across all six AEZs. In contrast, Tmax projections reveal profound structural sensitivities in low-altitude zones, where the expansion to a Top-5 ensemble induces diverge bias patterns. In the EHAZ, lower-ranked models introduce a systematic warm bias (+ 0.42–0.49 °C), likely reflecting poorly constrained elevation-dependent warming (EDW) feedbacks^109^. More critically, the ELAZ exhibits a structurally opposite bias, where lower-ranked models are consistently cooler than the Top-3, leading to sign reversals in all near-term scenarios. We contend that these sign reversals projecting near-term cooling in an equatorial lowland zone represent a non-physical artifact of specific land-surface coupling errors in lower-performing models. Given the categorical evidence for continued warming across Africa^2,110,111^ and documented coupling deficiencies in certain CMIP6 models at low elevations^112,113^, a cooling projection is physical implausible and reflects model structural failure rather than genuine climatic uncertainty^49,57^. The fact that ELAZ is sensitive in Tmax but robust in Tmin reinforces the premise that model deficiencies manifest selectively across variables^47,57,93^. This diagnostic evidence justifies the performance-based selection as a necessary filter to prevent idiosyncratic noise from undermining the physical plausibility of regional projections^14,32^.

Our results align with Knutti and Sedláček^114^, who demonstrated that increasing ensemble size in CMIP generations does not inherently reduce inter-model spread, and with Hawkins and Sutton^115^, who identified model structural uncertainty as the primary driver of regional projection spread. Consequently, expanding an ensemble without performance-based screening risks compounding epistemic uncertainty by absorbing idiosyncratic structural biases that obscure the forced climate signal. We acknowledge that our Top-3 approach, by construction, yields narrower uncertainty bounds than an unvetted ensemble; however, our sensitivity analysis confirms that this constraint is a prerequisite for physical plausibility in complex terrain. The performance-constrained approach aligns with established methods for resolving regional signal fidelity^47,49,93,107^. For instance, Sa’adi et al.^111^ demonstrated that retaining only the most skillful models can narrowed uncertainty without sacrificing physical plausibility. Our framework validated through cross-validation stability and shown to be spatially and temporally consistent across 40 of 54 AEZ-scenario-period combinations identifies the specific GCM subset capable of resolving South Kivu geoclimatic drivers. By identifying the remaining 14 combinations as high-divergence cases, where structural uncertainty remains dominant over the forced signal, we provide a diagnostic of the limits of projection confidence. This characterization of uncertainty, rather than an exhaustive multi-model mean, aligns with best practices for localized climate assessment in data-scarce regions^32,32,47,49,92,93^.

The projected trajectories for South Kivu represent a physically-consistent intensification of the significant historical warming identified in our baseline analysis. While historical trends were dominated by daytime increases (+ 0.015 to + 0.016 °C yr^− 1^), future projections under SSP5-8.5 suggest a transition toward greater absolute increase in nighttime temperatures (Tmin of up to 3.89 °C), reversing the historical asymmetric warming pattern where Tmin remained largely non-significant. These magnitudes align with the projected ranges of + 1.4 °C to 4.4 °C for Africa^2^ and are consistent within the 1.7–4.5 °C increase projected for the DRC^7^. Notably, the greater nocturnal warming relative to Tmax mirrors the greater tropical response to intensified greenhouse forcing and cloud radiative feedback^116,117^. This narrowing of the diurnal temperature range (DTR) is physically driven by trapped longwave radiation within the planetary boundary layer, a mechanism consistent with regional assessments over East Africa^57,118,119^. Our AEZ-specific approach reveals critical intrazonal heterogeneity. The EMAZ shows amplified daytime warming (up to 3.38 °C ± 0.56 under SSP5-8.5), exceeding the lowland ELAZ (2.31 °C) despite its higher elevation. This projection extends the significant Tmax increases already detected in the historical record, and potentially signals the early stages of elevation-dependent warming (EDW) feedbacks^120,121^. EDW is well documented in the Albertine Rift, where land-surface feedbacks regulate temperature responses through coupled albedo and evapotranspiration shifts^122^. However, we acknowledge that this high-altitude amplification may be compounded by orographic parameterization errors in coarse-resolution GCMs. Furthermore, the high nighttime warming for the THMAZ corroborates localized findings in the Ruzizi catchment the findings in the Ruzizi catchment^25^, confirm the regional coherence of the warming signal across adjacent study domains.

Precipitation remains the most volatile component of the region’s climate trajectory, exhibiting divergent and non-linear trajectories that do not scale linearly with projected warming. This this mirrors the weak thermodynamic coupling identified in our historical analysis, where temperature explained only up to 14% of annual precipitation variance. Consequently, future shifts are likely dominated by changes in large-scale atmospheric circulation and moisture transport rather than local thermal forcing. While South Kivu generally follows a pattern of near-term decline followed by late-century intensification, consistent with findings in the northeastern DRC^56^ and Central-East Africa^123^, significant intrazonal disparities persist. The EMAZ exhibits persistent long-term declines (up to -9.8%), representing an intensification of the negative trend already observed in the historical record. Conversely, the THMAZ shows a dramatic late-century wetting signal (+ 21% ± 8.14), exceeding CMIP5-based estimates (+ 14.7%)^25^. This amplification likely reflects higher climate sensitivity of CMIP6 compared to their predecessors^2,30,47,49,57,68^. While the direction of the THMAZ wetting trend is robust within our Top-3 ensemble, its magnitude is highly sensitive to individual model parameterization. Crucially, we acknowledge that the true structural uncertainty is wider than our screened ensemble spread; for the THMAZ, the full seven-model distribution spans − 8% to + 34%, highlighting that projection confidence is lowest where model structural divergence is largest. This inherent uncertainty is consistent with projection spreads documented across comparable high-altitude African domains^32,49,124^.

Beyond annual shifts, the projected seasonal redistribution signals a significant alteration of regional hydro-climatic constraints. The dry season (June-August) exhibits the most severe and statistically robust precipitation declines across all AEZs, while the bimodal rainy seasons show a divergent response: intensification during Season A (September-January) and contraction during Season B (February-May), particularly in high-altitude zones (Fig. 12). This divergence is consistent with regional dynamics where the October-December rains, heavily modulated by the IOD and El Niño‒Southern Oscillation (ENSO) are projected to experience greater intensification than the March‒May season^59^. The projected contraction of the short rainy season increases the regional dependence on a single growing period, potentially amplifying agricultural exposure in a domain already vulnerable to multi-hazard climate stress^6,8,9,26^. While our analysis characterizes the physical climate drivers, quantifying the precise thresholds at which these trajectories cross critical productivity limits remain a priority for future integrated assessment. By providing a physically-consistent forcing baseline, this study establishes the necessary foundation for the process-based crop and hydrological modeling required to translate AEZ-scale climate signals into actionable adaptation thresholds.

### Implications of the study

The projected climate trajectories across South-Kivu represent shifts in the physical boundary conditions of regional agroecosystems. The warming trends identified, with Tmax rising by up to 3.38 °C ± 0.56 in the EMAZ and Tmin peaking at + 3.89 °C ± 1.02 in the THMAZ under SSP5-8.5 signal a plausible intensification of evaporative demand. In the absence of targeted adaptation, these thermal trajectories represent an increased exposure to heat stress, potentially reducing the suitability envelope for staple crops across all AEZs. In the high-altitude zones (EHAZ and EMAZ), the projected EDW may alter the production niche for traditional cool-climate crops^125^. However, we emphasize that specific yield penalties would be contingent on genotype-specific responses and CO2 fertilization effect, which is beyond the scope of this study. The EMAZ, with its persistent drying trajectory emerges as a priority zone for investigating compounded drought and thermal stress. Furthermore, the established literature for the region^12–14^ suggests that the projected warming provides the physical boundary conditions for potential upward expansion of ecological niches for thermophilic pests and vectors; though confirmed shifts would require dynamic species distribution modelling beyond the current physical climate assessment. The contraction of Season B signals a transition risk for planting calendar management across high-altitude zones^6,25^. In the lowland and transitional zones (ELAZ, EATZ, TLAZ), late-century precipitation intensification increases exposure to soil saturation and run-off-driven nutrient loss; in THMAZ, the + 21% wetting signal under SSP5-8.5 indicates heightened episodic flood risk, particularly if the increase is concentrated within Season A.

These AEZ-differentiated signals highlight the necessity for zone-targeted risk assessments. While this study does not directly model agronomic outcomes, the climatic evidence indicates a significant decoupling of historical practices from future atmospheric realities. The AEZ-scale trajectories and associated uncertainty ranges produced here represent the most physically-screened climate forcing dataset currently available for South Kivu, providing the necessary inputs for future process-based crop simulations, hydrological frameworks and land-use policy. Decision-makers should prioritize high-confidence signals when formulating near-term adaptation strategies, while treating structurally sensitive signals as high-risk indicators requiring wider safety margins. Translating these physical signals into specific adaptation thresholds through integrated impact modeling remains the essential next step for localized planning in South Kivu.

## Conclusion

This study provides a high-resolution characterization of climate change trajectories across South Kivu’s agroecological zones (AEZs) using a performance-based CMIP6 ensemble. By implementing a symmetric two-fold cross-validation and a variable-specific selection framework, we demonstrate that model skill in resolving regional geoclimatic drivers represents a persistent structural feature rather than a temporal artifact. Our selected ensemble members consistently reproduce regional orographic climate dynamics across climatologically distinct windows, achieving a weighted mean stability index of 0.83 for precipitation and 0.94-1.00 for temperature. MRI-ESM2-0, GFDL-ESM4, and MPI-ESM1-2-HR performed best overall, though pronounced zonal variations were observed. Furthermore, our sensitivity analysis confirms that ensemble membership expansion introduces idiosyncratic biases and physically implausible sign reversals, reinforcing the necessity of a performance-filtered Top-3 subset to isolate a consistent climate signal from structural model noise.

Historically, the province is defined by a thermodynamic decoupling, where significant warming signal coexists with largely non-significant precipitations decline. Our projections indicate a systematic intensification of these trajectories through 2100. Under the SSP5-8.5, thermal amplification reaches 3.38 °C ± 0.56 in the EMAZ, and + 3.89 °C ± 1.02 in the THMAZ, signaling a potential narrowing of the diurnal temperature range and the emergence of elevation-dependent warming feedbacks. Precipitation projections remains non-linear and spatially divergent, following a pattern of near-term drying followed by a late-century intensification across most zones. EMAZ maintains a persistent drying trajectory (reaching − 9.8%), while the THMAZ shows a dramatic late-century wetting signal (+ 21.2 ± 8.14), highlighting the extreme topographic modulation of the region’s future moisture regime.

These projections are best interpreted within the context of the three specific limitations:

i. Satellite-derived observational products may introduce systematic biases regarding orographic rainfall and subgrid-scale thermal heterogeneity over the Mitumba Range,
ii. The attribution of precipitation dynamics to ITCZ migration and rain-shadow effects remains interpretive pending VIMT diagnostics.
iii. Empirical QM assumes stationarity of the statistical transfer function, which may not hold under high-emission pathways where projected distributional shifts exceed the historical calibration range.

Ultimately, the AEZ-tailored framework developed here demonstrates that performance-based GCM selection can effectively isolate reliable forced signals from idiosyncratic structural noise. This study provides a physically-screened climate forcing baseline that characterizes the range of plausible trajectories across South Kivu’s diverse landscapes. Translating these physical signals into specific adaptation thresholds through process-based crop and hydrological simulations remains the essential next step for understanding localized climate vulnerability in the Albertine Rift and analogous tropical montane systems.

## Supplementary Information

Below is the link to the electronic supplementary material.

Supplementary Material 1

## Data Availability

**Materials should be addressed to L.C.K upon a reasonable request**.
