# Supplementary material for: Climate change scenarios across South-Kivu agroecological zones, Eastern D.R. Congo
Source: Sci Rep. 2026 May 3;16:20452. doi: 10.1038/s41598-026-50143-8 (PMC13332071; doi:10.1038/s41598-026-50143-8)
Supplement: Supplementary file 1 — Supplementary Material 1 [file 41598_2026_50143_MOESM1_ESM.docx]

**Supplementary Tables**

**Table S1. Homogeneity test results for precipitation and temperature across AEZs (1983-2014)**

| Variable | AEZ | Pettitt's *K* | p-value | Breakpoints | Year | Month | Shift Magnitude |
| --- | --- | --- | --- | --- | --- | --- | --- |
| Precipitation (mm/month) | EHAZ | 3980 | 0.375 | No |  |  |  |
|  | EMAZ | 1705 | 1 | No |  |  |  |
|  | THMAZ | 2141 | 1 | No |  |  |  |
|  | ELAZ | 2338 | 1 | No |  |  |  |
|  | EATZ | 2583 | 0.988 | No |  |  |  |
|  | TLAZ | 2607 | 0.975 | No |  |  |  |
| Tmax (°C) | EHAZ | 10404 | <0.001*** | Yes | 1993 | June | 0.494 |
|  | EMAZ | 8170 | 0.002** | Yes | 1993 | June | 0.45 |
|  | THMAZ | 8878 | <0.001*** | Yes | 1993 | June | 0.46 |
|  | ELAZ | 12460 | <0.001*** | Yes | 1995 | April | 0.448 |
|  | EATZ | 11262 | <0.001*** | Yes | 1995 | April | 0.478 |
|  | TLAZ | 9404 | <0.001*** | Yes | 1995 | April | 0.428 |
| Tmin (°C) | EHAZ | 5857 | 0.053 | No |  |  |  |
|  | EMAZ | 4647 | 0.204 | No |  |  |  |
|  | THMAZ | 4596 | 0.215 | No |  |  |  |
|  | ELAZ | 6497 | 0.023* | Yes | 2006 | May | -0.306 |
|  | EATZ | 7172 | 0.009** | Yes | 1990 | April | 0.382 |
|  | TLAZ | 4488 | 0.238 | No |  |  |  |

p < 0.05, p < 0.01**, p < 0.001***

**Table S2. Model performance metrics for precipitation across agroecological zones on Fold 1 validation (1999-2014) and Fold 2 validation (1983-1998)**

| **AEZ** | **Model** | **Fold 1** | | | **Fold 2** | | |
| --- | --- | --- | --- | --- | --- | --- | --- |
|  |  | **RMSE (mm/month)** | **PBIAS (%)** | **TSS** | **RMSE (mm/month)** | **PBIAS (%)** | **TSS** |
| EHAZ | BCC-CSM2-MR | 37.8 | -20.9 | 0.588 | 53.24 | -27.33 | 0.373 |
|  | CMCC-CM2-SR5 | 19.2 | -4.4 | 0.774 | 30.85 | -8.01 | 0.531 |
|  | GFDL-ESM4 | 27.6 | -12.5 | 0.675 | 40.62 | -17.21 | 0.446 |
|  | MIROC6 | 32.4 | -15.4 | 0.628 | 46.44 | -20.44 | 0.398 |
|  | MPI-ESM1-2-HR | 25.8 | -11.9 | 0.712 | 37.91 | -9.16 | 0.490 |
|  | MRI-ESM2-0 | 38.8 | -21.1 | 0.565 | 54.13 | 3.92 | 0.323 |
| EMAZ | BCC-CSM2-MR | 30.6 | -10.1 | 0.610 | 45.16 | -14.53 | 0.399 |
|  | CMCC-CM2-SR5 | 15.8 | 0.1 | 0.730 | 24.26 | 4.14 | 0.520 |
|  | GFDL-ESM4 | 24.4 | -12.4 | 0.675 | 36.34 | -16.04 | 0.447 |
|  | MIROC6 | 31.8 | -16.9 | 0.644 | 48.78 | -22.18 | 0.368 |
|  | MPI-ESM1-2-HR | 17.6 | -8.1 | 0.774 | 27.21 | -12.67 | 0.551 |
|  | MRI-ESM2-0 | 29.2 | -12.9 | 0.700 | 40.18 | -17.05 | 0.435 |
| THMAZ | BCC-CSM2-MR | 24.9 | -13.1 | 0.653 | 39.48 | -20.11 | 0.359 |
|  | CMCC-CM2-SR5 | 18.6 | 13.4 | 0.614 | 52.16 | 19.74 | 0.368 |
|  | GFDL-ESM4 | 13.2 | -3.9 | 0.711 | 23.38 | -8.40 | 0.505 |
|  | MIROC6 | 38.1 | 13.4 | 0.676 | 30.09 | 22.28 | 0.447 |
|  | MPI-ESM1-2-HR | 10.1 | 2.2 | 0.755 | 16.42 | 7.10 | 0.556 |
|  | MRI-ESM2-0 | 23.8 | -13.4 | 0.676 | 36.31 | -18.44 | 0.426 |
| ELAZ | BCC-CSM2-MR | 22.1 | -7.1 | 0.749 | 28.24 | -10.36 | 0.709 |
|  | CMCC-CM2-SR5 | 23.1 | -8.4 | 0.730 | 29.46 | -12.72 | 0.675 |
|  | GFDL-ESM4 | 22.4 | -3.9 | 0.744 | 30.11 | -7.48 | 0.661 |
|  | MIROC6 | 29.1 | -12.4 | 0.690 | 39.83 | -18.41 | 0.604 |
|  | MPI-ESM1-2-HR | 29.8 | -12.1 | 0.730 | 36.09 | -16.93 | 0.644 |
|  | MRI-ESM2-0 | 18.8 | 5.2 | 0.774 | 27.21 | 9.22 | 0.695 |
| EATZ | BCC-CSM2-MR | 19.1 | -3.4 | 0.675 | 28.66 | -7.16 | 0.628 |
|  | CMCC-CM2-SR5 | 15.2 | 4.4 | 0.772 | 22.14 | 8.42 | 0.695 |
|  | GFDL-ESM4 | 12.1 | 1.4 | 0.789 | 18.36 | 4.96 | 0.714 |
|  | MIROC6 | 22.8 | 6.9 | 0.700 | 31.90 | 11.04 | 0.641 |
|  | MPI-ESM1-2-HR | 23.8 | 7.9 | 0.716 | 34.51 | 13.71 | 0.601 |
|  | MRI-ESM2-0 | 17.8 | -5.4 | 0.730 | 26.33 | -9.53 | 0.661 |
| TLAZ | BCC-CSM2-MR | 18.9 | -8.9 | 0.747 | 24.90 | -12.66 | 0.571 |
|  | CMCC-CM2-SR5 | 15.1 | -3.9 | 0.684 | 21.12 | -7.24 | 0.502 |
|  | GFDL-ESM4 | 33.6 | -17.1 | 0.670 | 46.78 | -22.17 | 0.473 |
|  | MIROC6 | 38.1 | 16.1 | 0.641 | 54.08 | 21.63 | 0.371 |
|  | MPI-ESM1-2-HR | 16.2 | 3.4 | 0.724 | 22.41 | 7.83 | 0.523 |
|  | MRI-ESM2-0 | 20.1 | -8.9 | 0.703 | 28.65 | -13.30 | 0.536 |

**Table S3. Model performance metrics for maximum temperature across agroecological zones on Fold 1 validation (1999-2014) and Fold 2 validation (1983-1998)**

| **AEZ** | **Model** | **Fold 1** | | | **Fold 2** | | |
| --- | --- | --- | --- | --- | --- | --- | --- |
|  |  | **RMSE (°C/month)** | **PBIAS (%)** | **TSS** | **RMSE (°C/month)** | **PBIAS (%)** | **TSS** |
| EHAZ | AWI-CM-1-1-MR | 2.09 | 3.44 | 0.663 | 2.79 | 4.33 | 0.502 |
|  | GFDL-ESM4 | 0.68 | 1.96 | 0.825 | 0.99 | 3.82 | 0.660 |
|  | MIROC6 | 1.18 | 3.96 | 0.794 | 1.63 | 5.61 | 0.615 |
|  | MPI-ESM1-2-HR | 0.52 | -1.66 | 0.841 | 0.79 | -3.24 | 0.697 |
|  | MRI-ESM2-0 | 0.22 | 0.42 | 0.872 | 0.37 | 1.17 | 0.741 |
| EMAZ | AWI-CM-1-1-MR | 1.38 | 1.77 | 0.898 | 1.94 | 2.97 | 0.732 |
|  | GFDL-ESM4 | 0.87 | 0.96 | 0.856 | 1.38 | 1.54 | 0.719 |
|  | MIROC6 | 2.38 | 3.55 | 0.843 | 3.09 | 5.12 | 0.663 |
|  | MPI-ESM1-2-HR | 1.38 | 1.77 | 0.898 | 1.96 | 2.44 | 0.748 |
|  | MRI-ESM2-0 | 1.61 | 2.58 | 0.852 | 2.36 | 3.88 | 0.684 |
| THMAZ | AWI-CM-1-1-MR | 0.68 | 1.56 | 0.901 | 1.07 | 2.88 | 0.756 |
|  | GFDL-ESM4 | 0.99 | 0.72 | 0.926 | 1.44 | 1.63 | 0.777 |
|  | MIROC6 | 0.81 | 2.38 | 0.874 | 1.41 | 3.41 | 0.727 |
|  | MPI-ESM1-2-HR | 0.68 | 1.56 | 0.901 | 0.98 | 2.31 | 0.765 |
|  | MRI-ESM2-0 | 1.58 | 2.47 | 0.874 | 1.97 | 3.72 | 0.704 |
| ELAZ | AWI-CM-1-1-MR | 0.44 | 1.34 | 0.870 | 0.71 | 2.14 | 0.775 |
|  | GFDL-ESM4 | 0.68 | -2.17 | 0.782 | 0.98 | -3.41 | 0.628 |
|  | MIROC6 | 0.78 | -2.64 | 0.499 | 1.12 | -4.07 | 0.371 |
|  | MPI-ESM1-2-HR | 0.42 | -1.07 | 0.945 | 0.62 | -1.88 | 0.823 |
|  | MRI-ESM2-0 | 0.36 | 0.98 | 0.935 | 0.56 | 1.74 | 0.820 |
| EATZ | AWI-CM-1-1-MR | 0.56 | 1.97 | 0.879 | 0.82 | 2.87 | 0.766 |
|  | GFDL-ESM4 | 0.29 | 0.9 | 0.901 | 0.47 | 1.34 | 0.804 |
|  | MIROC6 | 0.68 | 2.41 | 0.874 | 0.99 | 3.54 | 0.735 |
|  | MPI-ESM1-2-HR | 0.28 | 0.88 | 0.916 | 0.44 | 1.22 | 0.822 |
|  | MRI-ESM2-0 | 0.52 | 1.76 | 0.926 | 0.72 | 2.43 | 0.816 |
| TLAZ | AWI-CM-1-1-MR | 2.04 | 3.64 | 0.575 | 2.81 | 4.91 | 0.463 |
|  | GFDL-ESM4 | 0.99 | -1.36 | 0.916 | 1.29 | -2.12 | 0.802 |
|  | MIROC6 | 1.06 | -2.02 | 0.870 | 1.56 | -3.17 | 0.732 |
|  | MPI-ESM1-2-HR | 2.04 | 3.65 | 0.575 | 2.76 | 5.14 | 0.459 |
|  | MRI-ESM2-0 | 0.99 | -1.33 | 0.89583 | 1.42 | -2.44 | 0.765 |

**Table S4. Model performance metrics for minimum temperature across agroecological zones on Fold 1 validation (1999-2014) and Fold 2 validation (1983-1998)**

| **AEZ** | **Model** | **Fold 1** | | | **Fold 2** | | |
| --- | --- | --- | --- | --- | --- | --- | --- |
|  |  | **RMSE (°C/month)** | **PBIAS (%)** | **TSS** | **RMSE (°C/month)** | **PBIAS (%)** | **TSS** |
| EHAZ | AWI-CM-1-1-MR | 1.4 | 1.04 | 0.874 | 1.84 | 1.88 | 0.765 |
|  | GFDL-ESM4 | 1.81 | 3.82 | 0.852 | 2.51 | 5.22 | 0.715 |
|  | MIROC6 | 5.99 | 4.07 | 0.685 | 7.56 | 6.44 | 0.521 |
|  | MPI-ESM1-2-HR | 1.66 | 2.07 | 0.859 | 2.24 | 3.41 | 0.735 |
|  | MRI-ESM2-0 | 0.91 | 1.41 | 0.898 | 1.28 | 2.17 | 0.778 |
| EMAZ | AWI-CM-1-1-MR | 0.33 | 1.49 | 0.930 | 0.58 | 2.54 | 0.778 |
|  | GFDL-ESM4 | 0.62 | 2.97 | 0.892 | 0.88 | 4.11 | 0.732 |
|  | MIROC6 | 0.71 | 4.22 | 0.843 | 1.08 | 5.87 | 0.679 |
|  | MPI-ESM1-2-HR | 0.33 | 1.49 | 0.930 | 0.49 | 2.33 | 0.799 |
|  | MRI-ESM2-0 | 0.36 | 2.1 | 0.916 | 0.52 | 3.17 | 0.802 |
| THMAZ | AWI-CM-1-1-MR | 0.31 | 1.15 | 0.941 | 0.49 | 2.11 | 0.777 |
|  | GFDL-ESM4 | 0.61 | 2.56 | 0.911 | 0.84 | 3.77 | 0.753 |
|  | MIROC6 | 3.29 | 9.76 | 0.237 | 4.64 | 13.87 | 0.152 |
|  | MPI-ESM1-2-HR | 0.17 | 0.63 | 0.949 | 0.28 | 1.43 | 0.808 |
|  | MRI-ESM2-0 | 1.69 | 3.6 | 0.619 | 2.37 | 5.44 | 0.477 |
| ELAZ | AWI-CM-1-1-MR | 0.98 | -4.29 | 0.806 | 1.38 | -5.87 | 0.668 |
|  | GFDL-ESM4 | 0.43 | 1.79 | 0.939 | 0.61 | 2.43 | 0.823 |
|  | MIROC6 | 0.91 | 3.87 | 0.829 | 1.28 | 5.22 | 0.679 |
|  | MPI-ESM1-2-HR | 0.51 | -2.14 | 0.945 | 0.72 | -3.14 | 0.799 |
|  | MRI-ESM2-0 | 0.12 | -0.18 | 0.962 | 0.17 | -0.77 | 0.825 |
| EATZ | AWI-CM-1-1-MR | 0.71 | 1.69 | 0.777 | 0.99 | 2.77 | 0.648 |
|  | GFDL-ESM4 | 0.34 | 0.87 | 0.901 | 0.52 | 1.44 | 0.802 |
|  | MIROC6 | 1.33 | 3.53 | 0.740 | 1.74 | 4.88 | 0.600 |
|  | MPI-ESM1-2-HR | 0.37 | 0.85 | 0.888 | 0.56 | 1.67 | 0.782 |
|  | MRI-ESM2-0 | 0.79 | 2.21 | 0.945 | 1.03 | 3.14 | 0.823 |
| TLAZ | AWI-CM-1-1-MR | 2.84 | 4.14 | 0.766 | 3.78 | 5.88 | 0.610 |
|  | GFDL-ESM4 | 0.33 | -1.37 | 0.916 | 0.49 | -2.04 | 0.820 |
|  | MIROC6 | 2.22 | -3.14 | 0.663 | 3.14 | -4.54 | 0.523 |
|  | MPI-ESM1-2-HR | 1.44 | 2.58 | 0.920 | 1.99 | 3.77 | 0.760 |
|  | MRI-ESM2-0 | 0.49 | -2.18 | 0.945 | 0.68 | -3.11 | 0.823 |

**Table S5. Stability matrix for precipitation Fold 1 validation (1999-2014) and Fold 2 validation (1983-1998)**

| **AEZ** | **Model** | **Fold 1 Rank** | **Fold 2 Rank** | **Stability in top 3** |
| --- | --- | --- | --- | --- |
| EHAZ | BCC-CSM2-MR | 4 | 5 | No |
|  | CMCC-CM2-SR5 | 1 | 1 | Yes |
|  | GFDL-ESM4 | 3 | 3 | Yes |
|  | MIROC6 | 5 | 4 | No |
|  | MPI-ESM1-2-HR | 2 | 2 | Yes |
|  | MRI-ESM2-0 | 6 | 6 | No |
| EMAZ | BCC-CSM2-MR | 4 | 5 | No |
|  | CMCC-CM2-SR5 | 2 | 2 | Yes |
|  | GFDL-ESM4 | 3 | 3 | Yes |
|  | MIROC6 | 5 | 6 | No |
|  | MPI-ESM1-2-HR | 1 | 1 | Yes |
|  | MRI-ESM2-0 | 6 | 4 | No |
| THMAZ | BCC-CSM2-MR | 6 | 5 | No |
|  | CMCC-CM2-SR5 | 3 | 6 | No |
|  | GFDL-ESM4 | 2 | 2 | Yes |
|  | MIROC6 | 5 | 3 | No |
|  | MPI-ESM1-2-HR | 1 | 1 | Yes |
|  | MRI-ESM2-0 | 4 | 4 | No |
| ELAZ | BCC-CSM2-MR | 1 | 1 | Yes |
|  | CMCC-CM2-SR5 | 4 | 3 | No |
|  | GFDL-ESM4 | 3 | 4 | No |
|  | MIROC6 | 5 | 6 | No |
|  | MPI-ESM1-2-HR | 6 | 5 | No |
|  | MRI-ESM2-0 | 2 | 2 | Yes |
| EATZ | BCC-CSM2-MR | 4 | 5 | No |
|  | CMCC-CM2-SR5 | 1 | 2 | Yes |
|  | GFDL-ESM4 | 3 | 1 | Yes |
|  | MIROC6 | 5 | 4 | No |
|  | MPI-ESM1-2-HR | 6 | 6 | No |
|  | MRI-ESM2-0 | 2 | 3 | Yes |
| TLAZ | BCC-CSM2-MR | 1 | 1 | Yes |
|  | CMCC-CM2-SR5 | 3 | 4 | No |
|  | GFDL-ESM4 | 4 | 5 | No |
|  | MIROC6 | 5 | 6 | No |
|  | MPI-ESM1-2-HR | 2 | 3 | Yes |
|  | MRI-ESM2-0 | 6 | 2 | No |

**Table S6. Stability matrix for Tmax Fold 1 validation (1999-2014) and Fold 2 validation (1983-1998)**

| **AEZ** | **Model** | **Fold 1 Rank** | **Fold 2 Rank** | **Stability in top 3** |
| --- | --- | --- | --- | --- |
| EHAZ | AWI-CM-1-1-MR | 4 | 5 | No |
|  | GFDL-ESM4 | 3 | 3 | Yes |
|  | MIROC6 | 5 | 4 | No |
|  | MPI-ESM1-2-HR | 2 | 2 | Yes |
|  | MRI-ESM2-0 | 1 | 1 | Yes |
| EMAZ | AWI-CM-1-1-MR | 2 | 2 | Yes |
|  | GFDL-ESM4 | 3 | 3 | Yes |
|  | MIROC6 | 4 | 5 | No |
|  | MPI-ESM1-2-HR | 1 | 1 | Yes |
|  | MRI-ESM2-0 | 5 | 4 | No |
| THMAZ | AWI-CM-1-1-MR | 2 | 3 | Yes |
|  | GFDL-ESM4 | 3 | 1 | Yes |
|  | MIROC6 | 5 | 4 | No |
|  | MPI-ESM1-2-HR | 1 | 2 | Yes |
|  | MRI-ESM2-0 | 4 | 5 | No |
| ELAZ | AWI-CM-1-1-MR | 2 | 3 | Yes |
|  | GFDL-ESM4 | 3 | 4 | No |
|  | MIROC6 | 5 | 5 | No |
|  | MPI-ESM1-2-HR | 1 | 1 | Yes |
|  | MRI-ESM2-0 | 4 | 2 | No |
| EATZ | AWI-CM-1-1-MR | 4 | 4 | No |
|  | GFDL-ESM4 | 3 | 3 | Yes |
|  | MIROC6 | 5 | 5 | No |
|  | MPI-ESM1-2-HR | 1 | 1 | Yes |
|  | MRI-ESM2-0 | 2 | 2 | Yes |
| TLAZ | AWI-CM-1-1-MR | 4 | 4 | No |
|  | GFDL-ESM4 | 1 | 1 | Yes |
|  | MIROC6 | 3 | 3 | Yes |
|  | MPI-ESM1-2-HR | 5 | 5 | No |
|  | MRI-ESM2-0 | 2 | 2 | Yes |

**Table S7. Stability matrix for Tmin Fold 1 validation (1999-2014) and Fold 2 validation (1983-1998)**

| **AEZ** | **Model** | **Fold 1 Rank** | **Fold 2 Rank** | **Stability in top 3** |
| --- | --- | --- | --- | --- |
| EHAZ | AWI-CM-1-1-MR | 2 | 2 | Yes |
|  | GFDL-ESM4 | 4 | 4 | No |
|  | MIROC6 | 5 | 5 | No |
|  | MPI-ESM1-2-HR | 3 | 3 | Yes |
|  | MRI-ESM2-0 | 1 | 1 | Yes |
| EMAZ | AWI-CM-1-1-MR | 3 | 3 | Yes |
|  | GFDL-ESM4 | 5 | 4 | No |
|  | MIROC6 | 4 | 5 | No |
|  | MPI-ESM1-2-HR | 2 | 1 | Yes |
|  | MRI-ESM2-0 | 1 | 2 | Yes |
| THMAZ | AWI-CM-1-1-MR | 3 | 2 | Yes |
|  | GFDL-ESM4 | 2 | 3 | Yes |
|  | MIROC6 | 4 | 5 | No |
|  | MPI-ESM1-2-HR | 1 | 1 | Yes |
|  | MRI-ESM2-0 | 5 | 4 | No |
| ELAZ | AWI-CM-1-1-MR | 4 | 5 | No |
|  | GFDL-ESM4 | 1 | 1 | Yes |
|  | MIROC6 | 5 | 4 | No |
|  | MPI-ESM1-2-HR | 2 | 3 | Yes |
|  | MRI-ESM2-0 | 3 | 2 | Yes |
| EATZ | AWI-CM-1-1-MR | 4 | 4 | No |
|  | GFDL-ESM4 | 2 | 2 | Yes |
|  | MIROC6 | 5 | 5 | No |
|  | MPI-ESM1-2-HR | 3 | 3 | Yes |
|  | MRI-ESM2-0 | 1 | 1 | Yes |
| TLAZ | AWI-CM-1-1-MR | 4 | 4 | No |
|  | GFDL-ESM4 | 2 | 2 | Yes |
|  | MIROC6 | 5 | 5 | No |
|  | MPI-ESM1-2-HR | 3 | 3 | Yes |
|  | MRI-ESM2-0 | 1 | 1 | Yes |

**Table S8. Stability index for precipitation**

| **Model** | **Stability Index** | **Fraction in Top 3** |
| --- | --- | --- |
| MPI-ESM1-2-HR | 1.00 | 4/4 |
| MRI-ESM2-0 | 1.00 | 3/3 |
| BCC-CSM2-MR | 1.00 | 2/2 |
| GFDL-ESM4 | 0.75 | 3/4 |
| CMCC-CM2-SR5 | 0.60 | 3/5 |
| Average SI | 0.83 | 15/18 |

**Table S9. Stability index for Temperature**

| **Model** | **Tmax** | | **Tmin** | |
| --- | --- | --- | --- | --- |
|  | **Stability index** | **Fraction in Top 3** | **SI** | **Fraction in Top 3** |
| MPI-ESM1-2-HR | 1.00 | 5/5 | 1.00 | 6/6 |
| AWI-CM-1-1-MR | 1.00 | 4/4 | 1.00 | 2/2 |
| MRI-ESM2-0 | 1.00 | 3/3 | 1.00 | 5/5 |
| GFDL-ESM4 | 0.83 | 4/5 | 1.00 | 5/5 |
| MIROC6 | 1.00 | 1/1 | - |  |
| Average SI | 0.944 | 17/18 | 1 | 18/18 |

**TABLE S10. Sensitivity of Precipitation Top 3 vs Top 5 ensemble members**

| **Scenario** | **AEZs** | **2026-2050** | | |  | **2051-2075** | | |  | **2076-2100** | | |
| --- | --- | --- | --- | --- | --- | --- | --- | --- | --- | --- | --- | --- |
|  |  | **Top 3**  **(%)** | **Top 5 (%)** | **Diff (%)** |  | **Top 3**  **(%)** | **Top 5 (%)** | **Diff (%)** |  | **Top 3**  **(%)** | **Top 5 (%)** | **Diff (%)** |
| SSP2-4.5 | EHAZ | -7.8 | -10.42 | -2.62 |  | -3 | -5.51 | -2.51 |  | -0.6 | -2.94 | -2.34 |
|  | EMAZ | -9.4 | -11.62 | -2.22 |  | -10.1 | -12.78 | -2.68 |  | -9.8 | -12.15 | -2.35 |
|  | THMAZ | 0.9 | -3.12 | -4.02 |  | -3.5 | -8.21 | -4.71 |  | -5.7 | -10.84 | -5.14 |
|  | ELAZ | -2.5 | -4.31 | -1.81 |  | -0.9 | -3.12 | -2.22 |  | 0.6 | -1.85 | -2.45 |
|  | EATZ | -2.1 | -1.75 | 0.35 |  | 0.3 | 0.48 | 0.18 |  | 0.7 | 1.05 | 0.35 |
|  | TLAZ | -2.6 | -4.82 | -2.22 |  | -0.5 | -2.91 | -2.41 |  | 1.8 | -0.74 | -2.54 |
| SSP3-7.0 | EHAZ | 0.1 | -2.15 | -2.25 |  | -4.1 | -6.84 | -2.74 |  | 8.9 | 5.62 | -3.28 |
|  | EMAZ | -1.8 | -3.95 | -2.15 |  | -9.2 | -11.44 | -2.24 |  | -0.8 | -3.22 | -2.42 |
|  | THMAZ | 0.8 | -4.25 | -5.05 |  | -4.9 | -10.15 | -5.25 |  | -7.1 | -12.33 | -5.23 |
|  | ELAZ | -0.5 | -2.18 | -1.68 |  | -1.1 | -3.47 | -2.37 |  | 1.2 | -0.74 | -1.94 |
|  | EATZ | 0.7 | 0.92 | 0.22 |  | -0.3 | -0.15 | 0.15 |  | 2.6 | 2.91 | 0.31 |
|  | TLAZ | -1.6 | -3.25 | -1.65 |  | -2.3 | -4.68 | -2.38 |  | 2.9 | 0.52 | -2.38 |
| SSP5-8.5 | EHAZ | -4.6 | -6.83 | -2.23 |  | -1.9 | -4.27 | -2.37 |  | 9.5 | 6.31 | -3.19 |
|  | EMAZ | -11.3 | -13.51 | -2.21 |  | -7.4 | -9.82 | -2.42 |  | -3.2 | -5.76 | -2.56 |
|  | THMAZ | -1.9 | -6.44 | -4.54 |  | 2.2 | -2.63 | -4.83 |  | 21.2 | 15.42 | -5.78 |
|  | ELAZ | -1.5 | -3.42 | -1.92 |  | 2.3 | 0.15 | -2.15 |  | 6.9 | 4.33 | -2.57 |
|  | EATZ | -0.3 | -0.11 | 0.19 |  | 1.9 | 2.12 | 0.22 |  | 2.6 | 2.84 | 0.24 |
|  | TLAZ | -2.5 | -4.71 | -2.21 |  | 0.9 | -1.45 | -2.35 |  | 2.6 | 0.18 | -2.42 |

**Table S11. Sensitivity of Tmin Top 3 vs Top 5 ensemble members**

| **Scenario** | **AEZs** | **2026-2050** | | |  | **2051-2075** | | |  | **2076-2100** | | |
| --- | --- | --- | --- | --- | --- | --- | --- | --- | --- | --- | --- | --- |
|  |  | **Top 3**  **(°C)** | **Top 5 (°C)** | **Diff (°C)** |  | **Top 3**  **(°C)** | **Top 5 (°C)** | **Diff (°C)** |  | **Top 3**  **(°C)** | **Top 5 (°C)** | **Diff (°C)** |
| SSP2-4.5 | EHAZ | 1.29 | 1.51 | 0.22 |  | 1.91 | 2.12 | 0.21 |  | 2.29 | 2.52 | 0.23 |
|  | EMAZ | 1.25 | 1.33 | 0.08 |  | 2 | 2.12 | 0.12 |  | 2.41 | 2.53 | 0.12 |
|  | THMAZ | 1.72 | 2.34 | 0.62 |  | 2.34 | 2.92 | 0.58 |  | 2.68 | 3.29 | 0.61 |
|  | ELAZ | 0.31 | 0.42 | 0.11 |  | 0.95 | 1.04 | 0.09 |  | 1.33 | 1.45 | 0.12 |
|  | EATZ | 1.34 | 1.45 | 0.11 |  | 1.95 | 2.07 | 0.12 |  | 2.36 | 2.48 | 0.12 |
|  | TLAZ | 0.58 | 0.67 | 0.09 |  | 1.23 | 1.34 | 0.11 |  | 1.67 | 1.78 | 0.11 |
| SSP3-7.0 | EHAZ | 1.36 | 1.56 | 0.2 |  | 2.28 | 2.47 | 0.19 |  | 3.04 | 3.24 | 0.2 |
|  | EMAZ | 1.37 | 1.46 | 0.09 |  | 2.35 | 2.44 | 0.09 |  | 3.23 | 3.31 | 0.08 |
|  | THMAZ | 1.86 | 2.45 | 0.59 |  | 2.85 | 3.46 | 0.61 |  | 3.83 | 4.42 | 0.59 |
|  | ELAZ | 0.42 | 0.54 | 0.12 |  | 1.58 | 1.69 | 0.11 |  | 2.87 | 2.96 | 0.09 |
|  | EATZ | 1.47 | 1.56 | 0.09 |  | 2.45 | 2.54 | 0.09 |  | 3.46 | 3.59 | 0.13 |
|  | TLAZ | 0.71 | 0.82 | 0.11 |  | 1.79 | 1.91 | 0.12 |  | 3.09 | 3.21 | 0.12 |
| SSP5-8.5 | EHAZ | 1.47 | 1.69 | 0.22 |  | 2.33 | 2.55 | 0.22 |  | 3.48 | 3.67 | 0.19 |
|  | EMAZ | 1.35 | 1.42 | 0.07 |  | 2.28 | 2.39 | 0.11 |  | 3.58 | 3.69 | 0.11 |
|  | THMAZ | 1.79 | 2.41 | 0.62 |  | 2.65 | 3.24 | 0.59 |  | 3.89 | 4.51 | 0.62 |
|  | ELAZ | 0.49 | 0.58 | 0.09 |  | 1.49 | 1.61 | 0.12 |  | 2.79 | 2.91 | 0.12 |
|  | EATZ | 1.59 | 1.68 | 0.09 |  | 2.61 | 2.72 | 0.11 |  | 3.83 | 3.94 | 0.11 |
|  | TLAZ | 0.83 | 0.95 | 0.12 |  | 1.92 | 2.01 | 0.09 |  | 3.23 | 3.36 | 0.13 |

**Table S12. Sensitivity of Tmax Top 3 vs Top 5 ensemble members**

| **Scenario** | **AEZs** | **2026-2050** | | |  | **2051-2075** | | |  | **2076-2100** | | |
| --- | --- | --- | --- | --- | --- | --- | --- | --- | --- | --- | --- | --- |
|  |  | **Top 3**  **(°C)** | **Top 5 (°C)** | **Diff (°C)** |  | **Top 3**  **(°C)** | **Top 5 (°C)** | **Diff (°C)** |  | **Top 3**  **(°C)** | **Top 5 (°C)** | **Diff (°C)** |
| SSP2-4.5 | EHAZ | 0.74 | 1.16 | 0.42 |  | 1.28 | 1.72 | 0.44 |  | 1.59 | 2.04 | 0.45 |
|  | EMAZ | 1.39 | 1.58 | 0.19 |  | 2.17 | 2.38 | 0.21 |  | 2.58 | 2.74 | 0.16 |
|  | THMAZ | 1.47 | 1.64 | 0.17 |  | 2.08 | 2.22 | 0.14 |  | 2.38 | 2.55 | 0.17 |
|  | ELAZ | 0.07 | -0.41 | -0.48 |  | 0.75 | 0.19 | -0.56 |  | 1.24 | 0.68 | -0.56 |
|  | EATZ | 0.85 | 1.01 | 0.16 |  | 1.47 | 1.61 | 0.14 |  | 1.95 | 2.16 | 0.21 |
|  | TLAZ | 0.1 | 0.96 | 0.86 |  | 0.7 | 1.56 | 0.86 |  | 1.11 | 2.04 | 0.93 |
| SSP3-7.0 | EHAZ | 0.76 | 1.21 | 0.45 |  | 1.49 | 1.94 | 0.45 |  | 1.98 | 2.46 | 0.48 |
|  | EMAZ | 1.46 | 1.69 | 0.23 |  | 2.43 | 2.61 | 0.18 |  | 3.17 | 3.41 | 0.24 |
|  | THMAZ | 1.54 | 1.71 | 0.17 |  | 2.32 | 2.48 | 0.16 |  | 2.97 | 3.11 | 0.14 |
|  | ELAZ | 0.02 | -0.56 | -0.58 |  | 1.04 | 0.46 | -0.58 |  | 2.09 | 1.52 | -0.57 |
|  | EATZ | 0.93 | 1.12 | 0.19 |  | 2.02 | 2.21 | 0.19 |  | 2.94 | 3.09 | 0.15 |
|  | TLAZ | 0.22 | 1.14 | 0.92 |  | 1.25 | 2.18 | 0.93 |  | 2.38 | 3.21 | 0.83 |
| SSP5-8.5 | EHAZ | 0.79 | 1.28 | 0.49 |  | 1.35 | 1.81 | 0.46 |  | 2.1 | 2.57 | 0.47 |
|  | EMAZ | 1.46 | 1.65 | 0.19 |  | 2.24 | 2.49 | 0.25 |  | 3.38 | 3.56 | 0.18 |
|  | THMAZ | 1.49 | 1.62 | 0.13 |  | 2.02 | 2.19 | 0.17 |  | 2.97 | 3.18 | 0.21 |
|  | ELAZ | 0.15 | -0.34 | -0.49 |  | 0.97 | 0.41 | -0.56 |  | 2.31 | 1.77 | -0.54 |
|  | EATZ | 1.06 | 1.21 | 0.15 |  | 1.9 | 2.11 | 0.21 |  | 3.08 | 3.27 | 0.19 |
|  | TLAZ | 0.25 | 1.11 | 0.86 |  | 1.03 | 1.89 | 0.86 |  | 2.25 | 3.16 | 0.91 |

**Supplementary Figures**


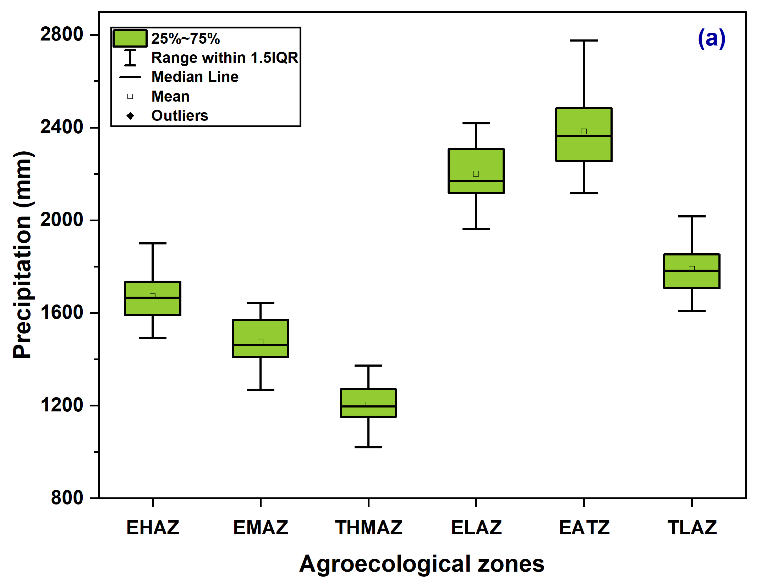

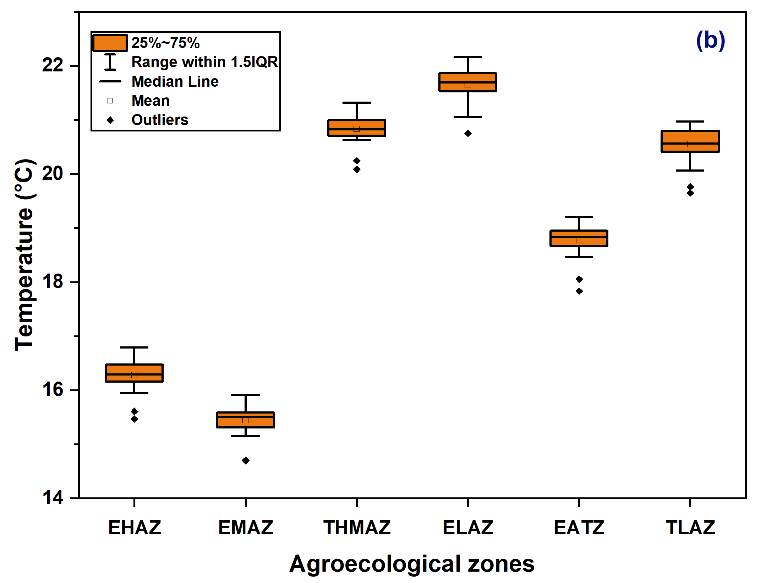

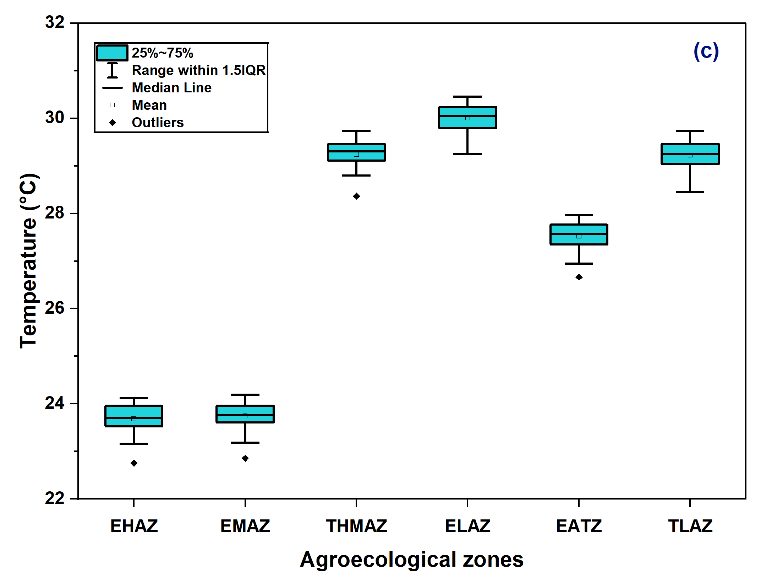


**Figure S1. Box-Whisker plot of annual daily average (a) precipitation, (b) Tmin and (c) Tmin across AEZs.**


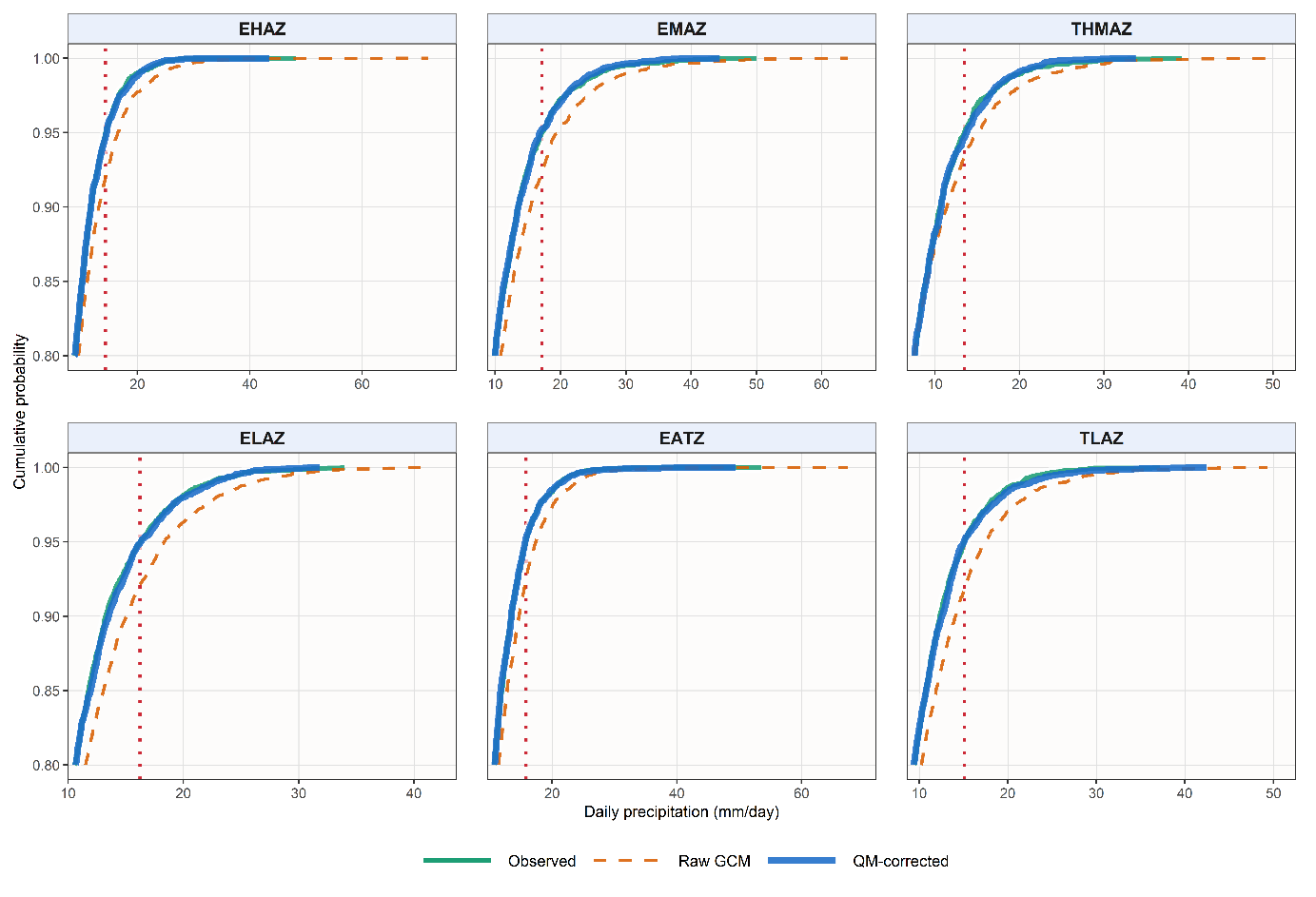


**Figure S2. Cumulative distribution function (CDF) of daily precipitation for observed, raw ensemble mean, and bias-corrected (QM) ensemble mean during the validation period (1999–2014)**


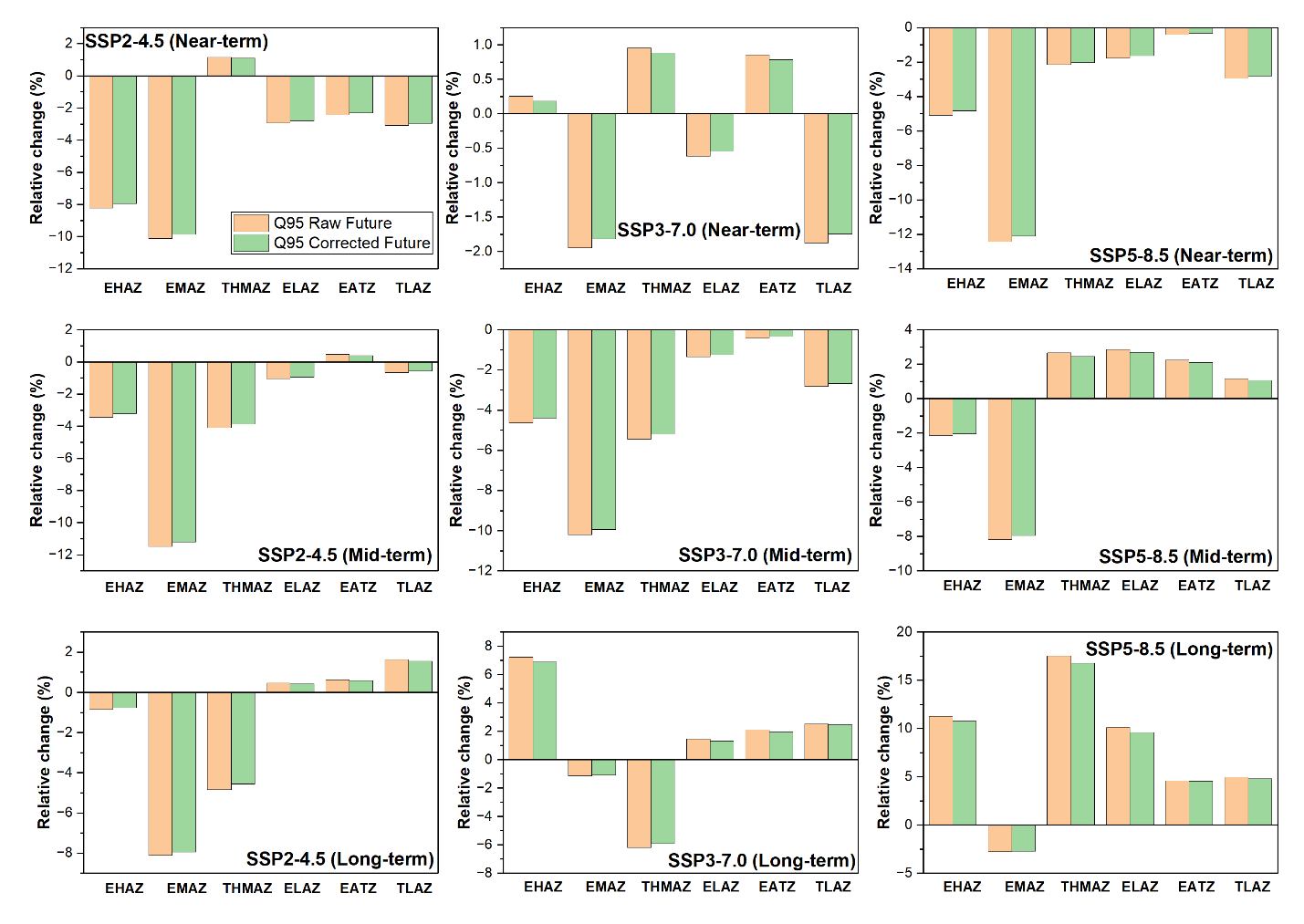


**Figure S3. Comparison of relative climate change signals between Raw GCM and QM corrected data for the 95th precipitation quantile across three SSPs under near, mid and long-term. Relative change is calculated against the 1999–2014 validation period.**


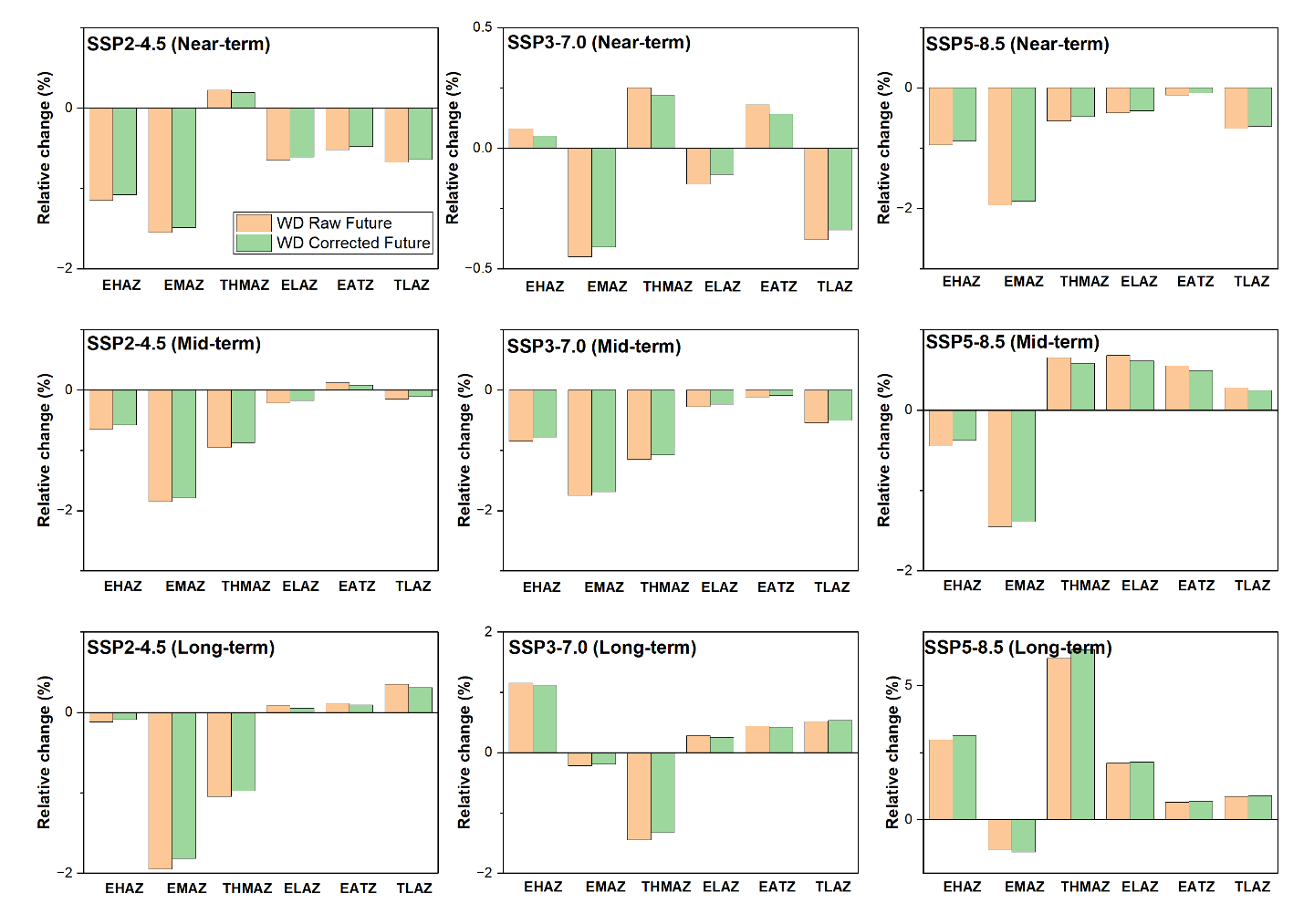


**Figure S4. Comparison of relative climate change signals between Raw GCM and QM corrected data for the wet day across three SSPs under near, mid and long-term. Relative change is calculated against the 1999–2014 validation period.**


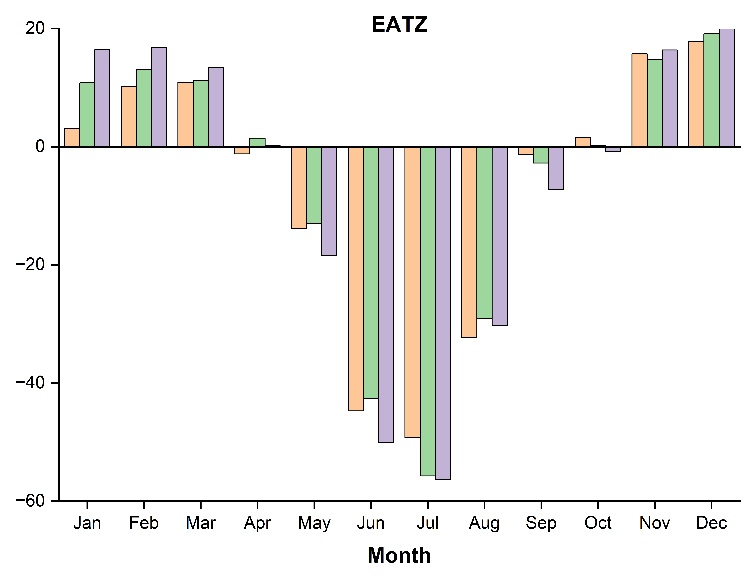

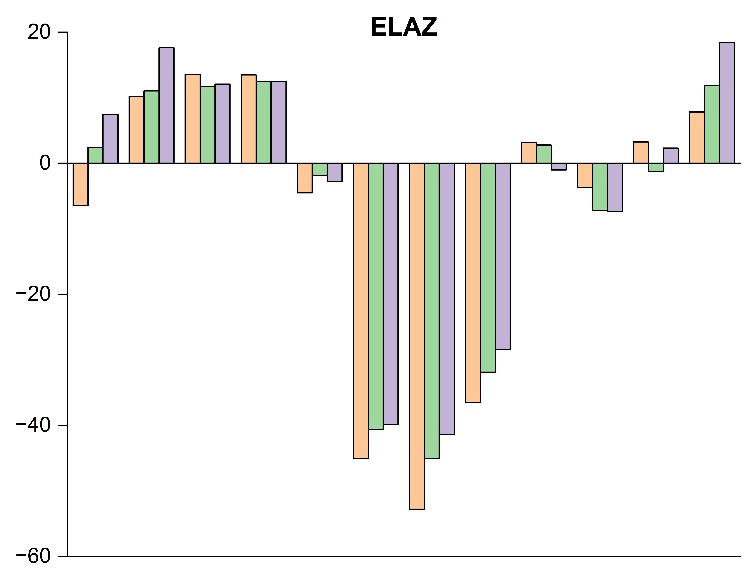

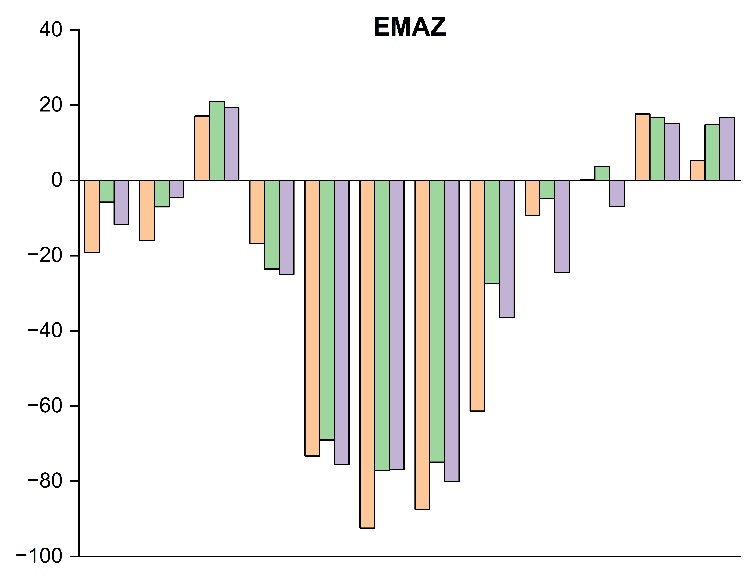

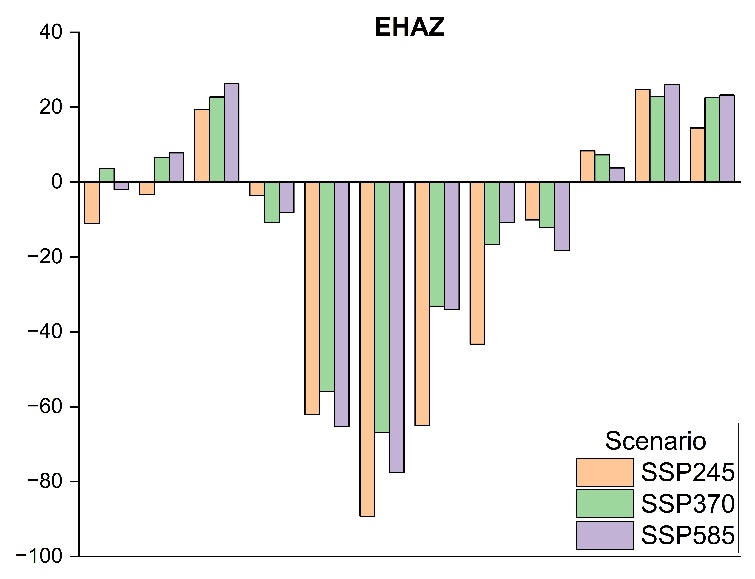

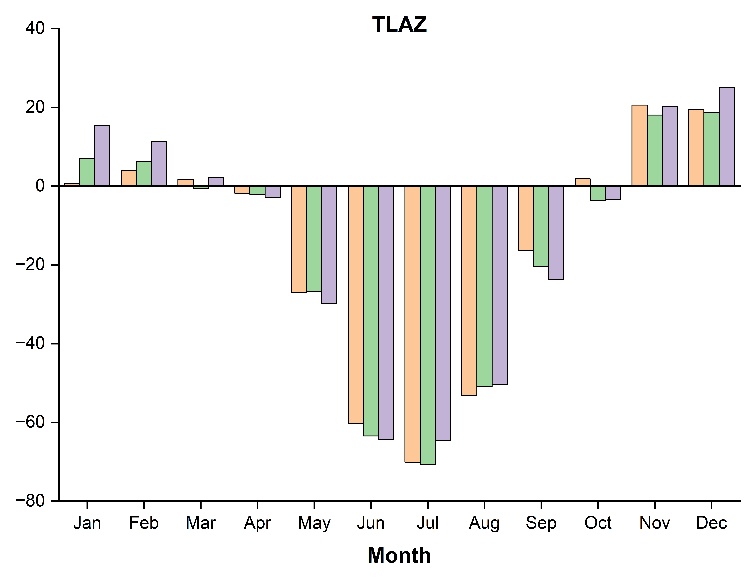

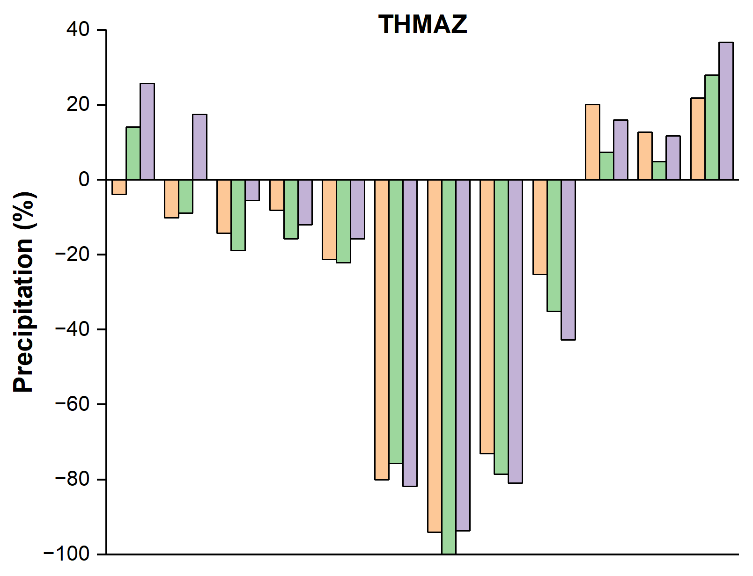


**Figure S5**. **Mean monthly precipitation changes (%) in period of 2014–2100 under SSP2–4.5, SSP3–7.0, and SSP5–8.5.**


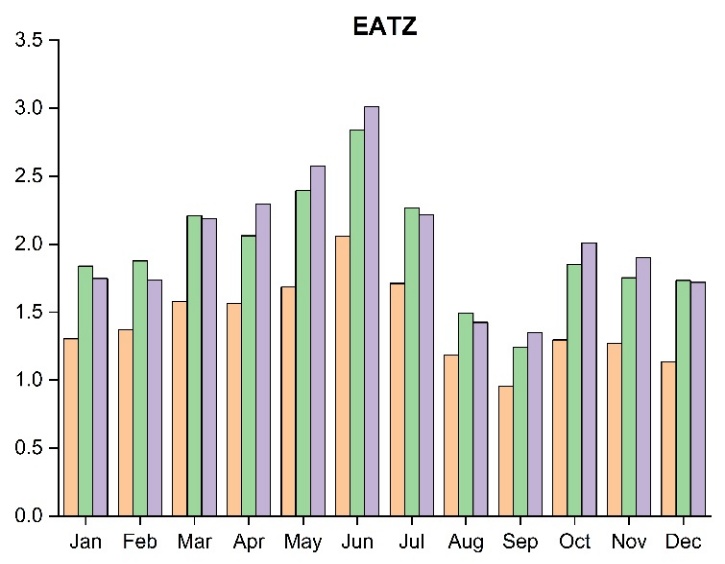

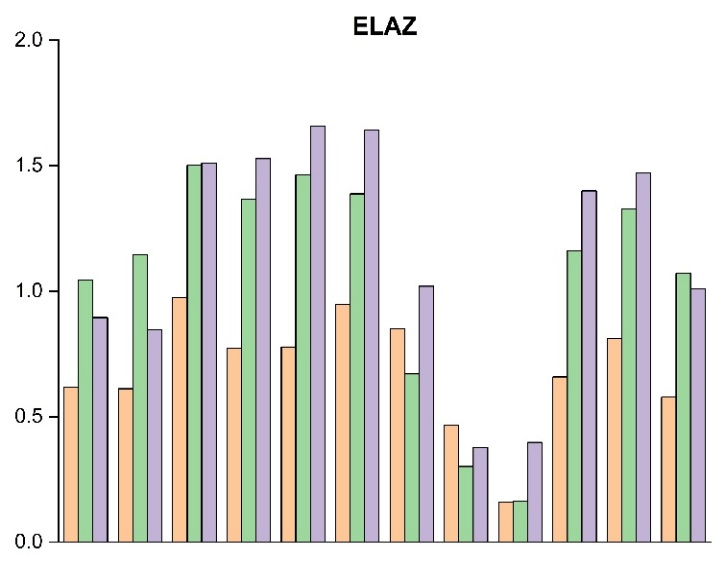

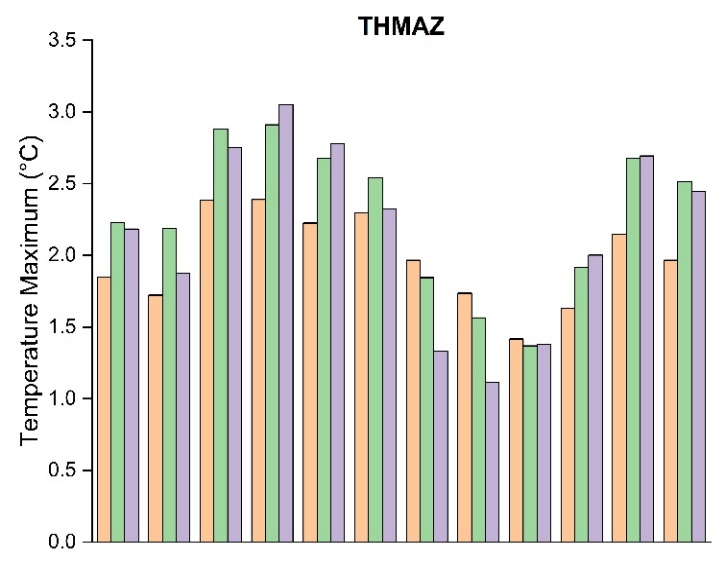

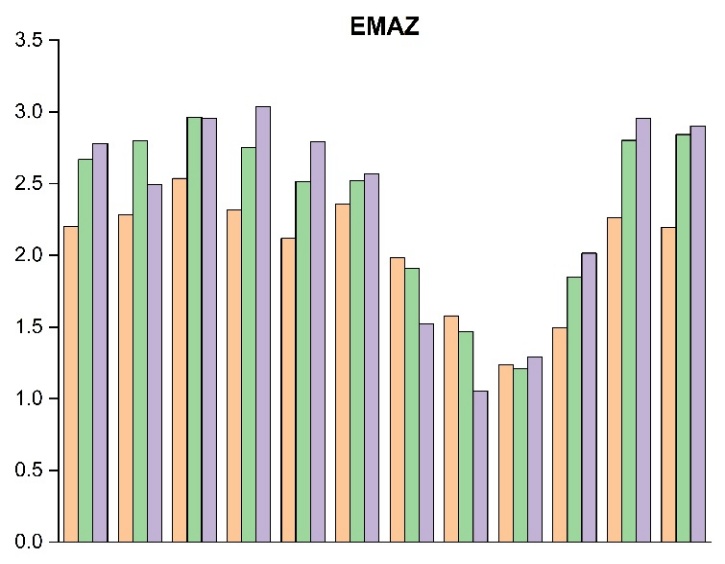

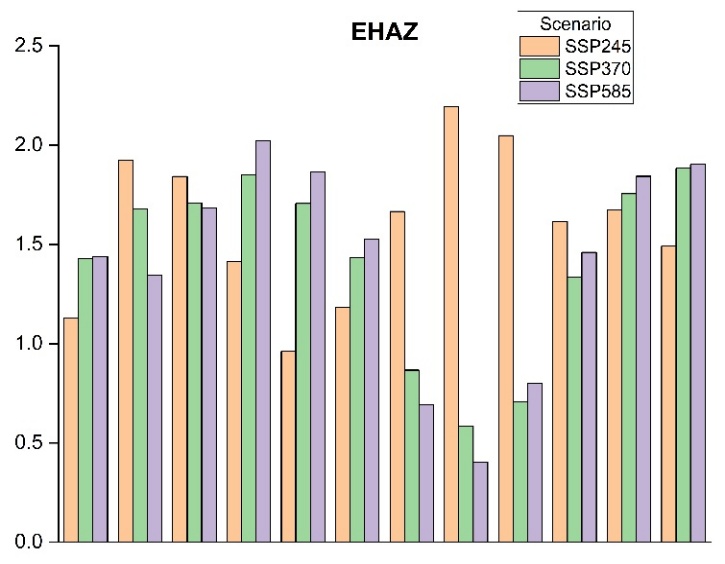

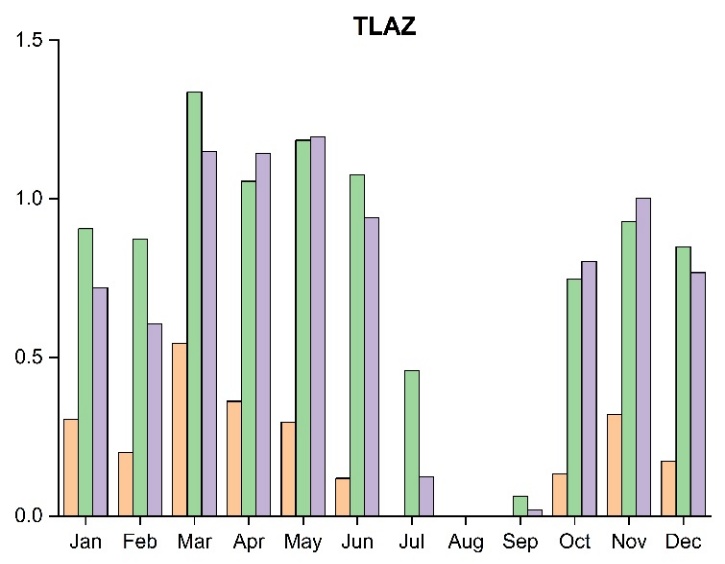


**Figure S6. Mean monthly Tmax changes (°C) in periods of 2014–2100 under SSP2–4.5, SSP3–7.0, and SSP5–8.5.**


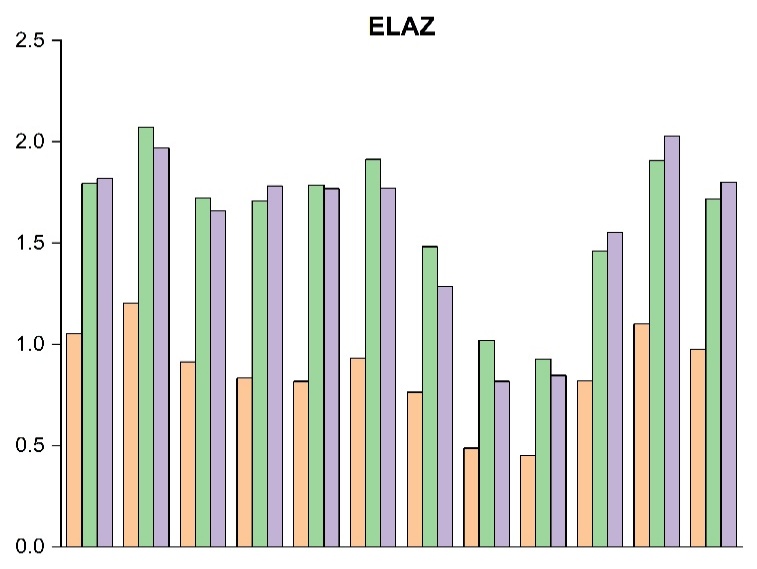

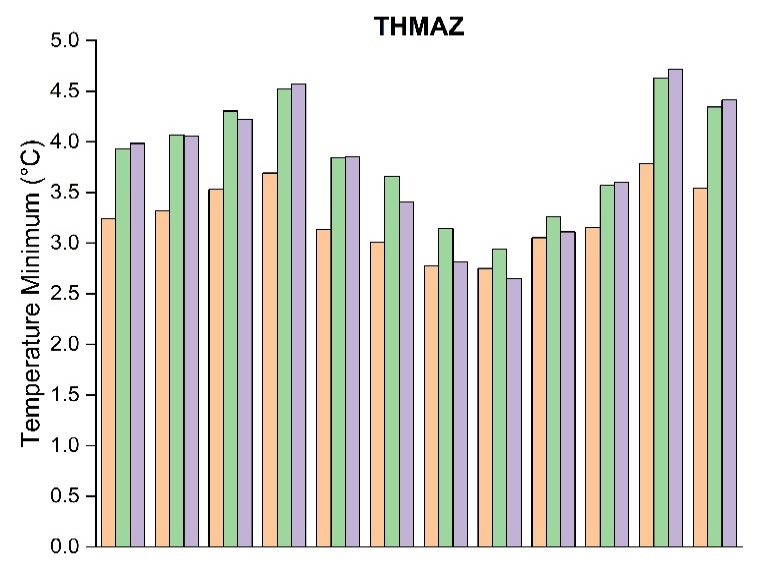

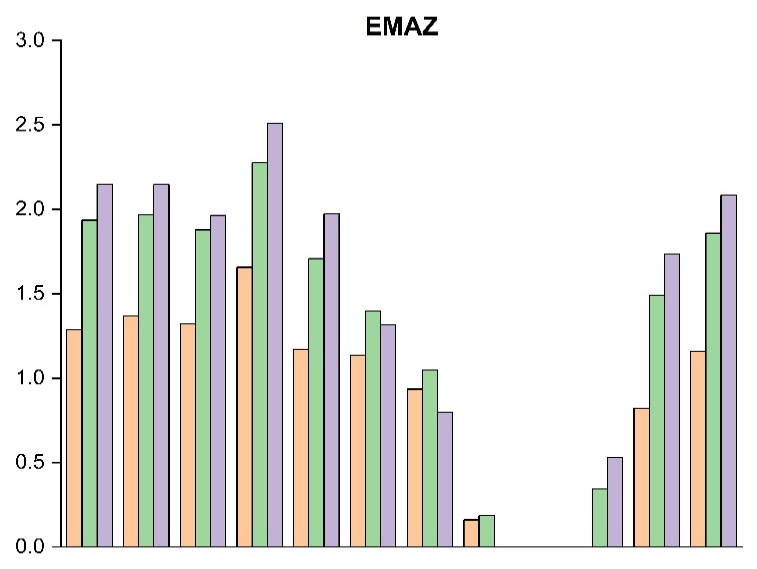

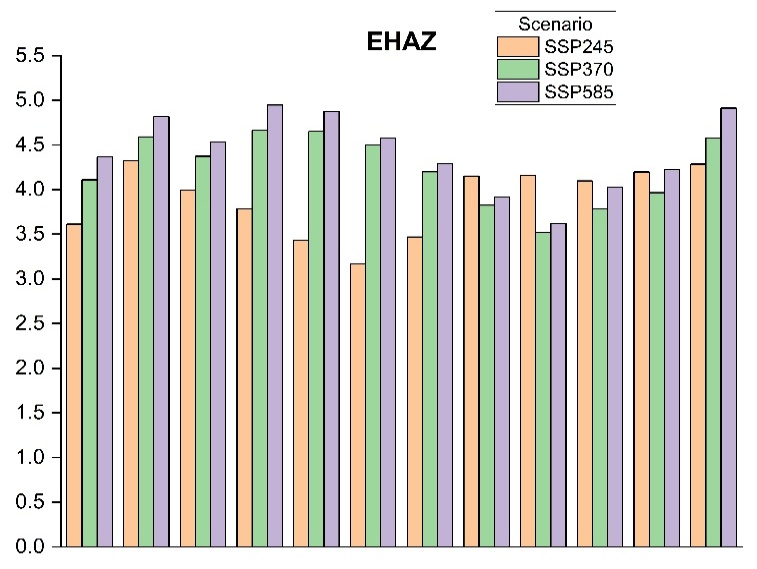

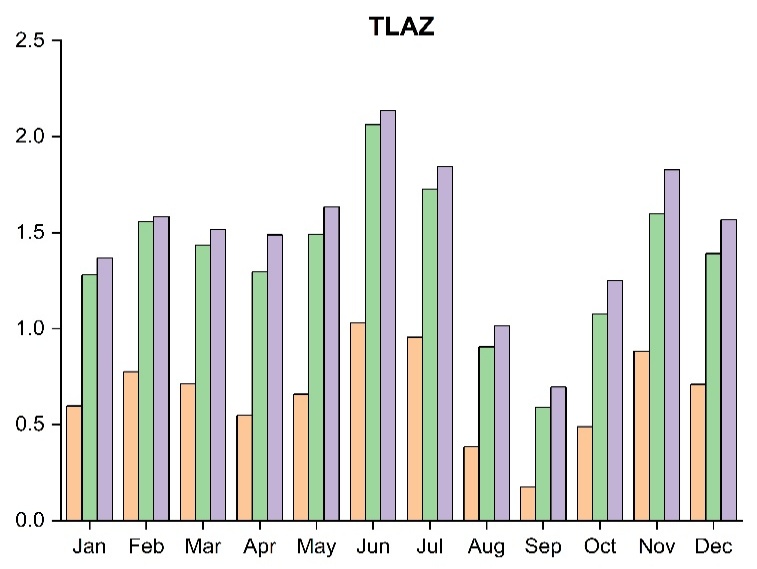

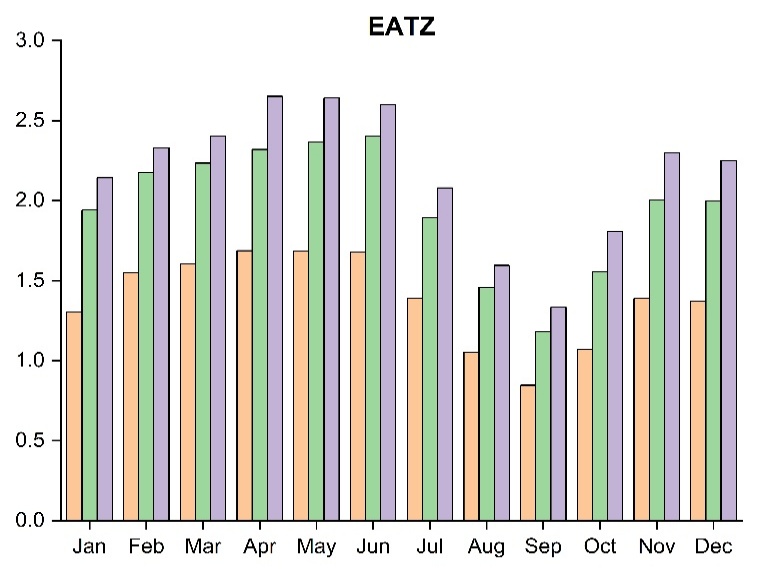


**Figure S7. Mean monthly Tmin changes (°C) in periods of 2014–2100 under SSP2–4.5, SSP3–7.0, and SSP5–8.5.**
